# Design, Synthesis, and Biological Evaluation of a Novel VEGFR-2 Inhibitor Based on a 1,2,5-Oxadiazole-2-Oxide Scaffold with MAPK Signaling Pathway Inhibition

**DOI:** 10.3390/ph15020246

**Published:** 2022-02-18

**Authors:** Mater H. Mahnashi, Fardous F. El-Senduny, Mohammed Abdulrahman Alshahrani, Mahrous A. Abou-Salim

**Affiliations:** 1 Department of Pharmaceutical Chemistry, College of Pharmacy, Najran University, Najran 61441, Saudi Arabia; mhmahneshi@nu.edu.sa; 2 Chemistry Department, Faculty of Science, Mansoura University, Mansoura 35516, Egypt; fkaneer@mans.edu.eg; 3 Department of Clinical Laboratory Sciences, Faculty of Applied Medical Sciences, Najran University, Najran 61441, Saudi Arabia; maalshahrani@nu.edu.sa; 4 Pharmaceutical Organic Chemistry, Faculty of Pharmacy, Al-Azhar University, Assiut 71524, Egypt

**Keywords:** pyrazolo[3,4-d]pyrimidines, furoxan, VEGFR-2, DAF-FM DA, MAPK, metastasis, apoptosis

## Abstract

Over the past few decades, the development of broad-spectrum anticancer agents with anti-angiogenic activity has witnessed considerable progress. In this study, a new series of pyrazolo[3,4-d]pyrimidines based on a phenylfuroxan scaffold were designed, synthesized, and evaluated, in terms of their anticancer activities. NCI-60 cell one-dose screening revealed that compounds **12a**–**c** and **14a** had the best MGI%, among the tested compounds. The target fluorinated compound **12b**, as the most active one, showed better anticancer activity compared to the reference drug sorafenib, with IC_50_ values of 11.5, 11.6, and 13 µM against the HepG-2, A2780CP, and MDA-MB-231 cell lines, respectively. Furthermore, compound **12b** (IC_50_ = 0.092 µM) had VEGFR-2-inhibitory activity comparable to that of the standard inhibitor sorafenib (IC_50_ = 0.049 µM). Furthermore, the ability of compound **12b** in modulating MAPK signaling pathways was investigated. It was found to decrease the level of total ERK and its phosphorylated form, as well as leading to the down-regulation of metalloproteinase MMP-9 and the over-expression of p21 and p27, thus leading to subG1 cell-cycle arrest and, thus, the induction of apoptosis. Additionally, compound **12b** decreased the rate of wound healing in the absence of serum, in comparison to DMSO-treated cells, providing a significant impact on metastasis inhibition. The quantitative RT-PCR results for *E-cadherin* and *N-cadherin* showed lower expression of the neuronal *N-cadherin* and increased expression of epithelial *E-cadherin*, indicating the ability of **12b** to suppress metastasis. Furthermore, **12b**-treated HepG2 cells expressed a low level of anti-apoptotic *BCL-2* and over-expressed proapoptotic *Bax* genes, respectively. Using the DAF-FM DA fluorescence probe, compound **12b** produced NO intracellularly as efficiently as the reference drug JS-K. In silico molecular docking studies showed a structural similarity through an overlay of **12b** with sorafenib. Interestingly, the drug-likeness properties of compound **12b** met the expectations of Pfizer’s rule for the design of new drug candidates. Therefore, this study presents a novel anticancer lead compound that is worthy of further investigation and activity improvement.

## 1. Introduction

Cancer is a large group of diseases, and is the result of the uncontrollable growth of abnormal cells [1]. In 2018, according to the WHO, the second leading cause of death worldwide was cancer, and it was estimated that 9.6 million people worldwide died from various cancers [1]. About 300,000 new cases are diagnosed every year among children only. The total annual economic cost of cancer was estimated to be about $1.16 trillion per year, and this number has been rising [2]. Cancer affects everyone—men, women, and children—alike, and represents an enormous burden for all. Cancer not only affects the diseased organ, but also invades adjoining organs in a process called metastasis, which is a major cause of death from cancer [1]. The interaction of tumor cells with their microenvironment is essential for their development [3]. Angiogenesis is the key regulatory process for tumor growth and metastasis, which begins with splitting or sprouting pre-existing blood vessels to form new ones, in order to deliver the essential nutrients to the tumor, and is a focus of anticancer therapy [3,4,5,6]. This process involves the growth, migration, and differentiation of blood vessel endothelial cells, which line their inside wall [7]. Therefore, tumor growth and metastasis may be suppressed by anti-angiogenic agents.

Vascular endothelial growth factor (VEGF) expression, through the expression of the cell-surface kinase VEGFR-2 (KDR/Flk-1), is strongly associated with both angiogenesis and tumor aggressiveness [5,8]. KDR is overexpressed in different types of cancers, such as breast cancer [9,10], cervical cancer [11,12], non-small cell lung cancer (NSCLC) [11,13,14], hepatocellular carcinoma (HCC) [5,11,15], and renal carcinoma [5,11,16]. VEGF overexpression has been observed in solid tumors, which initiates the activation of VEGFR-2 [4]. This, in turn, triggers a downstream signaling cascade, including the Raf/MEK/ERK pathway, ultimately leading to the excessive formation of new blood vessels and accelerated angiogenesis, tumor proliferation, and metastasis [4,5,17,18]. Hence, effective anti-angiogenic agents are those having potential inhibition of VEGFR-2 [4,18].

Sunitinib, sorafenib, and cabozantinib were recently shown to be among the anti-angiogenic drugs that can inhibit VEGFR-2, and which are active against a vast array of cancer types [4]. Their adverse side-effects and influence on patient health are the main cause of concern relating to chemotherapeutic drugs [19]. Therefore, there is an urgent need to develop new classes of VEGFR-2 inhibitors with low toxicity and enriched anti-angiogenic potential.

The chemical fragments pyrazolo [3,4-d]pyrimidine and 1,2,5-oxadiazole-*N*-oxide may serve as promising scaffolds for the design of a new class of anti-angiogenic agents through improved bioavailability and enhanced biological activity [20,21,22]. They have been identified based on the combination principle of multiple pharmacophore and bioisosteric replacements of the lead potent multi-target kinase inhibitor sorafenib (Nexavar^®^) [23,24]. Sorafenib is a small molecule inhibitor of VEGFR-2, VEGFR-3, PDGFR-ß, and c-Kit kinases with downstream targeting of the Raf/MEK/ERK pathway [23,25].

**Figure 1:**
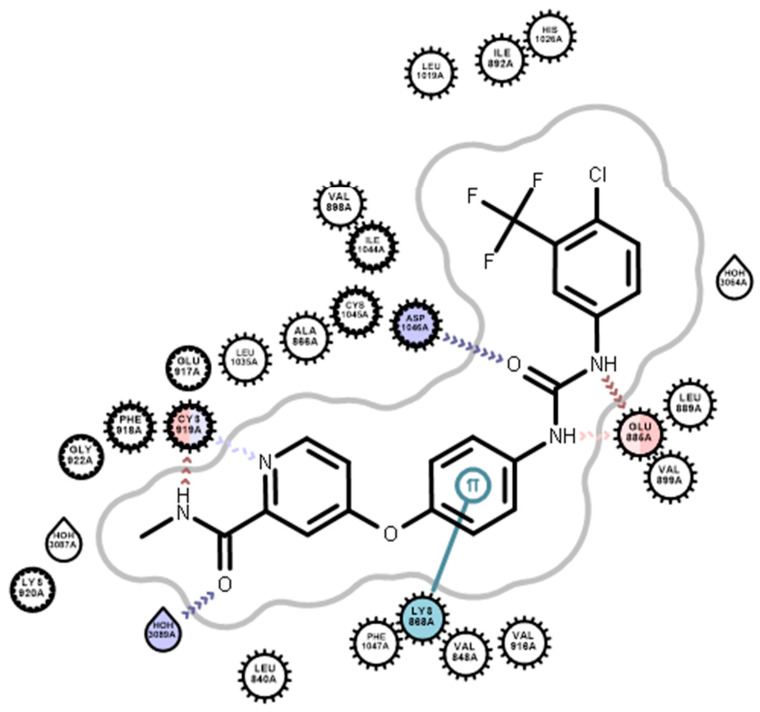
Basic pharmacophoric features of sorafenib (PDB ID: 3WZE).

**Figure 2:**
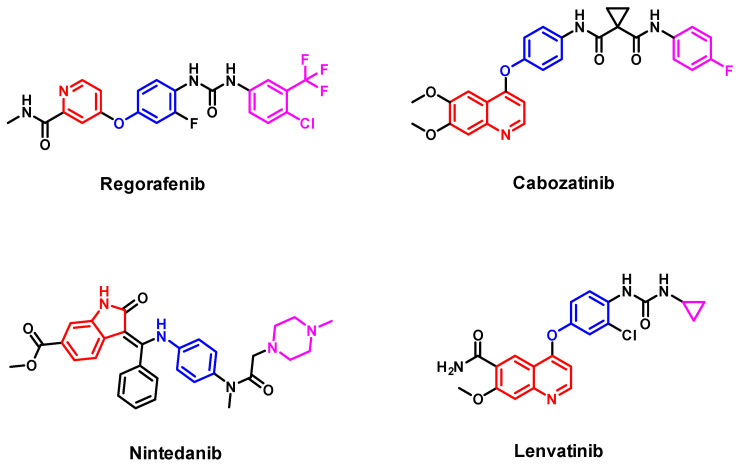
Pharmacophoric features of the next-generation VEGFR-2 inhibitors.

The basic pharmacophoric features of sorafenib are characterized by its hydrophobic tail (allosteric binding site; substituted phenyl), hydrogen bond donor and acceptor activities (binding with Glu:883:A and Asp:1044:A in the DFG domain; urea group), central aryl ring (occupying the linker region; aryloxy group), and heteroaromatic ring (ATB-binding domain; substituted pyridine) [26,27,28]; see Figure 1. Interestingly, the next generation of potent VEGFR-2 inhibitors such as regorafenib [28], cabozantinib [28], nintedanib [28] and lenvatinib [26,28] demonstrate the same basic pharmacophoric features of sorafenib [26,27,28]: hydrophobic tail (substituted phenyl, piperazinyl, or cyclopropyl), hydrogen bond donor and acceptor activities (urea or amide group), central aryl ring (aryloxy or arylamino group), and heteroaromatic ring (substituted pyridine, quinoline, or indole); see Figure 2. Hence, the design of a new series with these essential structural pharmacophoric features has drawn our interest.

Furthermore, the pyrimidine-based core was selected as the ATP-binding domain isostere. It has recently been reported that numerous pyrazolo[3,4-d]pyrimidine derivatives exhibit potent anti-tumor activities [29,30,31,32]. In 2018, Wand et al. reported a potent VEGFR-2 inhibitor having a pyrazolo[3,4-d]pyrimidine scaffold [20]. Therefore, the privileged pyrazolo[3,4-d]pyrimidine nucleus was deemed to be a promising scaffold for developing a new VEGFR-2 inhibitor class.

On the other hand, the nitric oxide (NO)-releasing furoxan moiety was shown to have potent anti-tumor activity in earlier reports [21,22]. It is worth mentioning that NO plays a pivotal role as a signaling free radical molecule, functioning as a vascular smooth muscle relaxant, platelet aggregation inhibitor, neurotransmitter, and immune regulator [33]. The anti-tumor activity of NO has been observed at high levels (more than 300 nM), through the initiation of DNA damage and nitrosative stress [34,35], the phosphorylation of P53, and the increasing of MKP-1 expression, leading to cellular respiration inhibition. High levels of NO also play a crucial role in the reversal of drug resistance through MRP efflux pump inhibition [36,37], hypoxia-induced drug-resistance attenuation [36], hypoxic radio-sensitization [38], transcription factor hypoxia-inducible factor (HIF-1) activation blocking [36,38], inhibition of epithelial–mesenchymal transition (EMT) [39], and metastatic inhibition [39]. Therefore, increasing the concentration of intracellular NO may have a significant impact on tumor growth and metastasis [35,36].

A convenient way to achieve high levels of intracellular NO is through the use of NO donors [40]. Among them, the most stable is the furoxan moiety [41], which releases NO in the presence of plasma, reduced glutathione (GSH), or albumin (i.e., through thiol mechanisms) and, therefore, may exert better biological activity and rarely leads to tolerance, compared to other nitric oxide-releasing motifs [40,41]. In summary, the synergistic effects discussed above have raised hopes for its therapeutic potential and, as such, endowing the pyrazolo[3,4-d]pyrimidine scaffold with a furoxan moiety may have a significant impact on tumor and metastasis suppression [20,21,22,41].

**Figure 3:**
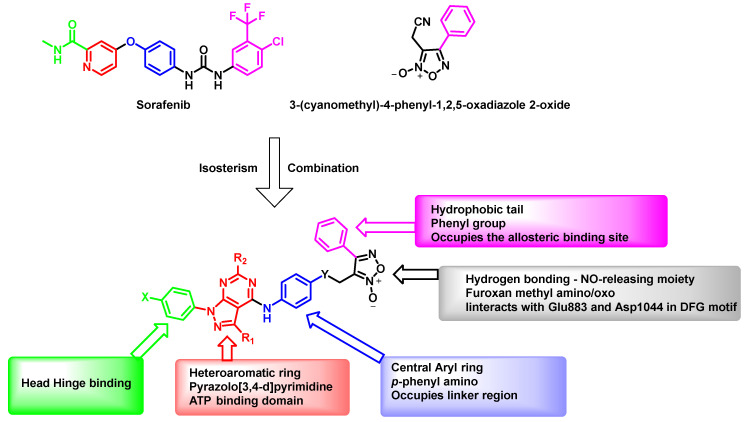
Rational design for target compounds.

Enlightened by these findings, we were inspired to design and synthesize a new series of pyrazolo[3,4-d]pyrimidines tethered to a NO-releasing furoxan moiety through a central aryl bridge. In this context, as shown in Figure 3, our program began with the replacement of the pyridine ring with an isosteric pyrazolo[3,4-d]pyrimidine base-scaffold (in order to fit the ATP-binding domain), the replacement of the oxygen atom with the bioisosteric NH as a hydrogen bond donor (HBD), the keeping of the central aryl moiety without modification as a linker, the replacement of the hydrogen-bonding moiety urea with furoxan methoxy/amino to bind the DFG domain and, finally, the incorporating of the phenyl group as a hydrophobic tail to occupy the allosteric binding region. Furthermore, C3 and C6 functionalizations were studied.

## 2. Results and Discussion

### 2.1. Chemistry

**Scheme 1:**
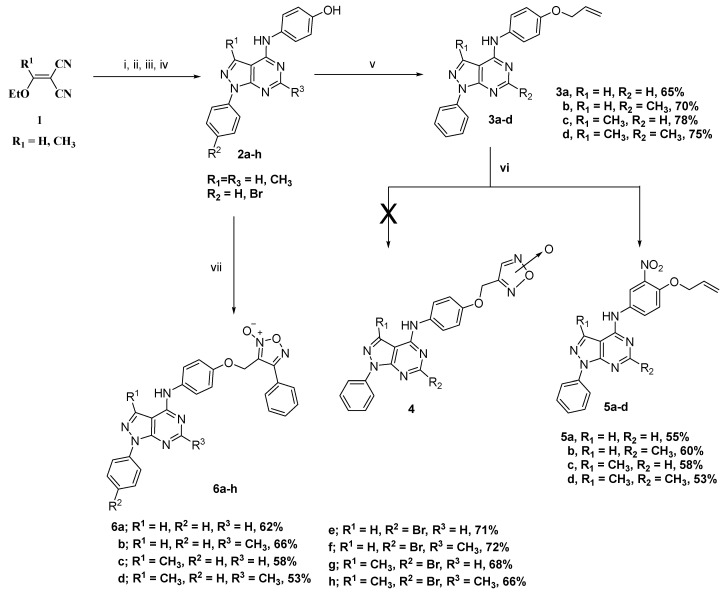
Synthesis of unexpected compounds **5a**–**d** and target compounds **6a**–**h**. (i) Ar-NHNH_2_; (ii) R_2_COOH; (iii) POCl_3_; (iv) *p-*aminophenol; (v) allyl bromide, EtOH, reflux, 45 min.; (vi) NaNO_2_, HOAc, 25–70 °C, 1 h; (vii) 3-(((methylsulfonyl)oxy)methyl)-4-phenyl-1,2,5-oxadiazole 2-oxide, NaOH, THF, reflux, 2 h.

The synthetic process for the target compounds was initiated with a trial to prepare the furoxan moiety from allyl ether precursors **3a**–**d**. As depicted in Scheme 1, the phenolic derivatives **2a**–**h** were prepared from alkylidyne malononitrile, through a multi-step reaction, with reasonable yields [42]. Compounds **2a**–**d** were alkylated, using allyl bromide, to obtain the allyloxy derivatives **3a**–**d** [42,43,44,45]. Upon treating compounds **3a**–**d** with sodium nitrite, an electrophilic aromatic substitution reaction took place at the ortho position to an allyloxy group, wherein the carbons were more shielded, thus providing the nitrated derivatives **5a**–**d,** rather than the desired compound **4** [46]. The unaffected chemical shift of allyl protons through the reaction and disappearance of the doublet at 7.00 ppm corresponding to the protons *ortho* to allyloxy group evidenced the proposed chemical structures. On the other hand, the furoxan moiety was successfully installed through the alkylation of phenolic OH precursors **2a**–**h** using phenyl furoxan methyl mesylate to afford the target compounds **6a**–**h** [21].

**Scheme 2:**
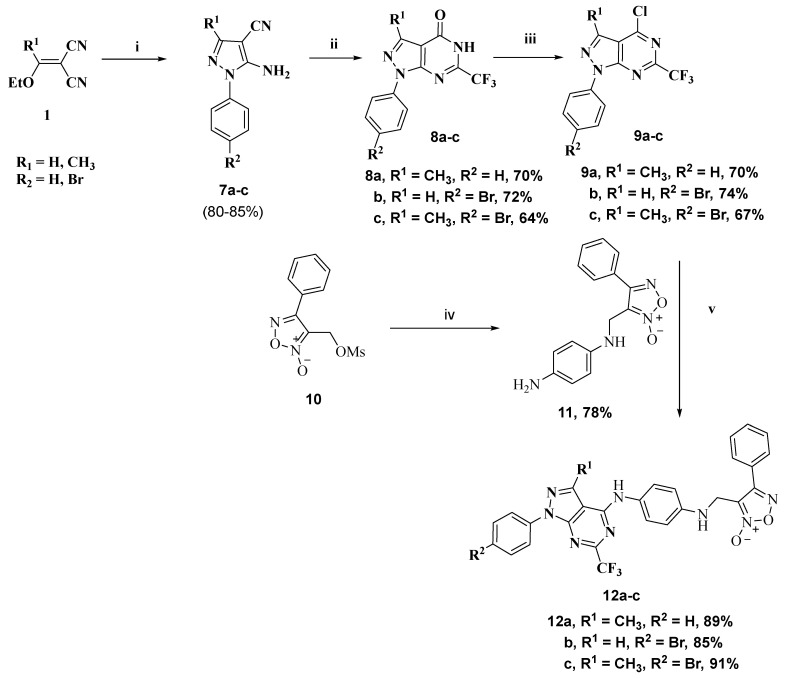
Synthesis of target compounds **12a**–**c**. (i) Ar-NHNH_2_, EtOH, reflux, 15 h; (ii) CF_3_COOH, POCl_3_, reflux, 2 h; (iii) POCl_3_, reflux, 10 h; (iv) *p-*phenylene diamine, TEA, THF, reflux, 2 h; (v) **11**, TEA, NaI, *i-*PrOH, reflux, 2 h.

The fluorinated pyrazolo[3,4-d]pyrimidine derivatives were synthesized as depicted in Scheme 2, in which the bioisosteric furoxan methylamino group was successfully installed on the central aryl ring. The reaction cascade began with the installation of a trifluoromethyl group at C6 of the pyrazolopyrimidine core **8a**–**c**, through the cyclization of the intermediate 5-amino 4-cyanopyrazole derivatives **7a**–**c** with trifluoroacetic acid in the presence of a catalytic amount of phosphorus oxychloride, thus yielding the intermediate 6-trifluoromethyl derivatives **8a**–**c** [47]. The 4-chlorinated derivatives **9a**–**c** were obtained through the refluxing of 6-trifluoromethyl-pyrazolopyrimidinone intermediates **8a**–**c** in phosphorus oxychloride [48]. The NO-releasing source, phenylfuroxan alkarylamine **11**, was obtained with good yield from furoxan mesylate **10**, through refluxing with *p*-phenylenediamine in THF in a ratio of 1:6 [21]. Finally, the target compounds **12a**–**c** were obtained, with reasonable yield, through alkylation of phenylfuroxan alkarylamine **11** with the 4-chloro derivatives **9a**–**c** [42].

**Scheme 3:**
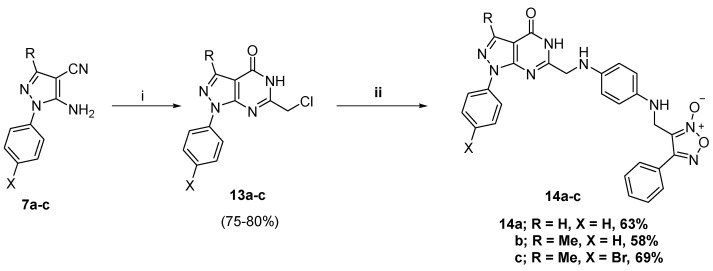
Synthesis of C6-appending compounds **14a**–**c**. (i) ClCH_2_COOH, POCl_3_, THF, reflux, 2 h; (ii) **11**, TEA, NaI, THF, reflux, 2 h.

**Scheme 4:**
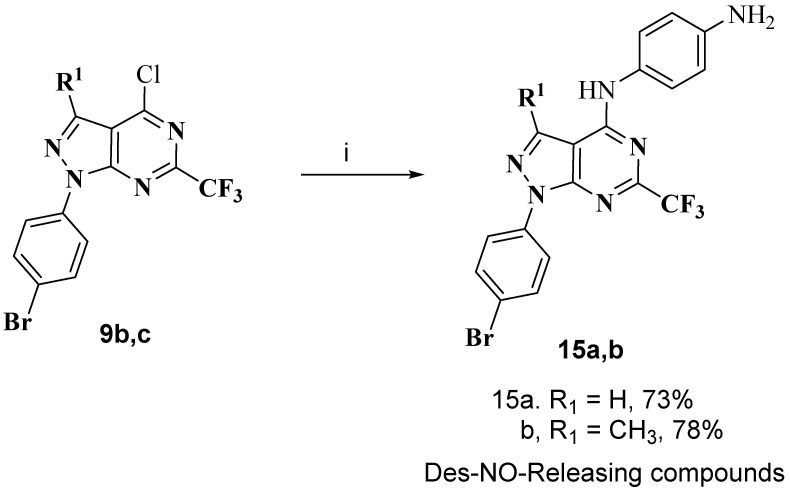
Synthesis of des-NO-releasing compounds **15a** and **b.** (i) *p*-phenylenediamine, NaI, TEA, *i-*PrOH, reflux, 2 h.

Moreover, the C6-substituted derivatives **14a**–**c** were obtained through the series of chemical reactions depicted in Scheme 3. The 6-chloromethyl intermediates **13a**–**c** were synthesized through intermolecular cyclization of the 5-amino-4cyano intermediates **7a**–**c**, using chloroacetic acid and a catalytic amount of phosphorus oxychloride [48,49]. Next, the target compounds **14a**–**c** were prepared by *N*-alkylation of phenylfuroxan alkarylamine **11** with the 6-chlormethyl intermediates **13a**–**c** [50]. Finally, the des-furoxan analogs **15a** and **b** were prepared, according to Scheme 4, through the alkylation of *p*-phenylenediamine with the 6-trifluoromethyl-4-chloro derivatives **9b** and **c** in a 6:1 ratio [42].

### 2.2. Biological Evaluation

#### 2.2.1. In Vitro Anticancer Activity

##### NCI-60 Cell Line Screening

**Figure 4:**
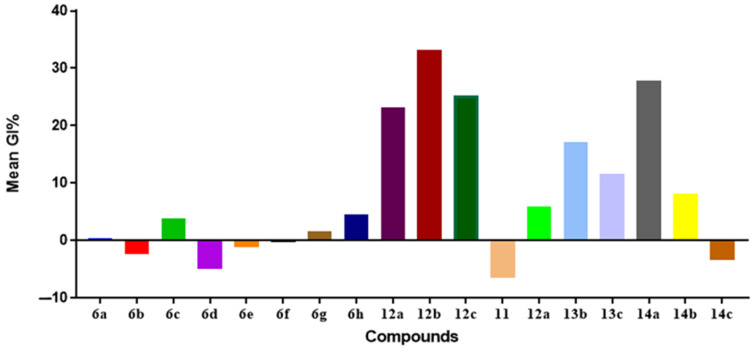
Mean GI% of compounds **6a**–**h, 12a**–**c, 11, 13a**–**c**, and **14a**–**c** against the NCI-59 cell line panel.

The structures of the synthesized compounds **6a**–**h**, **12a**–**c**, **11**, **13a**–**c**, and **14a**–**c** were submitted to the National Cancer Institute’s Developmental Therapeutics Program (NCI-DTP), and all of them were selected for preliminary in vitro anticancer activity screening against NCI-59 human cancer cell lines at a concentration of 10 µM, using the sulforhodamine B (SRB) assay to determine the growth percentage and cell viability [51]. The results for a panel of 59 cancer cell lines demonstrating nine types of cancer (leukemia, non-small cell lung, colon, CNS, melanoma, ovarian, renal, prostate, and breast cancers) are depicted in Tables S1 and S2 (from the Supplementary Material File), and the mean % growth inhibition values (GI%) for the tested compounds against the NCI-59 cancer cell line’s full panel are depicted in Figure 4.

As shown in Figure 4**,** considering the mean growth inhibition % (MGI%) at 10 µM, compounds **6a** and **f** demonstrated no mean growth inhibition percentage, while compounds **6b**, **d**, and **e** enhanced the growth percentage by 1–5%. Compounds **6c**, **g**, and **h** demonstrated low anticancer activity, with GI% = 1.58–4.57%. Additionally, compounds **12a**–**c** exhibited significant inhibitory effects in their anticancer activity, with GI% in the range of 23.2–33.17%.

On the other hand, moving the substitution from C4 (compounds **12a**–**c**) to C6, as in compounds **14a** and **14b**, reduced the growth-inhibitory activity (exhibiting GI% of 27.93 and 8.22, respectively), while compound **14c** enhanced the growth by 3.35%.

Moreover, based on the above findings, compound **12b** was identified as the most active analog among the tested compounds, displaying a better anticancer profile. As shown in Table S1 (from the Supplementary Material File), compound **12b** exhibited growth-inhibitory % activity against the tested panel of up to 77% in leukemia, 64% in non-small cell lung cancer, 62% in colon cancer, 45% in CNS cancer, 72% in melanoma, 40% in ovarian cancer, 69% in renal cancer, 52% in prostate cancer, and 61% in breast cancer cells. Hence, the anticancer profile of compound **12b** against the NCI-59 cancer cell lines encouraged us to further explore its mode of action.

##### Preliminary SARs Study

**Figure 5:**
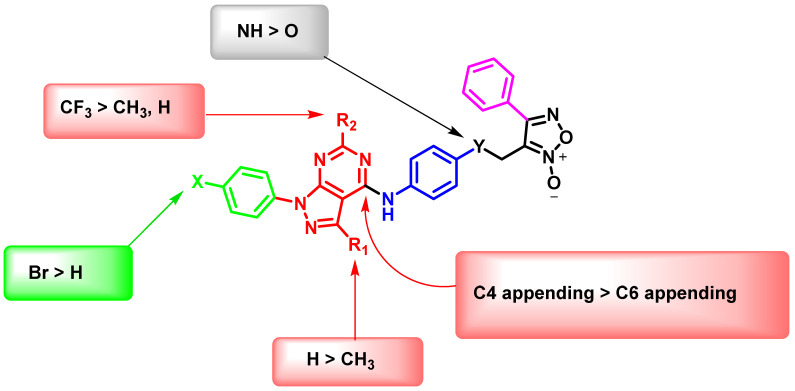
Preliminary SAR of target compounds.

The methyl substitution (EDG) at C3 and C6 (i.e., compounds **6a**–**h**) had no significant effect on the anticancer activity. Notably, the replacement of EDG at C6 in the tested series (i.e., compounds **6a**–**h**) with the electron-withdrawing group CF_3_, and the replacement of the oxygen of the central aryl ring with NH, caused compounds **12a**–**c** to exhibit significant inhibitory effects in their anticancer activity, indicating that the electron-withdrawing substituents at C6 very likely led to enhanced potency. On the other hand, moving the substitution from C4 (compounds **12a**–**c**) to C6, as in compounds **14a** and **14b**, reduced the growth-inhibitory activity while compound **14c** enhanced the growth, indicating that appending the furoxan moiety at C6 and keeping both the pyrazole C3 and 1-phenyl ring unsubstituted may have a good impact on the activity. Furthermore, to test the hybridization hypothesis, compounds **11** and **13a**–**c** were also tested, and the results displayed a significant improvement in anticancer activity, especially for compound **14a**, indicating synergistic efficiency. Consequently, appending the furoxan moiety at C4, utilizing an NH in the central aryl ring at C4, and substituting the C6 with an electron-withdrawing CF_3_ group can be suggested as vital elements for the growth-inhibitory activity of the furoxan-based pyrazolo[3,4-d]pyrimidine hybrids reported herein. A summary, based on the above discussion, of the preliminary structure–activity relationships (SARs) observed in this study is provided in Figure 5.

##### In Vitro Anti-Proliferative Activities

**Figure 6:**
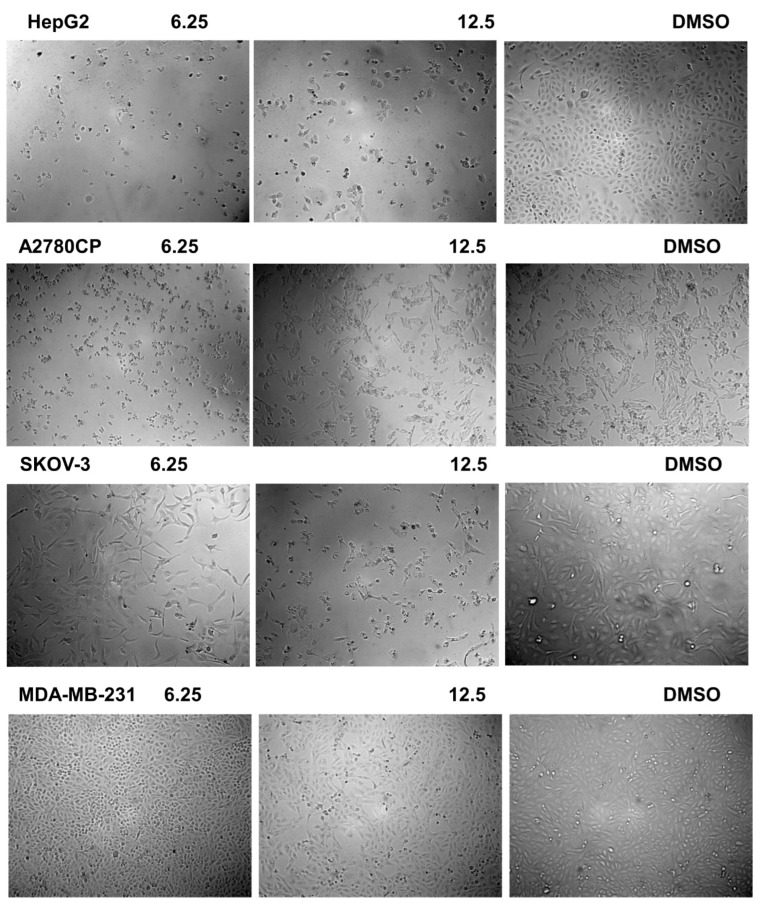
Morphological changes in **12b**-treated cancer cell lines at concentrations of 6.25 and 12.50 µM.

The cell growth-inhibitory activities of the most active compound **12b** were evaluated against human cancer HepG-2 [52,53,54], ovarian [55,56], breast [54], and colon [54] cell lines highly expressing the VEGFR-2 protein. The cells were treated with serial dilutions for 48 h, after which the cell viability was determined by standard MTT assay, using sorafenib as a reference drug. The initial screening of the target compounds showed that **12b** was the most active compound, at a single 50 µM dose, against liver (HepG2), breast (MDA-MB-231 and MCF-7), Colon (HT-29), and ovarian (SKOV-3, A2780, and A2780CP) cancer cell lines. In order to determine the concentration that killed 50% of cells, serial dilutions were used in the treatment, and the determination of percentage of viability identified that **12b** was the most active compound. The IC_50_ values are shown in Table 1 and Figure 6. Compared to sorafenib, the target compound **12b** showed better anticancer activity, with IC_50_ values of 11.5, 11.6, and 13 µM against HepG-2, A2780CP, and MDA-MB-231 cell lines, respectively, while the IC_50_ for SKOV-3 was comparable to that of the reference drug sorafenib. In addition, the morphological changes in **12b**-treated cancer cell lines at concentrations of 6.25 and 12.50 µM are shown in Figure 6. In contrast, the des-NO-releasing compounds **15a** and **b** were tested, but did not inhibit the proliferation of cancer cells at concentrations as high as 50 µM, indicating the significance of the phenylfuroxan moiety in the anticancer activity of **12b**.

Furthermore, the selectivity index (SI) of compound **12b** towards the various cancer cell lines was evaluated by detecting the required concentration to kill 50% of normal human skin fibroblast cells (HSF). The results revealed that compound **12b** did not cause any morphological changes in HSF cells at a concentration as high as 50 µM, indicating its safety and selectivity towards cancer cells, rather than normal cells.

#### 2.2.2. VEGFR Kinase Inhibitory Profile of **12b**

**Figure 7:**
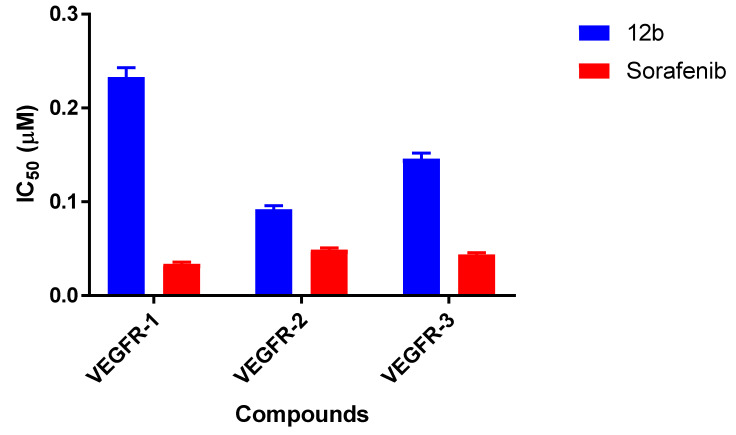
VEGFRx kinase selectivity profiles of **12b** and the reference drug sorafenib.

The fluorinated pyrazolo[3,4-d]pyrimidine derivative based on the 1,2,5-oxadiazole-2-oxide scaffold (**12b**) was evaluated for its VEGFRx (VEGFR-1, VEGFR-2, and VEGFR-3) kinase inhibitory activities, using a VEGFR inhibitory assay with sorafenib as a reference drug. As shown in Figure 7, the results indicated that compound **12b** possesses potency that is comparable to that of sorafenib, displaying noticeable selectivity against VEGFR-2 with an IC_50_ of 0.09 µM.

#### 2.2.3. Flow Cytometric Studies

**Figure 8:**
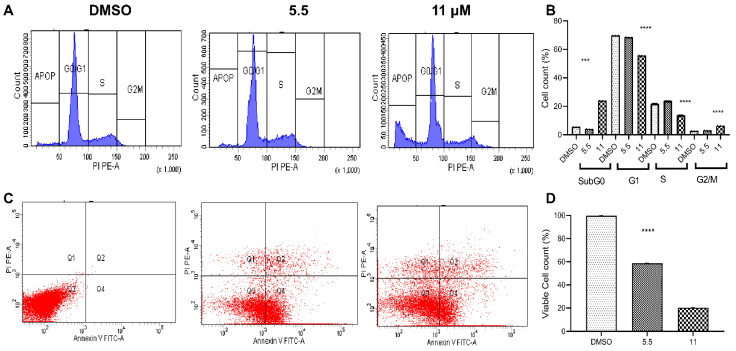
Flow cytometric studies of **12b** in HepG-2 cells: (**A**) Cell-cycle distribution. (**B**) histogram for the change in cell count in each phase in comparison to the control cells. Cells were treated with increasing concentrations for 48 h: control (DMSO), 5.5 µM (1/2 IC_50_), and 11 µM (IC_50_). (**C**) Apoptotic effect of **12b** through Annexin-V-FITC/PI staining assay. Cells were treated with **12b** at the tested concentrations for 24 h. (**D**) Histograms for induction of apoptosis and showing the decrease in viable cell count. **** = significant results in comparison to DMSO-treated cells (*p* < 0.0001). the statistical difference was analyzed by One-Way ANOVA by GraphPad Prism 8.0.

The mechanism of action of **12b** was investigated by evaluating its effectiveness in modulating the cell cycle and induction of apoptosis. As seen in Figure 8A,B, treating HepG2 cells with compound **12b** for 48 h caused cell-cycle arrest at subG1 (24 vs. 5.6 in DMSO-treated cells), leading to the induction of apoptotic pathways (Figure 8C,D).

#### 2.2.4. Intracellular Measurement of NO

**Figure 9:**
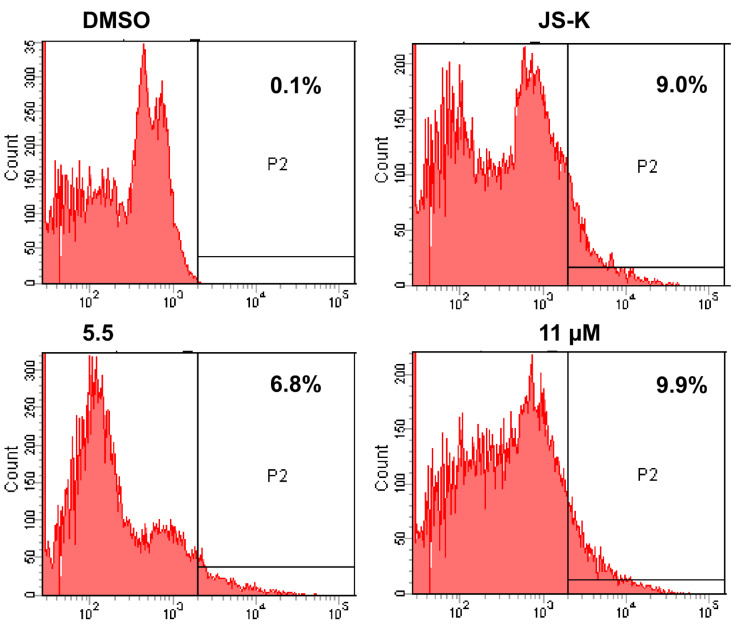
Intracellular measurement of NO for **12b** using DAF-FM DA, with JS-K as a positive control.

The NO-releasing properties of **12b** were evaluated by measuring the level of intracellular NO by flow cytometry, using a cell-permeable and photo-stable NO fluorescent indicator, 3-Amino-4-aminomethyl-2’,7’-difluorofluorescein diacetate (DAF-FM DA), with a detection limit of ~3 nM. DAF-FM diacetate penetrates the cells and is deacetylated by esterase enzyme to yield a non-fluorescent dye, DAF-FM, which, upon reaction with NO, produces a fluorescent benzotriazole product [57]. Compound **12b** was efficiently able to increase the production of NO in the treated cells (9.9% vs. 0.1% in DMSO-treated cells; Figure 9). Furthermore, compound **12b** increased NO production as efficiently as the positive control reference drug JS-k.

#### 2.2.5. Wound-Healing Assay

**Figure 10:**
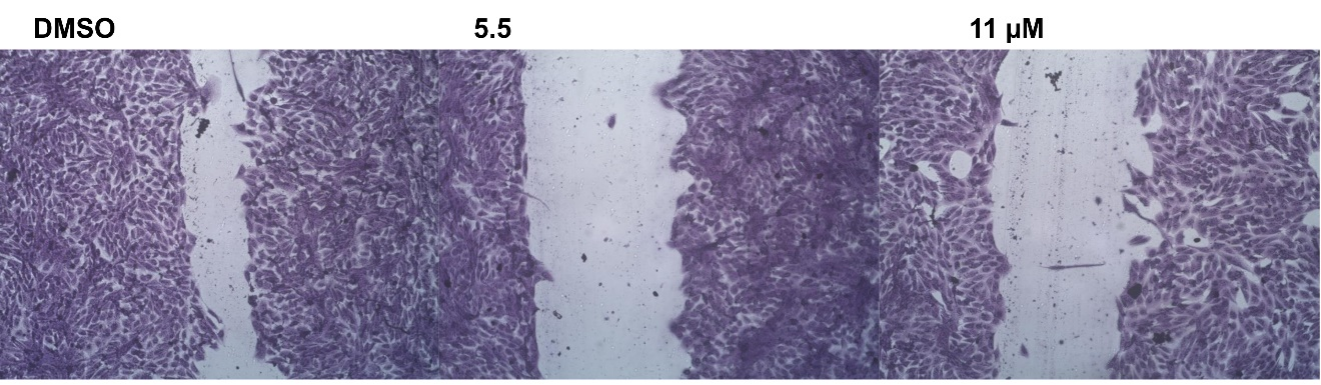
Inhibition of wound healing in **12b**-treated HepG2 cells.

A wound-healing assay is a simple and economical assay used to investigate the anti-metastatic activity of compounds. Cells were treated with compound **12b** in serum-free media for 24 h. After that, the cells were fixed with ice-cold methanol and air-dried, then stained with 0.5% crystal violet. As shown in Figure 10, compound **12b** decreased the rate of wound healing in the absence of serum, in comparison to the DMSO-treated cells. These data revealed that **12b** is an anticancer compound that possesses anti-metastatic activity.

#### 2.2.6. Apoptosis and Metastatic Proteins

**Figure 11:**
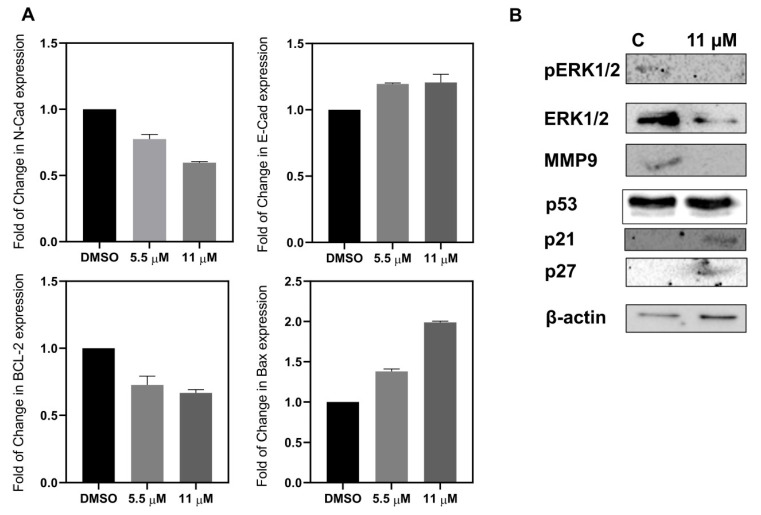
Compound **12b** inhibited cell migration and induced cell-cycle arrest and apoptosis in treated HepG2 cells. (**A**) relative fold of change in metastatic and apoptotic genes. (**B**) western blot analysis for the change in proteins involved in MAPK pathway signaling. Beta-actin was used as a loading control.

Epithelial cells express specific genes involved in maintaining apical–basal polarity and adherence, such as epithelial *E-cadherin*. The epithelial cells migrate to distant locations by over-expressing mesenchymal genes, including neuronal *N-cadherin* and vimentin [58]. Cancer aggressiveness has been positively correlated with the over-expression of *N-cadherin*. Therefore, in this study, the partial mechanism of activity of **12b** was investigated, by quantitative RT-PCR, with respect to *E-cadherin* and *N-cadherin*. The **12b**-treated cells showed lower expression of neuronal *N-cadherin* and increased expression of epithelial *E-cadherin*, indicating the ability of **12b** to suppress metastasis (Figure 11A). Additionally, the ability of **12b** was confirmed by detecting the change in expression of both pro-apoptotic (*Bax*) and anti-apoptotic (*BCL-2*) genes. Compound **12b**-treated HepG2 cells (for 12 h) expressed a low level of *BCL-2* and over-expressed *Bax* (Figure 11A).

**Figure 12:**
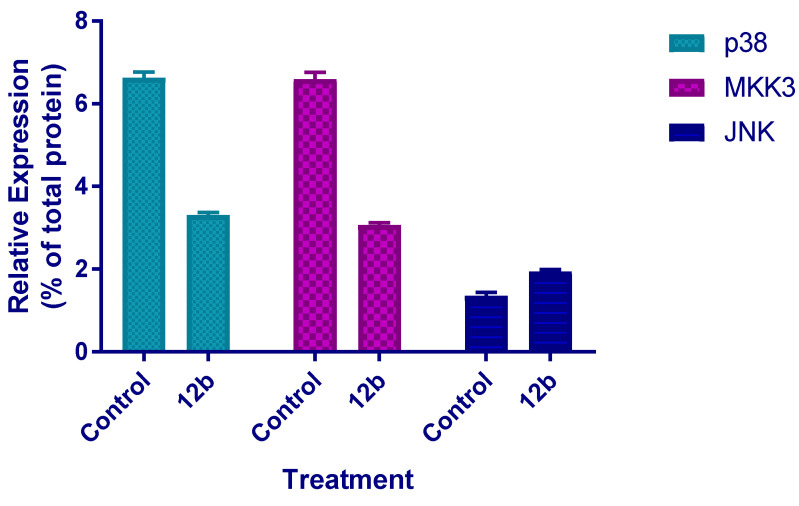
Relative expression of p38, MKK3, and JNK in **12b**-treated HepG-2 cells.

The mitogen-activated protein kinase (MAPK) pathway is considered to be a key player in the gene expression of survival, proliferative, and anti-apoptotic proteins. Aberrant dysregulation in the MAPK pathway activity has been linked to the overall poor prognosis in various cancers [59]. Compound **12b** decreased the level of total ERK and its phosphorylated form (Figure 11B), indicating its ability to modulate signaling pathways. Inhibition of the MAPK pathway led to the downregulation of metalloproteinase MMP-9 (metastatic protein), the over-expression of cell-cycle inhibitors (p21 and p27), and a slight increase in the expression of p53; see Figure 11B. Additionally, compound **12b** was further tested against p38, MKK3, and JNK using ELISA assay protocols [60,61,62] at a concentration of 11 µM. The results indicated that **12b** caused low expression in p38 and MKK3 while JNK was overexpressed; see Figure 12. The overexpression of JNK might have contributed to the NO released from **12b** and reactive oxygen species (ROS). In summary, the novel anticancer lead compound **12b** is worthy of further investigation and activity improvement.

### 2.3. In Silico Molecular Modeling

#### 2.3.1. EON Scaffold Hopping

The discovery of new molecular targets can be achieved by whole-molecule replacements, through the use of physically realistic shapes and electrostatic potential similarities that permit the identification of biologically active molecules with significantly different structures, with respect to existing active agents [63]. OpenEye’s EON application [64] can contribute significantly to lead generation and library design, through the creation of electrostatic Tanimoto (ET) grids using full Poisson–Boltzmann (PB) electrostatics and attaching them to each output molecule [65]. EON is also influenced by the pKa state and formal charges, which have major impacts on the electrostatics, as the query and database molecule are adjusted to a neutral pH model [65]. The data manager in the VIDA module can easily read the EON report file [66]. Sorafenib was included in the library in order to validate the EON results. The designed library was tested against sorafenib as a query reference, and the compounds were ranked according to their shape and electrostatic similarity to sorafenib. As shown in Table 2, the top-scoring compounds were those of the C6-appending series **14a**–**c**, which showed the best alignment, followed by the fluorinated derivatives **12a**–**c** and, then, the rest of the designed compounds.

#### 2.3.2. Docking Studies

**Figure 13:**
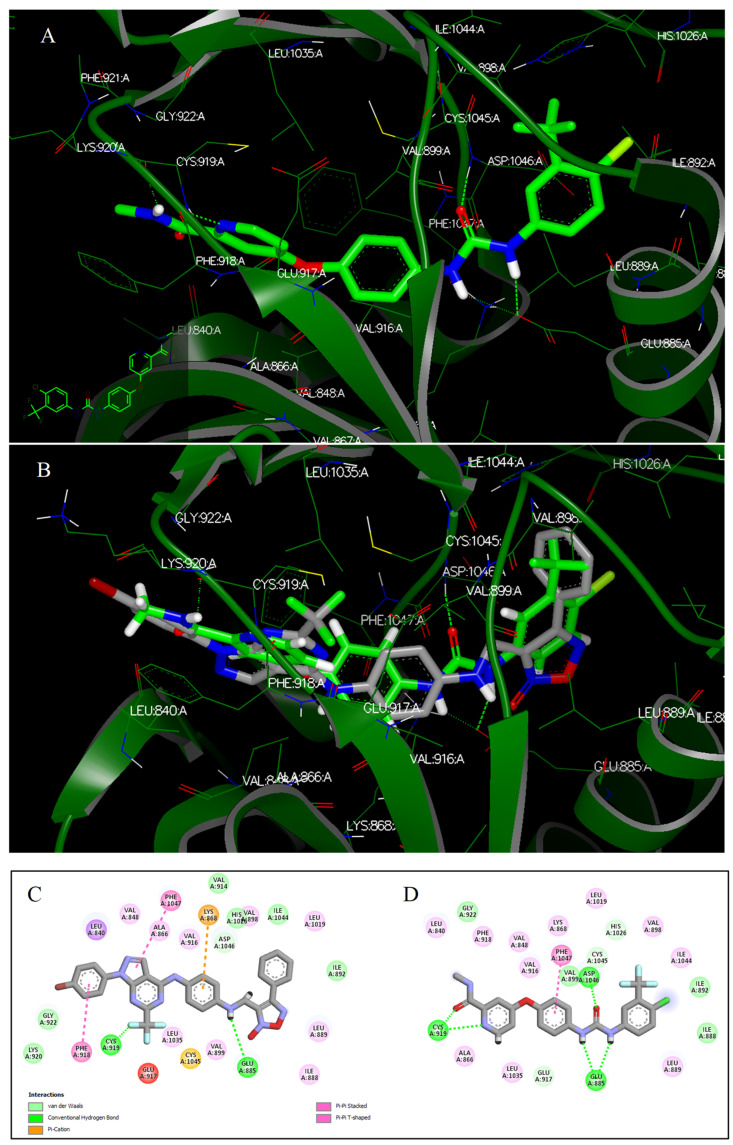
Binding interactions in the active site of VEGFR-2: (**A**) 3D representation of sorafenib; (**B**) 3D representation of **12b** (grey) showing an overlay with sorafenib (green); (**C**) 2D diagram of **12b**; (**D**) 2D diagram of sorafenib.

In silico molecular docking studies were performed using the OpenEye Scientific Software, version 2021.spr, in order to explore the binding modes of the target compounds with the VEGFR-2 active site, adopting the crystal structure of VEGFR-2 in complex with sorafenib (PDB ID: 3WZE). The tested compounds were ranked according to their FRED Chemgauss4 scores—the sum of shape, HB, and desolvation energies—with the lowest score indicating the best binding interactions (see Table 3). To validate the docking results and rationalize the predictive protein–ligand interactions of the target compounds within the active site, the positive drug sorafenib was re-docked using the same procedure as the target compounds. The results demonstrated that the docking pose of the co-crystallized ligand (see Figure 13A), showing interactions similar to those existing in the crystal structure, and the hydrophobic interactions were roughly the same.

The target compounds interacted with the active site in different ways, as follows: (1) The first set, **6a**–**h**, had the same binding pose, in which compounds **6a**, **b**, **d**, and **e** displayed H-bonds between N2 and Cys:919:A (with length ranging from 1.71 to 2.09 Å), as well as between NH and Asp:1046:A (with length ranging from 1.89 to 2.38 Å) in the DFG domain, while compounds **6c**, **f**, **g**, and **h** possessed an additional H-bond, arising from the interaction between furoxan-*N*-oxide and Lys:868:A (length ranging from 2.24 to 2.41 Å); Figure S74A (from the Supplementary Material File). (2) The second set, **14a**–**c**, with C6 appending and pyrimidine amide, showed H-bonds between the carbonyl group at C4 and Cys:919:A (with length ranging from 1.61 to 2.23 Å), as well as H-bonds between NH and Asp:1046:A (bond length 2.04 Å), Glu:885:A (bond length 2.23 Å), and Asp:1046:A (bond length 1.42 Å) in the DFG region, while N5-H and furoxan-2-oxide in compound **14c** displayed two additional hydrogen bonds, with Glu:917:A (bond length 2.27 Å) and Lys:868:A (bond length 2.15 Å); Figure S74B (from the Supplementary Material File). (3) The third set, **12a**–**c**, with trifluoromethyl at C6 and appended C4, displayed different modes of interactions, in which compounds **12a** and **12c** stacked well together (but did not overlie sorafenib), showing two H-bonds coming from furoxan-2-oxide and NH with Lys:919:A (bond lengths 2.18 and 1.95 Å, respectively) and Glu:885:A (bond lengths 2.12 and 1.39 Å, respectively); Figure S74C (from the Supplementary Material File). Overall, the results recommended extensions or modifications for the abovementioned compounds on the central aryl ring, which projects towards the protein cavity.

The most active compound against NCI-60 cell lines, **12b**, was selected as the most plausible binding conformation and, thus, we explored its binding mode within the binding pocket. Compound **12b** displayed a unique binding mode that could underlie its broad-spectrum anticancer activities. As shown in Figure 13B, in spite of not displaying a strong H-bond, **12b** stacked well with sorafenib and tightly fit into the protein pocket without any projection to the protein cavity. The depicted 2D interaction of the protein with **12b** (see Figure 13C,D), generated by Discovery Studio Visualizer [67,68], showed two weak hydrogen bonds, coming from the NH group (HBD) on the furoxan methylamino linker and Glu:885:A (HBA, bond length 4.79Å), as confirmed by the 2D depiction of the protein–ligand interaction using the OpenEye docking report (Figure S75; from the Supplementary Material), as well as between fluorine and Cys:919:A (HBD). Furthermore, there were hydrophobic interactions with the contacted residues. The central aryl ring, pyrazole, and 1-phenyl formed pi–cation electrostatic, pi–pi T-shaped, and pi–pi stacking hydrophobic interactions, respectively. Additionally, **12b** formed Van der Waals and other mixed hydrophobic interactions. The docking report of OpenEye revealed that the furoxan-*N*-oxide formed a hydrogen bond (3%) with Lys:868:A (HBD), as shown in Figure S75 (from the Supplementary Material File). In addition, the FRED docking report showed an additional property relating to the guidance of the optimization of compound **12b,** displaying shape, hydrogen bonding interaction, and protein and ligand desolvation energies, identified through the FRED Chemgauss4 score. Compound **12b** had a FRED chemgauss4 score of −11.37 (where that of sorafenib was −18.58). Thus, either the hydrogen bonds or hydrophobic interactions of **12b** were weaker than those of sorafenib. Simultaneously, as a whole molecule, **12b** spatially stacked well with the co-crystallized ligand and tightly fit into the pocket of VEGFR-2; this provided a reasonable explanation as to why compound **12b** had better anticancer activity among the tested analogs, as confirmed by the NCI-60 cell line screening and its molecular mechanisms.

### 2.4. ADME/Toxicity Analysis

Prediction of the physicochemical, pharmacokinetic, and drug-likeness properties of compound **12b** was achieved using the web-based tool ADMETlab 2.0 [69] which offers a straightforward approach to the comprehensive, accurate, and efficient prediction of ADMET properties using a high-quality database of 250,000 entries covering 53 endpoints and a multi-task graph attention framework [70]. As shown in the radar graph in Figure S76 (from the Supplementary Material File), compound **12b** displayed physicochemical properties comparable to those of sorafenib [69]. In addition, as shown in Table S3 (from the Supplementary Material File), the ADMET analysis revealed that the drug-likeness properties of compound **12b** met the expectations of Pfizer’s rule for designing new drug candidates. The synthetic accessibility score (SAscore), which was designed to estimate the ease-of-synthesis of drug-like molecules, showed a value of 3.406, thus indicating the ease of **12b** synthesis and its good economic cost. Furthermore, it displayed an excellent absorption profile. Notably, its distribution, metabolism, excretion, and toxicity profiles were comparable to those of sorafenib. Furthermore, according to the toxicophoric rules, the acute toxicity during oral administration for **12b** did not show any alerts, in contrast with the reference drug sorafenib. On the other hand, the environmental toxicity rules uncovered that the bioconcentration factors, which are used for considering secondary poisoning potential and assessing risks to human health through the food chain, showed a value of 1.38 − log_10_[(mg/L)/(1000 × MW)], close to that of sorafenib (1.41 − log_10_[(mg/L)/(1000 × MW)]). Furthermore, the environmental toxicity rule for *Tetrahymena pyriformis* 50% growth inhibition concentration (IGC_50_) showed a higher value of 5.086 − log_10_[(mg/L)/(1000 × MW)], compared to that of sorafenib (4.478 − log10[(mg/L)/(1000 × MW)]). Overall, these findings shed light on the ADMET features of **12b**, an intriguing target molecule.

## 3. Materials and Methods

### 3.1. Chemistry

^1^H and ^13^C NMR spectra were obtained using a Bruker AVANCE and Varian Unity INOVA-400 MHz NMR spectrometer in DMSO-*d_6_* using solvent residual peaks as an internal standard; CDCl_3_ (δ = 7.27 ppm) and DMSO-*d_6_* (δ = 2.50 ppm) for ^1^H NMR and CDCl_3_ (δ = 77.23 ppm) and DMSO-*d_6_* (δ = 39.51 ppm) for ^13^C NMR. The signal multiplicities were reported as s (singlet), d (doublet), t (triplet), q (quartet), dd (doublet of doublets), m (multiplet), or br (broad) and the *J*-coupling constants were in Hz. High-resolution mass spectra (HRESI-MS) were acquired on a Thermo Instruments MS system (LTQ XL/LTQ Orbitrap Discovery) linked to a Thermo Instruments HPLC system (Accela PDA detector, Accela PDA autosampler and pump). Thin-layer chromatography (TLC) was performed for reaction monitoring on pre-coated silica gel 60 F_254_ (Merck) sheets and visualized by ultraviolet light (254 nm). Purification of synthesized compounds was achieved via either column chromatography using 150–250 μm silica gel or recrystallization from an appropriate solvent. Solvents and reagents were acquired from commercially available suppliers.

Compounds **2a–h** [42], **7a–c** [42], **8a** [71], **10** [21], and **13a**–**c** [49,72] were prepared as reported.

#### 3.1.1. General Procedure for Preparation of Allyloxy Derivatives (**3a**–**d**)

To a stirred solution of phenolic derivatives **2a**–**d** (0.002 mol) in absolute ethanol (15 mL), allyl bromide (1.209 g, 0.010 mol) and sodium hydroxide (0.080 g, 0.002 mol) were added. After 15 min of reflux, another equivalent of sodium hydroxide was added, and the reflux was continued for another 30 min (TLC monitoring). After cooling, the precipitated solid was filtered, washed with ethanol, dried, and recrystallized from acetonitrile to yield the allyloxy derivatives **3a**–**d** [43,44,45].

##### *N*-(4-(allyloxy)phenyl)-1-phenyl-1*H*-pyrazolo[3,4-d]pyrimidin-4-amine (**3a**)

White powder; 65% yield; ^1^H NMR (400 MHz, DMSO-*d_6_*, *δ* = ppm) *δ* = 10.11 (s, 1H, NH), 8.47 (s, 2H, 3-*H*, 6-*H*), 8.21 (d, *J* = 8.0 Hz, 2H, *H*-Ar), 7.69 (d, *J* = 8.0 Hz, 2H, *H*-Ar), 7.56 (t, 2H, *J* = 8.0 Hz, *H*-Ar), 7.36 (t, *J* = 8.0 Hz, 1H, *H*-Ar), 7.02 (d, *J* = 8.0 Hz, 2H, *H-*Ar), 6.12–6.02 (m, 1H, OCH_2_C*H*=CH_2_), 5.42 (dd, *J* = 17.8, 2.2 Hz, 1H, OCH_2_CH=C*H_2_*), 5.27 (dd, *J* = 10.4, 2.2 Hz, 1H, OCH_2_CH=C*H_2_*), 4.58 (d, *J* = 4.0 Hz, 2H, OC*H_2_*). HRESI-MS m/z calcd for [M+H]^+^: C_20_H_17_N_5_O: 344.1506; found: 344.1497.

##### *N*-(4-(allyloxy)phenyl)-6-methyl-1-phenyl-1*H*-pyrazolo[3,4-d]pyrimidin-4-amine (**3b**)

White powder; 70% yield; ^1^H NMR (400 MHz, DMSO-*d_6_*, *δ* = ppm) *δ* = 9.96 (s, 1H, NH), 8.45 (s, 1H, 3-*H*), 8.20 (d, *J* = 8.0 Hz, 2H, *H*-Ar), 7.71 (s, 2H, *H*-Ar), 7.55 (t, *J* = 8.0 Hz, 2H, *H*-Ar), 7.34 (t, *J* = 8.0 Hz, 1H, *H*-Ar), 7.01 (d, *J* = 8.0 Hz, 2H, *H*-Ar), 6.11–6.02 (m, 1H, OCH_2_C*H*=CH_2_), 5.42 (dd, *J* = 17.3, 1.7 Hz, 1H, OCH_2_CH=C*H_2_*), 5.27 (dd, 1H, *J* = 10.5, 1.6 Hz, OCH_2_CH=C*H_2_*), 4.58 (d, 2H, *J* = 5.3 Hz, OC*H_2_*), 2.53 (s, 3H, C*H_3_*). HRESI-MS m/z calcd for [M+H]^+^: C_21_H_19_N_5_O: 358.1662; found: 368.1655.

##### *N*-(4-(allyloxy)phenyl)-3-methyl-1-phenyl-1*H*-pyrazolo[3,4-d]pyrimidin-4-amine (**3c**)

White powder; 78% yield; ^1^H NMR (400 MHz, DMSO-*d_6_*, *δ* = ppm) *δ* = 8.73 (s, 1H, NH), 8.36 (s, 1H, 6-*H*), 8.18 (d, *J* = 8.0 Hz, 2H**,** *H*-Ar), 7.55–7.50 (m, 4H, *H*-Ar), 7.32 (t, *J* = 8.0 Hz, 1H, *H*-Ar), 6.99 (d, *J* = 8.0 Hz, 2H**,** *H*-Ar), 6.11–6.01 (m, 1H, OCH_2_C*H*=CH_2_), 5.41 (dd, *J* = 17.4, 1.8 Hz, 1H, OCH_2_CH=C*H_2_*), 5.27 (dd, *J* = 10.6, 1.8 Hz, 1H, OCH_2_CH=C*H_2_*), 4.58 (d, *J* = 5.4 Hz, 2H, OC*H_2_*), 2.75 (s, 3H, C*H_3_*). ^13^C NMR (101 MHz, DMSO-*d_6_*) δ 14.81 (*C*H_3_), 68.38 (O*C*H_2_), 101.13 (C3a), 114.48 (*C*-Ar), 117.40 (OCH_2_CH=*C*H_2_), 120.51 (*C*-Ar), 125.57 (*C*-Ar), 125.84 (*C*-Ar), 129.10 (*C*-Ar), 131.37 (*C*-Ar), 133.81 (OCH_2_*C*H=CH_2_), 138.72 (*C*-Ar), 142.41 (*C*-Ar), 154.09 (C3), 155.27 (C7a), 155.85 (C4), 156.02 (C6).

##### *N*-(4-(allyloxy)phenyl)-3,6-dimethyl-1-phenyl-1*H*-pyrazolo[3,4-d]pyrimidin-4-amine (**3d**)

White powder; 75% yield; ^1^H NMR (400 MHz, DMSO-*d_6_*, *δ* = ppm) *δ* = 8.54 (s, 1H, NH), 8.19 (d, *J* = 8.0 Hz, 2H, *H*-Ar), 7.59 (d, *J* = 8.0 Hz, 2H, *H*-Ar), 7.51 (t, *J* = 8.0 Hz, 2H, *H*-Ar), 7.29 (t, *J* = 8.0 Hz, 1H, *H*-Ar), 6.97 (d, *J* = 8.0 Hz, 2H, *H*-Ar), 6.11–6.01 (m, 1H, OCH_2_C*H*=CH_2_), 5.41 (dd, *J* = 17.3, 1.7 Hz, 1H, OCH_2_CH=C*H_2_*), 5.27 (dd, *J* = 10.5, 1.6 Hz, 1H, OCH_2_CH=C*H_2_*), 4.57 (d, *J* = 5.3 Hz, 2H, OC*H_2_*), 2.70 (s, 3H, 3-C*H_3_*), 2.46 (s, 3H, 6-C*H*_3_). ^13^C NMR (101 MHz, DMSO-*d_6_*) δ 14.77 (3-*C*H_3_), 26.21 (6-*C*H_3_), 68.34 (O*C*H_2_), 99.25 (C3a), 114.39 (*C*-Ar), 117.34 (OCH_2_CH=*C*H_2_), 120.39 (*C*-Ar), 124.85 (*C*-Ar), 125.53 (*C*-Ar), 128.98 (*C*-Ar), 131.74 (*C*-Ar), 133.83 (OCH_2_*C*H=CH_2_), 138.93 (*C*-Ar), 142.09 (*C*-Ar), 154.92 (C3), 155.21 (C7a), 155.29 (C4), 165.22 (C6).

#### 3.1.2. General Procedure for Preparation of Nitrated Allyloxy Derivatives (**5a**–**d**)

To a flask charged with allyloxy derivatives **3a**–**d** (0.015 mol) in glacial acetic acid (3 mL), saturated aqueous NaNO_2_ (0.045 mol) solution was added dropwise so that the temperature did not exceed 70 °C [46]. After 1 h, the mixture was diluted with water and extracted with EtOAc. The organic layer was washed with water and brine, dried over anhydrous sodium sulfate, and concentrated in vacuo. The residue was then purified by column chromatography using hexane/ethyl acetate (8:2) as eluent to yield the nitrated derivatives **5a**–**d**.

##### *N*-(4-(allyloxy)-3-nitrophenyl)-1-phenyl-1*H*-pyrazolo[3,4-d]pyrimidin-4-amine (**5a**)

Orange crystals; 55% yield; ^1^H NMR (400 MHz, DMSO-*d_6_*, *δ* = ppm) *δ* = 10.50 (s, 1H, NH), 8.42 (s, 1H, 6-*H*), 8.34 (s, H, 3-*H*), 8.18 (d, *J* = 8.0 Hz, 2H, *H*-Ar), 7.64–7.55 (m, 4H, *H*-Ar), 7.42–7.35 (m, 2H, *H*-Ar), 6.12–6.03 (m, 1H, OCH_2_C*H*=CH_2_), 5.45 (dd, *J* = 17.3, 1.6 Hz, 1H, OCH_2_CH=C*H_2_*), 5.32 (dd, *J* = 10.5, 1.4 Hz, 1H, OCH_2_CH=C*H_2_*), 4.72 (d, *J* = 5.2 Hz, 2H, OC*H_2_*). ^13^C NMR (101 MHz, DMSO-*d_6_*) δ 69.09 (O*C*H_2_), 102.20 (C3a), 110.42 (*C*-Ar), 118.10 (OCH_2_CH=*C*H_2_), 120.85 (*C*-Ar), 120.91 (*C*-Ar), 126.49 (*C*-Ar), 129.22 (*C*-Ar), 129.35 (*C*-Ar), 132.99 (*C*-Ar), 133.59 (OCH_2_*C*H=CH_2_), 138.62 (*C*-Ar), 145.08 (C3), 153.14 (C7a), 155.81 (C4), 155.90 (C6); HRESI-MS m/z calcd for [M+H]^+^: C_20_H_16_N_6_O_3_: 389.1357; found: 389.1347.

##### *N*-(4-(allyloxy)-3-nitrophenyl)-6-methyl-1-phenyl-1*H*-pyrazolo[3,4-d]pyrimidin-4-amine (**5b**)

Orange crystals; 60% yield; ^1^H NMR (400 MHz, DMSO-*d_6_*, *δ* = ppm) *δ* = 10.48 (s, 1H, NH), 8.32 (s, 1H, 3-*H*), 8.18 (d, *J* = 8.0 Hz, 2H, *H*-Ar), 7.58–7.53 (m, 4H, *H*-Ar), 7.40–7.32 (m, 2H, *H*-Ar), 7.34 (t, *J* = 8.0 Hz, 1H, *H*-Ar), 7.01 (d, *J* = 8.0 Hz, 2H, *H*-Ar), 6.11–6.03 (m, 1H, OCH_2_C*H*=CH_2_), 5.45 (dd, *J* = 17.3, 1.5 Hz, 1H, OCH_2_CH=C*H_2_*), 5.32 (dd, *J* = 10.5, 1.4 Hz, 1H, OCH_2_CH=C*H_2_*), 4.71 (d, *J* = 5.3 Hz, 2H, OC*H_2_*), 2.39 (s, 3H, C*H_3_*). ^13^C NMR (101 MHz, DMSO-*d_6_*) δ 25.79 (6-*C*H_3_), 69.07 (O*C*H_2_), 100.31 (C3a), 110.30 (*C*-Ar), 118.06 (OCH_2_CH=*C*H_2_), 120.63 (*C*-Ar), 120.84 (*C*-Ar), 126.26 (*C*-Ar), 128.40 (*C*-Ar), 129.15 (*C*-Ar), 133.04 (*C*-Ar), 133.45 (OCH_2_*C*H=CH_2_), 138.81 (*C*-Ar), 144.88 (*C*-Ar), 153.98 (C3), 154.26 (C7a), 155.53 (C4), 165.08 (C6); HRESI-MS m/z calcd for [M+H]^+^: C_21_H_18_N_6_O_3_: 403.1513; found: 403.1503.

##### *N*-(4-(allyloxy)-3-nitrophenyl)-3-methyl-1-phenyl-1*H*-pyrazolo[3,4-d]pyrimidin-4-amine (**5c**)

Orange crystals; 58% yield; ^1^H NMR (400 MHz, DMSO-*d_6_*, *δ* = ppm) *δ* = 9.59 (s, 1H, NH), 8.40 (s, 1H, 6-*H*), 8.20–8.15 (m, 3H, *H*-Ar), 7.67–7.32 (m, 5H, *H*-Ar), 6.07–6.04 (m, 1H, OCH_2_C*H*=CH_2_), 5.44 (d, *J* = 17.4 Hz, 1H, OCH_2_CH=C*H_2_*), 5.31 (d, *J* = 10.6 Hz, 1H, OCH_2_CH=C*H_2_*), 4.71 (d, *J* = 5.4 Hz, 2H, OC*H_2_*), 2.79 (s, 3H, C*H_3_*). HRESI-MS m/z calcd for [M+H]^+^: C_21_H_18_N_6_O_3_: 403.1513; found: 403.1502.

##### *N*-(4-(allyloxy)-3-nitrophenyl)-3,6-dimethyl-1-phenyl-1*H*-pyrazolo[3,4-d]pyrimidin-4-amine (**5d**)

Orange crystals; 53% yield; ^1^H NMR (400 MHz, DMSO-*d_6_*, *δ* = ppm) *δ* = 9.59 (s, 1H, NH), 8.28–8.14 (m, 3H, *H*-Ar), 7.62–7.28 (m, 5H, *H*-Ar), 6.07–6.02 (m, 1H, OCH_2_C*H*=CH_2_), 5.43 (d, *J* = 17.3 Hz, 1H, OCH_2_CH=C*H_2_*), 5.30 (d, *J* = 10.6 Hz, 1H, OCH_2_CH=C*H_2_*), 4.67 (d, *J* = 4.5 Hz, 2H, OC*H_2_*), 2.72 (s, 3H, 3-C*H_3_*), 2.44 (s, 3H, 6-C*H*_3_). HRESI-MS m/z calcd for [M+H]^+^: C_22_H_20_N_6_O_3_: 417.1670; found: 417.1659.

#### 3.1.3. General Procedure for Preparation of Target Compounds **6a**–**h**

To a flask charged with phenolic derivatives **2a**–**h** (0.002 mol) in THF (15 mL), furoxan mesylate **10** (0.540 g, 0.002 mol) and NaOH (0.080 g, 0.002 mol) were added and the mixture was heated at reflux for 2 h (TLC monitoring) [21]. Then, the mixture was cooled to room temperature, diluted with water, and extracted with EtOAc. The organic layer was washed with brine, dried over anhydrous sodium sulfate, and concentrated in vacuo. The residue was then purified by column chromatography using EtOAc:Hex (1:4) as eluent to provide the target compounds **6a**–**h**.

##### 4-phenyl-3-((4-((1-phenyl-1*H*-pyrazolo[3,4-d]pyrimidin-4-yl)amino)phenoxy)methyl)-1,2,5-oxadiazole 2-oxide (**6a**)

White powder; 62% yield; ^1^H NMR (400 MHz, DMSO-*d_6_*) δ 10.21 (s, 1H, NH), 8.55 (s, 1H, 3-*H*), 8.51 (s, 1H, 6-*H*), 8.22 (d, *J* = 8.0 Hz, 2H, *H*-Ar), 7.88–7.86 (m, 2H, *H*-Ar), 7.76 (d, *J* = 7.6 Hz, 2H, *H*-Ar), 7.69–7.62 (m, 3H, *H*-Ar), 7.57 (t, *J* = 8.0 Hz, 2H, *H*-Ar), 7.38 (t, *J* = 7.4 Hz, 1H, *H*-Ar), 7.08 (d, *J* = 8.0 Hz, 2H, *H*-Ar), 5.25 (s, 2H, OC*H*_2_); ^13^C NMR (101 MHz, DMSO-*d_6_*) δ 157.71 (*C*-Fu), 156.66 (C6), 153.53 (C4), 139.23 (C7a), 134.20 (C3), 133.66 (*C*-Ar), 132.07 (*C*-Ar), 129.97 (*C*-Ar), 129.70 (*C*-Ar), 128.08 (*C*-Ar), 126.84 (*C*-Ar), 126.24 (*C*-Fu), 121.26 (*C*-Ar), 115.84 (*C*-Ar), 113.18 (C3a), 59.38 (O*C*H_2_); DEPT135 ^13^C NMR (101 MHz, DMSO-*d_6_*) δ 156.66, 134.19, 132.07, 129.97, 129.70, 128.08, 126.84, 121.26, 115.84, 59.38 (inverted); Anal. Calcd. for C_26_H_19_N_7_O_3_ (477.48): C, 65.40; H, 4.01; N, 20.53; Found: C, 65.57; H, 4.24; N, 20.49.

##### 3-((4-((6-methyl-1-phenyl-1*H*-pyrazolo[3,4-d]pyrimidin-4-yl)amino)phenoxy)methyl)-4-phenyl-1,2,5-oxadiazole 2-oxide (**6b**)

White powder; 66% yield; ^1^H NMR (400 MHz, DMSO-*d_6_*) δ 10.05 (s, 1H, NH), 8.43 (s, 1H, 3-*H*), 8.22 (d, *J* = 8.0 Hz, 2H, *H*-Ar), 7.91–7.83 (m, 2H, *H*-Ar), 7.79 (s, 2H, *H*-Ar), 7.69–7.60 (m, 3H, *H*-Ar), 7.57 (t, *J* = 7.9 Hz, 2H, *H*-Ar), 7.35 (t, *J* = 7.4 Hz, 1H, *H*-Ar), 7.07 (d, *J* = 8.0 Hz, 2H, H-Ar), 5.24 (s, 2H, OC*H*_2_), 2.55 (s, 3H, C*H*_3_); ^13^C NMR (101 MHz, DMSO-*d_6_*) δ 165.97 (C6), 157.70 (*C*-Fu), 154.67 (C4), 139.43 (C7a), 134.03 (C3), 132.04 (*C*-Ar), 129.95 (*C*-Ar), 129.61 (*C*-Ar), 128.07 (*C*-Ar), 126.59 (*C*-Ar), 126.24 (*C*-Fu), 121.21 (*C*-Ar), 115.83 (*C*-Ar), 113.16 (C3a), 59.37 (O*C*H_2_), 26.81 (6-*C*H_3_); Anal. Calcd. for C_27_H_21_N_7_O_3_ (491.51): C, 65.98; H, 4.31; N, 19.95; Found: C, 65.76; H, 4.59; N, 20.21.

##### 3-((4-((3-methyl-1-phenyl-1*H*-pyrazolo[3,4-d]pyrimidin-4-yl)amino)phenoxy)methyl)-4-phenyl-1,2,5-oxadiazole 2-oxide (**6c**)

White powder; 58% yield; ^1^H NMR (400 MHz, DMSO-*d_6_*) δ 8.95 (s, 1H, NH), 8.39 (s, 1H, 6-*H*), 8.18 (d, *J* = 8.0 Hz, 2H, *H*-Ar), 7.87 (d, *J* = 5.0 Hz, 2H, *H*-Ar), 7.66–7.55 (m, 7H, *H*-Ar), 7.34 (s, 1H, *H-*Ar), 7.07 (d, *J* = 8.0 Hz, 2H, *H*-Ar), 5.26 (s, 2H, OC*H*_2_), 2.77 (s, 3H, C*H*_3_); ^13^C NMR (101 MHz, DMSO-*d_6_*) δ 157.70 (*C*-Fu), 156.13 (C6), 155.99 (C4), 154.44 (C7a), 143.01 (C3), 139.10 (*C*-Ar), 133.07 (*C*-Ar), 132.08 (*C*-Ar), 129.97 (*C*-Ar), 129.61 (*C*-Ar), 128.07 (*C*-Ar), 126.44 (*C*-Ar), 126.24 (*C*-Fu), 126.17 (*C*-Ar), 121.06 (*C*-Ar), 115.51 (*C*-Ar), 113.18 (*C*-Ar), 101.65 (C3a), 59.33 (O*C*H_2_), 15.28 (3-*C*H_3_); Anal. Calcd. for C_27_H_21_N_7_O_3_ (491.51): C, 65.98; H, 4.31; N, 19.95; Found: C, 65.81; H, 4.56; N, 20.14.

##### 3-((4-((3,6-dimethyl-1-phenyl-1*H*-pyrazolo[3,4-d]pyrimidin-4-yl)amino)phenoxy)methyl)-4-phenyl-1,2,5-oxadiazole 2-oxide (**6d**)

White powder; 75% yield; ^1^H NMR (400 MHz, DMSO-*d_6_*) δ 8.61 (s, 1H, NH), 8.20 (d, *J* = 8.0 Hz, 2H, *H*-Ar), 7.87 (d, *J* = 8.0 Hz, 2H, *H*-Ar), 7.67–7.65 (m, 5H, *H*-Ar), 7.53 (t, *J* = 7.6 Hz, 2H, *H*-Ar), 7.31 (t, *J* = 7.1 Hz, 1H, *H*-Ar), 7.05 (d, *J* = 8.0 Hz, 2H, *H*-Ar), 5.25 (s, 2H, OC*H*_2_), 2.72 (s, 3H, C3- C*H*_3_), 2.48 (s, 3H, C6- C*H*_3_); ^13^C NMR (101 MHz, DMSO-*d_6_*) δ 165.72 (C6), 157.70 (*C*-Fu), 155.69 (C4), 155.67 (C7a), 153.97 (*C*-Ar), 142.62 (C3), 139.36 (*C*-Ar), 133.63 (*C*-Ar), 132.06 (*C*-Ar), 129.96 (*C*-Ar), 129.53 (*C*-Ar), 128.07 (*C*-Ar), 126.24 (*C*-Fu), 126.12 (*C*-Ar), 125.29 (*C*-Ar), 120.92 (*C*-Ar), 115.42 (*C*-Ar), 113.18 (*C*-Ar), 99.82 (C3a), 59.32 (O*C*H_2_), 26.70 (6-*C*H_3_), 15.25 (3-*C*H_3_); Anal. Calcd. for C_28_H_23_N_7_O_3_ (505.54): C, 66.52; H, 4.59; N, 19.39; Found: C, 66.34; H, 4.70; N, 19.61.

##### 3-((4-((1-(4-bromophenyl)-1*H*-pyrazolo[3,4-d]pyrimidin-4-yl)amino)phenoxy)methyl)-4-phenyl-1,2,5-oxadiazole 2-oxide (**6e**)

White powder; 71% yield; ^1^H NMR (400 MHz, DMSO-*d_6_*) δ 10.24 (s, 1H, NH), 8.58 (s, 1H, C3-*H*), 8.51 (s, 1H, C6-*H*), 8.24 (d, *J* = 8.0 Hz, 2H, *H*-Ar), 7.87 (d, *J* = 8.0 Hz, 2H, *H*-Ar), 7.78–7.74 (m, 4H, *H*-Ar), 7.67–7.64 (m, 3H, *H*-Ar), 7.08 (d, *J* = 8.8 Hz, 2H, *H*-Ar), 5.25 (s, 2H, OC*H*_2_); Anal. Calcd. for C_26_H_18_BrN_7_O_3_ (556.38): C, 56.13; H, 3.26; N, 17.62; Found: C, 56.42; H, 3.48; N, 17.80.

##### 3-((4-((1-(4-bromophenyl)-6-methyl-1*H*-pyrazolo[3,4-d]pyrimidin-4-yl)amino)phenoxy)methyl)-4-phenyl-1,2,5-oxadiazole 2-oxide (**6f**)

White powder; 72% yield; ^1^H NMR (400 MHz, DMSO-*d_6_*) δ 10.08 (s, 1H, NH), 8.45 (s, 1H, C3-*H*), 8.23 (d, *J* = 8.0 Hz, 2H, *H*-Ar), 7.87 (dd, *J* = 7.6, 1.6 Hz, 2H, *H*-Ar), 7.76 (d, *J* = 8.9 Hz, 4H, *H*-Ar), 7.70–7.59 (m, 3H, *H*-Ar), 7.07 (d, *J* = 8.0 Hz, 2H, *H*-Ar), 5.24 (s, 2H, OC*H*_2_), 2.55 (s, 3H, C6- C*H*_3_); ^13^C NMR (101 MHz, DMSO-*d_6_*) δ 166.19 (C6), 157.70 (*C*-Fu), 154.81 (C4), 138.73 (C7a), 134.50 (C3), 133.89 (*C*-Ar), 132.55 (*C*-Ar), 132.06 (*C*-Ar), 129.97 (*C*-Ar), 128.09 (*C*-Ar), 126.25 (*C*-Fu), 122.73 (*C*-Ar), 118.84 (*C*-Ar), 115.86 (*C*-Ar), 113.18 (C3a), 59.38 (O*C*H_2_), 26.83 (6-*C*H_3_); Anal. Calcd. for C_27_H_20_BrN_7_O_3_ (570.41): C, 56.85; H, 3.53; N, 17.19; Found: C, 56.97; H, 3.72; N, 17.42.

##### 3-((4-((1-(4-bromophenyl)-3-methyl-1*H*-pyrazolo[3,4-d]pyrimidin-4-yl)amino)phenoxy)methyl)-4-phenyl-1,2,5-oxadiazole 2-oxide (**6g**)

White powder; 68% yield; ^1^H NMR (400 MHz, DMSO-*d_6_*) δ 8.86 (s, 1H, NH), 8.40 (s, 1H, C6-*H*), 8.20 (d, *J* = 8.8 Hz, 2H, *H*-Ar), 7.87 (d, *J* = 6.4 Hz, 2H, *H*-Ar), 7.73 (d, *J* = 8.8 Hz, 2H, *H*-Ar), 7.69–7.62 (m, 3H, *H*-Ar), 7.57 (d, *J* = 8.8 Hz, 2H, *H*-Ar), 7.06 (d, *J* = 8.8 Hz, 2H, *H*-Ar), 5.26 (s, 2H, OC*H*_2_), 2.76 (s, 3H, C3- C*H*_3_); ^13^C NMR (101 MHz, DMSO-*d_6_*) δ 157.69 (*C*-Fu), 156.48 (C6), 156.11 (C4), 154.65 (C7a), 154.40 (*C*-Ar), 143.45 (C3), 138.45 (*C*-Ar), 133.09 (*C*-Ar), 132.49 (*C*-Ar), 132.07 (*C*-Ar), 129.96 (*C*-Ar), 128.07 (*C*-Ar), 126.23 (*C*-Ar), 126.14 (*C*-Fu), 122.47 (*C*-Ar), 118.47 (*C*-Ar), 115.47 (*C*-Ar), 113.17 (*C*-Ar), 101.81 (C3a), 59.32 (O*C*H_2_), 15.28 (3-*C*H_3_); Anal. Calcd. for C_27_H_20_BrN_7_O_3_ (570.41): C, 56.85; H, 3.53; N, 17.19; Found: C, 56.99; H, 3.81; N, 17.38.

##### 3-((4-((1-(4-bromophenyl)-3,6-dimethyl-1*H*-pyrazolo[3,4-d]pyrimidin-4-yl)amino)phenoxy)methyl)-4-phenyl-1,2,5-oxadiazole 2-oxide (**6h**)

White powder; 66% yield; ^1^H NMR (400 MHz, DMSO-*d_6_*) δ 8.64 (s, 1H, NH), 8.21 (d, *J* = 8.9 Hz, 2H, *H*-Ar), 7.90–7.84 (m, 2H, *H*-Ar), 7.71 (d, *J* = 8.9 Hz, 2H, *H*-Ar), 7.68–7.59 (m, 5H, *H*-Ar), 7.04 (d, *J* = 8.9 Hz, 2H, *H*-Ar), 5.25 (s, 2H, OC*H*_2_), 2.71 (s, 3H, C3- C*H*_3_), 2.47 (s, 3H, C6- C*H*_3_); ^13^C NMR (101 MHz, DMSO-*d_6_*) δ 165.95 (C6), 157.70 (*C*-Fu), 155.84 (C4), 155.69 (C7a), 154.05 (*C*-Ar), 143.19 (C3), 138.66 (*C*-Ar), 133.51 (*C*-Ar), 132.45 (*C*-Ar), 132.07 (*C*-Ar), 129.97 (*C*-Ar), 128.08 (*C*-Ar), 126.24 (*C*-Fu), 125.39 (*C*-Ar), 122.42 (*C*-Ar), 118.24 (*C*-Ar), 115.44 (*C*-Ar), 113.19 (*C*-Ar), 99.97 (C3a), 59.32 (O*C*H_2_), 26.71 (6-*C*H_3_), 15.25 (3-*C*H_3_); Anal. Calcd. for C_28_H_22_BrN_7_O_3_ (584.43): C, 57.54; H, 3.79; N, 16.78; Found: C, 57.69; H, 3.95; N, 16.97.

#### 3.1.4. General Procedure for Preparation of Pyrazolopyrimidinone Derivatives **8b**,**c**

To a suspension of 5-amino-4-cyanopyrazole intermediates **7b**,**c** (0.002 mol) in TFA (6 mL), phosphorus oxychloride (0.4 mL) was added instantly and the mixture was heated at reflux for 2 h [47]. Then, the reaction mixture was cooled to room temperature and diluted with ice-water. The filtered precipitate was recrystallized from formic acid to afford compounds **8b**,**c** in good yields.

##### 1-(4-bromophenyl)-6-(trifluoromethyl)-1,5-dihydro-4*H*-pyrazolo[3,4-d]pyrimidin-4-one (**8b**)

White powder; 72% yield; ^1^H NMR (400 MHz, DMSO-*d_6_*) δ 13.21 (s, 1H, NH), 8.52 (s, 1H, 3-*H*), 7.99 (d, *J* = 6.4 Hz, 2H, *H*-Ar), 7.83 (d, *J* = 6.4 Hz, 1H, *H*-Ar); ^13^C NMR (101 MHz, DMSO-*d_6_*) δ 158.85 (C4), 150.95 (C6), 137.50 (C7a), 136.89 (C3), 132.83 (*C*-Ar), 123.94 (*C*-Ar), 120.66 (*C*-Ar), 119.82 (*C*-Ar), 117.07 (CF_3_), 108.43 (C3a).

##### 1-(4-bromophenyl)-3-methyl-6-(trifluoromethyl)-1,5-dihydro-4*H*-pyrazolo[3,4-d]pyrimidin-4-one (**8c**)

White powder; 64% yield; ^1^H NMR (400 MHz, DMSO-*d_6_*) δ 13.75 (s, 1H, NH), 7.97 (s, 2H, *H*-Ar), 7.80 (s, 2H, *H*-Ar), 2.57 (s, 3H, C*H*_3_); ^13^C NMR (101 MHz, DMSO-*d_6_*) δ 159.31 (C4), 151.18 (C6), 146.91 (C7a), 137.47 (C3), 132.76 (*C*-Ar), 123.59 (*C*-Ar), 120.18 (*C*-Ar), 119.71 (*C*-Ar), 116.96 (CF_3_), 106.58 (C3a), 13.74 (3-*C*H_3_).

#### 3.1.5. General Procedure for Preparation of 4-Chloro Derivatives **9a**–**c**

A flask charged with suspension of pyrazolopyrimidinone derivatives **8a**–**c** (0.002 mol) in POCl_3_ (16 mL) was heated at reflux for 10 h [48]. Then, the mixture was cooled to room temperature and added dropwise to ice-cooled water. The solid formed was filtered, washed with water, dried, and recrystallized from *n*-hexane to yield the 4-chlorinated derivatives **9a**–**c**.

##### 4-chloro-3-methyl-1-phenyl-6-(trifluoromethyl)-1*H*-pyrazolo[3,4-d]pyrimidine (**9a**)

White powder; 70% yield; ^1^H NMR (400 MHz, DMSO-*d_6_*) δ 8.07 (d, *J* = 7.7 Hz, 2H, *H*-Ar), 7.64 (t, *J* = 7.7 Hz, 2H, *H*-Ar), 7.47 (t, *J* = 7.1 Hz, 1H, *H*-Ar), 2.79 (s, 3H, C*H*_3_). ^13^C NMR (101 MHz, DMSO-*d_6_*) δ 156.22 (C6), 153.22 (C4), 144.50 (C7a), 137.70 (C3), 130.08 (*C*-Ar), 129.85 (*C*-Ar), 128.16 (*C*-Ar), 122.10 (*C*-Ar), 121.96 (CF_3_), 115.14 (C3a), 14.33 (3-*C*H_3_).

##### 1-(4-bromophenyl)-4-chloro-6-(trifluoromethyl)-1*H*-pyrazolo[3,4-d]pyrimidine (**9b**)

White powder; 74% yield; ^1^H NMR (400 MHz, DMSO-*d_6_*) δ 9.04 (s, 1H, 3-*H*), 8.09 (d, *J* = 8.9 Hz, 2H, *H*-Ar), 7.90 (d, *J* = 8.9 Hz, 2H, *H*-Ar).

##### 1-(4-bromophenyl)-4-chloro-3-methyl-6-(trifluoromethyl)-1*H*-pyrazolo[3,4-d]pyrimidine (**9c**)

White powder; 67% yield; ^1^H NMR (400 MHz, DMSO-*d_6_*) δ 8.03 (d, *J* = 8.8 Hz, 2H, *H*-Ar), 7.83 (d, *J* = 8.4 Hz, 2H, *H*-Ar), 2.78 (s, 3H, C*H*_3_).

#### 3.1.6. General Procedure for Preparation of Phenylfuroxan Derivative **11**

To a flask charged with a solution of furoxan mesylate **10** (0.270 g, 0.001 mol) in THF (10 mL), *p*-phenylendiamine (0.642 g, 0.006 mol) and TEA (0.001 mol) were added, and the reaction mixture was heated at reflux for 2 h (TLC monitoring) [21]. Then, the mixture was cooled to room temperature and extracted with EtOAc, then washed with hot water 3 times or until the disappearance of unreacted *p*-phenylenediamine. The organic layer was dried over anhydrous Na_2_SO_4_ and concentrated in vacuo. The residue was then purified by column chromatography using EtOAc:Hex (1:1) to give the phenylfuroxan derivative **11** in good yield.

##### 3-(((4-aminophenyl)amino)methyl)-4-phenyl-1,2,5-oxadiazole 2-oxide (**11**)

White powder; 78% yield; ^1^H NMR (400 MHz, DMSO-*d_6_*) δ 7.86 (dd, *J* = 7.9, 1.4 Hz, 2H, *H*-Ar), 7.68–7.54 (m, 3H, *H*-Ar), 6.42 (d, *J* = 8.6 Hz, 2H, *H*-Ar), 6.34 (d, *J* = 8.6 Hz, 2H, *H*-Ar), 5.40 (t, *J* = 5.8 Hz, 1H, NH, exchangeable), 4.42 (s, 2H, NH_2_, exchangeable), 4.25 (d, *J* = 5.8 Hz, 2H, OC*H*_2_); ^13^C NMR (101 MHz, DMSO-*d_6_*) δ 157.83 (*C*-Fu), 140.82 (*C*-Ar), 138.66 (*C*-Ar), 131.70 (*C*-Ar), 129.73 (*C*-Ar), 128.19 (*C*-Ar), 126.70 (*C*-Fu), 115.80 (*C*-Ar), 114.95 (*C*-Ar), 114.61 (*C*-Ar), 38.42 (NH*C*H_2_).

#### 3.1.7. General Procedure for Preparation of Fluorinated Target Compounds **12a**–**c**

To a stirred solution of 6-trifluoromethyl-4-chloro derivatives **9a**–**c** (0.001 mol) in isopropanol (10 mL), 3-(((4-aminophenyl)amino)methyl)-4-phenyl-1,2,5-oxadiazole 2-oxide **11** (0.282 g, 0.001 mol), TEA (0.001 mol), and NaI (0.0005 mol) were added, and the mixture was heated under reflux for 2 h (TLC monitoring) [42]. Then, the reaction mixture was cooled to room temperature and diluted with water. The isolated solid was recrystallized form absolute ethanol to give the target compounds **12a**–**c** in good yields.

##### 3-(((4-((3-methyl-1-phenyl-6-(trifluoromethyl)-1*H*-pyrazolo[3,4-d]pyrimidin-4-yl)amino)phenyl)amino)methyl)-4-phenyl-1,2,5-oxadiazole 2-oxide (**12a**)

White powder; 89% yield; ^1^H NMR (400 MHz, DMSO-*d_6_*) δ 9.12 (s, 1H, NH), 8.11 (d, *J* = 7.1 Hz, 2H, *H*-Ar), 7.85 (d, *J* = 5.7 Hz, 2H, *H*-Ar), 7.60 (m, 5H, *H*-Ar), 7.36 (d, *J* = 7.2 Hz, 3H, *H*-Ar), 6.59 (d, *J* = 7.6 Hz, 2H, *H*-Ar), 6.38 (s, 1H, N*H*CH_2_), 4.41 (s, 2H, NHC*H*_2_), 2.72 (s, 3H, C3- C*H*_3_); ^13^C NMR (101 MHz, DMSO-*d_6_*) δ 157.81 (*C*-Fu), 156.56 (C6), 153.83 (C4), 153.19 (C7a), 152.84 (C3), 145.57 (*C*-Ar), 143.39 (*C*-Ar), 138.66 (*C*-Ar), 131.79 (*C*-Ar), 129.80 (*C*-Ar), 129.75 (*C*-Ar), 128.80 (*C*-Ar), 128.19 (*C*-Ar), 126.99 (*C*-Ar), 126.56 (*C*-Fu), 126.01 (*C*-Ar), 121.39 (*C*-Ar), 114.57 (CF_3_), 112.48 (*C*-Ar), 101.80 (C3a), 37.27 (NH*C*H_2_), 15.20 (3-*C*H_3_); DEPT135 ^13^C NMR (101 MHz, DMSO-*d_6_*) δ 131.79, 129.80, 129.75, 128.19, 126.99, 126.01, 121.38, 112.48, 37.27 (inverted), 15.20; Anal. Calcd. for C_28_H_21_F_3_N_8_O_2_ (558.53): C, 60.21; H, 3.79; N, 20.06; Found: C, 59.97; H, 3.88; N, 19.89.

##### 3-(((4-((1-(4-bromophenyl)-6-(trifluoromethyl)-1*H*-pyrazolo[3,4-d]pyrimidin-4-yl)amino)phenyl)amino)methyl)-4-phenyl-1,2,5-oxadiazole 2-oxide (**12b**)

White powder; 85% yield; ^1^H NMR (400 MHz, DMSO-*d_6_*) δ 10.56 (s, 1H, NH), 8.68 (s, 1H, C3-*H*), 8.13 (d, *J* = 8.3 Hz, 1H, *H*-Ar), 8.10–7.99 (m, 1H, *H*-Ar), 7.83 (dd, *J* = 14.4, 7.5 Hz, 4H, *H*-Ar), 7.68–7.57 (m, 3H, *H*-Ar), 7.53 (d, *J* = 8.2 Hz, 1H, *H*-Ar), 7.16 (m, 1H, *H*-Ar), 6.68–6.54 (m, 2H, *H*-Ar), 6.36 (s, 1H, N*H*CH_2_), 4.44 (s, 2H, NHC*H*_2_); Anal. Calcd. for C_27_H_18_BrF_3_N_8_O_2_ (623.39): C, 52.02; H, 2.91; N, 17.98; Found: C, 52.24; H, 3.14; N, 18.15.

##### 3-(((4-((1-(4-bromophenyl)-3-methyl-6-(trifluoromethyl)-1*H*-pyrazolo[3,4-d]pyrimidin-4-yl)amino)phenyl)amino)methyl)-4-phenyl-1,2,5-oxadiazole 2-oxide (**12c**)

White powder; 91% yield; ^1^H NMR (400 MHz, DMSO-*d_6_*) δ 9.13 (s, 1H, NH), 8.08 (d, *J* = 8.1 Hz, 2H, *H*-Ar), 7.85 (d, *J* = 6.1 Hz, 2H, *H*-Ar), 7.74 (d, *J* = 8.1 Hz, 2H, *H*-Ar), 7.62 (d, *J* = 7.1 Hz, 3H, *H*-Ar), 7.34 (d, *J* = 7.8 Hz, 2H, *H*-Ar), 6.59 (d, *J* = 7.8 Hz, 2H, *H*-Ar), 6.38 (s, 1H, N*H*CH_2_), 4.40 (d, *J* = 3.4 Hz, 2H, NHC*H*_2_), 2.70 (s, 3H, C3-C*H*_3_); ^13^C NMR (101 MHz, DMSO-*d_6_*) δ 157.79 (*C*-Fu), 156.48 (C6), 153.93 (C4), 153.25 (C7a), 152.90 (C3), 145.60 (*C*-Ar), 143.79 (*C*-Ar), 137.94 (*C*-Ar), 132.60 (*C*-Ar), 131.77 (*C*-Ar), 129.79 (*C*-Ar), 128.17 (*C*-Ar), 127.91 (*C*-Ar), 126.55 (*C*-Fu), 126.02 (*C*-Ar), 122.76 (*C*-Ar), 121.52 (*C*-Ar), 119.18 (*C*-Ar), 118.78 (*C*-Ar), 114.54 (CF_3_), 112.46 (*C*-Ar), 101.94 (C3a), 37.26 (NH*C*H_2_), 15.20 (3-*C*H_3_); Anal. Calcd. for C_28_H_20_BrF_3_N_8_O_2_ (637.42): C, 52.76; H, 3.16; N, 17.58; Found: C, 52.93; H, 3.42; N, 17.81.

#### 3.1.8. General Procedure for Preparation of C6 Appending Target Compounds **14a**–**c**

To a stirred solution of 6-chloromethyl derivatives **13a**–**c** (0.001 mol) in THF (10 mL), phenylfuroxan derivative **11** (0.282 g, 0.001 mol), TEA (0.001 mol), and NaI (0.0005 mol) were added, and the reaction mixture was heated under reflux for 2 h (TLC monitoring) [50]. Then, the mixture was cooled to room temperature, diluted with water, and extracted with EtOAc. The organic layer was dried over anhydrous Na_2_SO_4_ then concentrated in vacuo to afford the crude product, which was purified by column chromatography using EtOAc:Hex (3:7) as eluent to yield the C6 appended target compounds **14a**–**c**.

##### 3-(((4-(((4-oxo-1-phenyl-4,5-dihydro-1**H**-pyrazolo[3,4-d]pyrimidin-6-yl)methyl)amino)phenyl)amino)methyl)-4-phenyl-1,2,5-oxadiazole 2-oxide (**14a**)

Yellow powder; 63% yield; ^1^H NMR (400 MHz, DMSO-*d_6_*) δ 12.20 (s, 1H, CON*H*, D_2_O exchangeable), 8.29 (s, 1H, C3-*H*), 7.98 (d, *J* = 7.8 Hz, 2H, *H*-Ar), 7.81 (d, *J* = 7.1 Hz, 2H, *H*-Ar), 7.57–7.47 (m, 5H, *H*-Ar), 7.36 (t, *J* = 7.4 Hz, 1H, *H*-Ar), 6.54 (d, *J* = 8.7 Hz, 2H, *H*-Ar), 6.42 (d, *J* = 8.7 Hz, 2H, *H*-Ar), 5.55–5.53 (two s, 2H, two NH, D_2_O exchangeable), 4.26–4.25 (two s, 4H, two C*H_2_*); ^13^C NMR (101 MHz, DMSO-*d_6_*) δ 160.99 (C4), 158.20 (C6), 157.76 (*C*-Fu), 152.49 (C7a), 140.56 (C3), 139.54 (*C*-Ar), 138.78 (*C*-Ar), 136.41 (*C*-Ar), 131.65 (*C*-Ar), 129.66 (*C*-Ar), 129.62 (*C*-Ar), 128.14 (*C*-Ar), 127.28 (*C*-Ar), 126.66 (*C*-Fu), 121.81 (*C*-Ar), 114.87 (*C*-Ar), 114.74 (*C*-Ar), 114.33 (*C*-Ar), 106.58 (C3a), 46.89 (6-*C*H_2_), 38.18 (NH*C*H_2_).

##### 3-(((4-(((3-methyl-4-oxo-1-phenyl-4,5-dihydro-1*H*-pyrazolo[3,4-d]pyrimidin-6-yl)methyl)amino)phenyl)amino)methyl)-4-phenyl-1,2,5-oxadiazole 2-oxide (**14b**)

Yellow powder; 58% yield; ^1^H NMR (400 MHz, DMSO-*d_6_*) δ 12.11 (s, 1H, CON*H*, D_2_O exchangeable), 7.97 (d, *J* = 7.7 Hz, 2H, *H*-Ar), 7.81 (d, *J* = 7.0 Hz, 2H, *H*-Ar), 7.55–7.42 (m, 5H, *H*-Ar), 7.31 (t, *J* = 6.9 Hz, 1H, *H*-Ar), 6.55 (d, *J* = 8.0 Hz, 2H, *H*-Ar), 6.44 (d, *J* = 8.0 Hz, 2H, *H*-Ar), 5.56–5.52 (two s, 2H, two NH, D_2_O exchangeable), 4.24 (s, 4H, two C*H_2_*), 2.51 (s, 3H, C*H_3_*); ^13^C NMR (101 MHz, DMSO-*d_6_*) δ 160.99 (C4), 158.95 (C6), 157.72 (*C*-Fu), 152.87 (C7a), 146.31 (C3), 140.53 (*C*-Ar), 139.56 (*C*-Ar), 138.80 (*C*-Ar), 131.62 (*C*-Ar), 129.64 (*C*-Ar), 129.63 (*C*-Ar), 129.49 (*C*-Ar), 128.11 (*C*-Ar), 126.76 (*C*-Fu), 126.63 (*C*-Ar), 121.46 (*C*-Ar), 114.82 (*C*-Ar), 114.77 (*C*-Ar), 114.35 (*C*-Ar), 104.68 (C3a), 46.93 (6-*C*H_2_), 38.17 (NH*C*H_2_), 13.78 (3-*C*H_3_).

##### 3-(((4-(((1-(4-bromophenyl)-3-methyl-4-oxo-4,5-dihydro-1*H*-pyrazolo[3,4-d]pyrimidin-6-yl)methyl)amino)phenyl)amino)methyl)-4-phenyl-1,2,5-oxadiazole 2-oxide (**14c**)

Yellow powder; 69% yield; ^1^H NMR (400 MHz, DMSO-*d_6_*) δ 12.17 (s, 1H, CON*H*, D_2_O exchangeable), 7.96 (d, *J* = 8.8 Hz, 2H, *H*-Ar), 7.79 (d, *J* = 7.2 Hz, 2H, *H*-Ar), 7.64 (d, *J* = 8.8 Hz, 2H, *H*-Ar), 7.57–7.49 (m, 3H, *H*-Ar), 6.54 (d, *J* = 8.6 Hz, 2H, *H*-Ar), 6.43 (d, *J* = 8.6 Hz, 2H, *H*-Ar), 5.56 (t, *J* = 5.7 Hz, 1H, NH, D_2_O exchangeable), 5.51 (s, 1H, NH, D_2_O exchangeable), 4.24 (d, *J* = 5.9 Hz, 4H, two C*H_2_*), 2.49 (s, 3H, C*H_3_*); ^13^C NMR (101 MHz, DMSO-*d_6_*) δ 161.35 (C4), 158.82 (C6), 157.73 (*C*-Fu), 153.04 (C7a), 146.73 (C3), 140.51 (*C*-Ar), 139.57 (*C*-Ar), 138.06 (*C*-Ar), 132.40 (*C*-Ar), 131.63 (*C*-Ar), 129.64 (*C*-Ar), 128.12 (*C*-Ar), 126.63 (*C*-Fu), 123.05 (*C*-Ar), 119.15 (*C*-Ar), 114.82 (*C*-Ar), 114.77 (*C*-Ar), 114.36 (*C*-Ar), 104.89 (C3a), 46.84 (6-*C*H_2_), 38.19 (NH*C*H_2_), 13.78 (3-*C*H_3_).

#### 3.1.9. General Procedure for Preparation of Des-NO-Releasing Compounds **15a**,**b**

To a stirred solution of 6-trifluoromethyl-4-chloro derivatives **9b**,**c** (0.001 mol) in isopropanol (10 mL), *p*-phenylenediamine (0.108 g, 0.001 mol), TEA (0.001 mol), and NaI (0.0005 mol) were added and the reaction mixture was heated under reflux for 2 h (TLC monitoring) [42]. Then, the mixture was cooled to room temperature and diluted with water. The formed precipitate was filtered and recrystallized form absolute ethanol to give the des-furoxan derivatives **15a**,**b** in good yields.

##### *N*1-(1-(4-bromophenyl)-6-(trifluoromethyl)-1*H*-pyrazolo[3,4-d]pyrimidin-4-yl)benzene-1,4-diamine (**15a**)

White powder; 73% yield; ^1^H NMR (400 MHz, DMSO-*d_6_*) δ 10.46 (d, *J* = 6.6 Hz, 1H, NH), 8.64 (s, 1H, C3-*H*), 8.11 (d, *J* = 8.6 Hz, 1H, *H*-Ar), 8.02 (d, *J* = 8.5 Hz, 1H, *H*-Ar), 7.75 (dd, *J* = 12.8, 8.3 Hz, 2H, *H*-Ar), 7.43 (d, *J* = 8.4 Hz, 1H, *H*-Ar), 7.08 (d, *J* = 8.1 Hz, 1H, *H*-Ar), 6.77–6.61 (m, 2H, *H*-Ar), 5.47 (s, 1H, NH_2_), 5.14 (s, 1H, NH_2_); ^13^C NMR (101 MHz, DMSO-*d_6_*) δ 159.48 (C6), 154.83 (C4), 153.26 (C7a), 146.55 (C3), 138.07 (*C*-Ar), 135.17 (*C*-Ar), 134.94 (*C*-Ar), 132.72 (*C*-Ar), 128.65 (*C*-Ar), 123.59 (*C*-Ar), 123.13 (*C*-Ar), 114.89 (CF_3_), 114.25 (*C*-Ar), 103.21 (C3a).

##### *N*1-(1-(4-bromophenyl)-3-methyl-6-(trifluoromethyl)-1*H*-pyrazolo[3,4-d]pyrimidin-4-yl)benzene-1,4-diamine (**15b**)

White powder; 78% yield; ^1^H NMR (400 MHz, DMSO-*d_6_*) δ 9.39–8.97 (s, 1H, NH), 8.09 (d, *J* = 8.6 Hz, 2H, *H*-Ar), 7.75 (d, *J* = 8.6 Hz, 2H, *H*-Ar), 7.24 (d, *J* = 8.2 Hz, 2H, *H*-Ar), 6.62 (d, *J* = 8.2 Hz, 2H, *H*-Ar), 5.18 (s, 2H, NH_2_), 2.93–2.54 (s, 3H, C*H_3_*); ^13^C NMR (101 MHz, DMSO-*d_6_*) δ 154.02 (C6), 153.34 (C4), 153.30 (C7a), 147.27 (C3), 143.88 (*C*-Ar), 137.97 (*C*-Ar), 132.65 (*C*-Ar), 126.44 (*C*-Ar), 126.20 (*C*-Ar), 122.89 (*C*-Ar), 121.54 (*C*-Ar), 119.21 (*C*-Ar), 114.02 (CF_3_), 101.84 (C3a), 15.22 (3-*C*H_3_).

### 3.2. Biological Evaluation

#### 3.2.1. NCI-60 Cell Line Screening

The in vitro NCI-60 cell line screening assay was conducted at the National Cancer Institute (NCI), Bethesda, USA against 59 cancer cell lines representing 9 types of cancers, as reported earlier [51,73]. Briefly, the human cancer cell lines were grown in RPMI 1640 medium containing 5% fetal bovine serum and 2 mM L-glutamine and inoculated into 96-well microtiter plates in 100 μL (5000 to 40,000 cells/well). Then, the plates were incubated at 37 °C, 5% CO_2_, 95% air, and 100% relative humidity for 24 h. After that, two plates of each cell line were fixed in situ with TCA, to represent cell population at time zero (Tz). The tested compounds were dissolved in DMSO at 400-fold and added so that the final concentration became 10 μM. The plates were then incubated for an additional 48 h followed by the addition of cold TCA to terminate the assay in case of adherent cells. Then, the cells were fixed in situ by cold 50% (*w*/*v*) TCA (50 μL; final concentration, 10% TCA) and incubated for 1 h at 4 °C. After discarding the supernatant, the plates were washed 5 times with tap water and air dried. Sulforhodamine B (SRB) solution (100 μL) at 0.4% (*w*/*v*) in 1% HOAc was added to each well, and plates were incubated for 10 min. at rt. Then, washing with 1% acetic acid was conducted 5 times to remove the unbound dye followed by air drying. After that, 10 mM trizma base was added to solubilize the bound stain, and the absorbance was read on an automated plate reader at a wavelength of 515 nm. On the other hand, the methodology for suspension cells was the same except that 50 μL of 80% TCA (final concentration, 16% TCA) was gently added to terminate the assay by fixing settled cells at the bottom of the wells. Percentage growth inhibition (GI%) was calculated as follows: 

$\mathrm{Growth}\;\mathrm{percentage}=\frac{\mathrm{Ti}\;\left(\mathrm{test}\;\mathrm{growth}\;\mathrm{in}\;\mathrm{the}\;\mathrm{presence}\;\mathrm{of}\;\mathrm{compound}\;\mathrm{at}\;10\;µM\;\right)-\mathrm{Tz}\;\left(\mathrm{time}\;\mathrm{zero}\right)}{C\;\left(\mathrm{control}\;\mathrm{growth}\right)-\mathrm{Tz}\;\left(\mathrm{time}\;\mathrm{zero}\right)}\times 100$ for Ti ≥ Tz;

$\mathrm{Growth}\;\mathrm{percentage}=\frac{\mathrm{Ti}\;\left(\mathrm{test}\;\mathrm{growth}\;\mathrm{in}\;\mathrm{the}\;\mathrm{presence}\;\mathrm{of}\;\mathrm{compound}\;\mathrm{at}\;10\;µM\;\right)-\mathrm{Tz}\;\left(\mathrm{time}\;\mathrm{zero}\right)}{\mathrm{Tz}\;\left(\mathrm{time}\;\mathrm{zero}\right)}\times 100$ for Ti < Tz.

where values of 100, 40, 0, −40, and −100 mean no GI, 60% GI, no net G, 40% lethality, and all cells are dead, respectively.

Data are expressed as a one-dose mean graph of growth percentages including the mean, delta, and range.

#### 3.2.2. VEGFRx Kinase Assay

The kinase assay was performed according to BPS Bioscience^®^ kinase assay kit protocols [17,74,75,76,77]. Briefly, 5x Kinase Buffer 1, ATP and Poly-(Glu,Tyr 4:1) (10 mg/mL) in case of FLT1 (Cat. No. 78019)/50x PTK substrate in case of KDR (Cat. No. 40325)/MBP (5 mg/mL) in case of FLT3 (Cat. No. 79797) were thawed and the master mixture (25 µL per well: N wells x (5x Kinase Buffer 1 + ATP (500 µM) + substrate + water)) was prepared. The master mixture (25 μL) was added to every well then inhibitor solution of each well, labeled as “Test Inhibitor”, was added. For the “Positive Control” and “Blank”, the same solution without inhibitor (Inhibitor buffer) was added. To the wells designated as “Blank”, 1x Kinase Buffer 1 was added. FLT1/VEGFR2/FLT3 enzyme was thawed on ice and diluted with 1x Kinase Buffer 1 then added to the wells designated “Positive Control” and “Test Inhibitor Control”. The plate was incubated at 30 °C for 45 min then ADP-Glo reagent/Kinase-Glo Max reagent was added to each well. The plate was covered with aluminum foil and incubated at room temperature for 15 min. The luminescence was measured using the microplate reader. In case of FLT1 and FLT3, the plate was incubated for 45 min, then Kinase Detection reagent was added to each well and the plate was covered with aluminum foil and incubated at room temperature for another 45 min. The luminescence was measured immediately using the Tecan–spark^®^ reader. A “Blank” value was subtracted from all readings. All samples and controls were tested in duplicate, and IC_50_s were represented as ±SD. Sorafenib was used as a reference drug.

#### 3.2.3. Cell Lines and Reagents

All cell lines were purchased from Nawah Scientific Company (Almokattam Mall, Cairo, Egypt) except A2780 and A2780CP, which were a kind gift from Prof. Jan Brabek (BIOCEV, Vestec u Prahy, Czech Republic). Cells were grown in DMEM medium (BioWhittaker™, Walkersville, MD, USA) or RMPI-1640 supplemented with bovine serum albumin (10%, Life Science Group L, Bedford, UK, Cat No: S-001B-BR) and with 100 IU/mL penicillin/streptomycin (100 µg/mL) (Lonza, Walkersville, MD, USA, 17-602E). Doxorubicin (D1515), sorafenib (SML2653), and JS-K (J4137) were obtained from Sigma-Aldrich, solubilized in DMSO, and kept at −20 °C as a stock solution. DAF-FM™ diacetate was purchased from Invitrogen (Cat. No. D-23844, Waltham, MA, USA). The tested compounds were prepared in dimethyl sulfoxide (10 mM stock) (DMSO Cat. No. 20385.02, Serva, Heidelberg, Germany) and stored at −20 °C.

##### The Initial Screening and Cell Viability by MTT Assay

According to the procedures of Skehan et al. (with minor modifications) [78,79], the cancer or normal cells were seeded in a 96-well plate (100 µL/well). After overnight incubation at 37 °C and 5% CO_2_, the cells were incubated with 50 µM of each tested compound or DMSO (0.5% *v*/*v*). After 48 h of incubation, MTT (3-(4,5-dimethylthiazoyl)-2,5-diphenyl-tetrazolium bromide) 5 mg/mL Phosphate-Buffered Saline (PBS) was added, and the plate was incubated for 4 h. After that, acidified sodium dodecyl sulfate (SDS) solution (10% SDS containing 0.01N HCl in 1× PBS) was used to solubilize formazan crystals. The absorbance was measured after 14 h of incubation at λ_570–630_ nm by a Biotek plate reader (Gen5™). To determine the IC_50_ for the most active compound **12b**, the cells were treated with serial dilutions of the **12b** compound for 48 h, then the viability was detected using MTT as mentioned above.

##### Selectivity of **12b** towards Cancer Cells

According to the procedures of Bézivin et al. (with minor modifications) [80,81,82], the selectivity of compound **12b** against cancer cell lines was evaluated by seeding human skin fibroblast cells (HSF) at 5 × 10^4^ cells/mL in a 96-well plate and incubating them overnight at 37 °C and 5% CO_2_. On the second day, serial dilution of the compound **12b** was added. After 48 h of incubation, the viability of HSF cells was determined by MTT assay as mentioned above and the selectivity index (SI) was calculated according to Equation (1):

Equation (1) Calculation of selectivity index.

(1) $$\mathrm{SI}=\frac{{\mathrm{IC}}_{50}\;normal\;cells}{{\mathrm{IC}}_{50}\;cancer\;cell}$$

##### Cell-Cycle Analysis

According to Gray et al. [83], HepG2 cells were seeded in a 6-well plate (2 mL/well) and incubated at 37 °C and 5% CO_2_ for 12 h. Cell synchronization at the G1 phase was performed by incubating the cells with serum-free medium for 12 h, then cells were treated with 5.5 or 11 μM of **12b**. After 48 h of incubation, cells were washed twice with ice-cold 1× PBS and subjected to trypsinization. The detached cells were centrifuged at 500× *g* for 5 min at 4 °C. Then, the cell pellet was resuspended in ice-cold 1× PBS and fixed with absolute ethanol. After 2 h of incubation at −20 °C, the fixed cells were washed with ice-cold 1× PBS. The cell pellet was resuspended in propidium (PI)/RNase (BD Biosciences, BDB550825, Franklin Lakes, NJ, USA) and incubated for 30 min at room temperature (RT) in the dark, then the DNA content in each phase was measured by flow cytometry using BD FACS Calibur [84].

##### Apoptosis Analysis

Briefly, HepG2 cells (10^6^ cells/mL) were treated either with **12b** (5.5 or 11 µM) or with DMSO for 24 h, and then were collected after trypsinization and washed twice with 1× PBS. The induction of apoptosis was analyzed by staining cells with PE-Annexin-V [85] in the presence of PI and analyzed using the BD FACS Calibur Flow cytometer.

##### Detection of Nitric Oxide Level by DAF-FM Diacetate

HepG2 cells were collected, and the number was adjusted at 10^6^ cells/test, and then they were stained with 5 µM DAF-FM™ diacetate (Invitrogen, Cat. No. D-23844) for 60 min at 37 °C and 5% CO_2_. After that, the stained cells were washed to remove the excess probe, and then were re-incubated for another 30 min. Following that, the stained cells were treated either with **12b** (5.5 or 11 µM) or JS-K as a positive nitric oxide donor or DMSO as a negative control for another 1 h. The fluorescence was detected by flow cytometry using the FITC detection system in a BD flow Cytometer [86,87,88].

##### RT-PCR for Gene Expression

HepG2 cells were treated either with DMSO, 5.5 or 11 µM of **12b** for 12 h, and then the total RNA was extracted by TRIzol™ Reagent (Invitrogen, Cat. No. 15596018). The RNA concentration and purity were evaluated using a Thermo Scientific™ NanoDrop™ One Microvolume UV-Vis Spectrophotometer. Four µg RNA was reverse transcribed to cDNA using the RevertAid First Strand cDNA Synthesis kit. The level of *N-cadherin*, *E-cadherin*, *BCL-2*, *Bax*, and *GAPDH* was detected using the SensiFAST™ SYBR^®^ High-ROX Kit with the StepOne™ Real-Time PCR System (Applied Biosystems™). The expression level was firstly normalized to the level of *GAPDH*, then calculated as 2^−(ΔΔCt)^, where ΔC_t_ **_12b_** = (C_t_ ™ C_t GAPDH_); ΔC_t DMSO_ = (C_t DMSO_ ™ C_t GAPDH_); ΔΔC_t_ = (ΔC_t_ **_12b_** ™ ΔC_t DMSO_). C_t_ is the cycle threshold. Primers were synthesized by Eurofins Scientific and are listed in Table 4 [58,89].

##### Western Blot Analysis

The cells were seeded (2.5 × 10^5^ cells/mL) and treated as described above. After 48 h of incubation with compound **12b**, cells were lysed by RIPA buffer containing 1x protease and phosphatase inhibitor cocktail (Thermo Scientific™, Waltham, MA, USA). The supernatant was collected after centrifugation at 4 °C and the Pierce BCA Protein Assay Kit (Thermo Scientific™, 23225, Waltham, MA, USA) was used for protein concentration detection. Proteins were then separated using SDS-PAGE at 100 V for 45 min. After that, the proteins were transferred to a nitrocellulose membrane. The membrane was subjected to a suitable primary antibody overnight. Then, an appropriate HRP-conjugated secondary antibody was used. *GAPDH* was used as a loading control. The membrane was developed using WesternBright^®^ ECL (Advansta Inc., K-12045-D20, San Jose, CA, USA), and the signal was detected using ChemiDoc XRS^+^ (1708265, Bio-Rad Laboratories, Hercules, CA, USA). All antibodies were purchased from Cell Signaling Technology (Danvers, MA, USA) are listed in Table 5 [59].

##### ELISA Assay

The p38, MKK3, and JNK assays were performed according to the manufacturer’s ELISA assay protocols [60,61,62]. Briefly, a 96-well plate was seeded with a cell density of 10,000–40,000 cells/well. Then, 50 µL of compound **12b** or control (DMSO) was added to appropriate wells in triplicate. Following this, 50 µL of the antibody cocktail (in case of p38; Cat. No. ab221012, Abcam (Waltham, MA, USA) and MKK3; Cat. No. PEL-MKK3-S189-T, RayBiotech, Peachtree Corners, GA, USA) was added to each well. The plate was then sealed and incubated for 1–2.5 h at room temperature with gentle shaking. Each well was washed with 1X Wash Buffer PT (4 × 350 µL), and then the plate was inverted and tapped gently against clean paper towels to remove excess liquid. In case of JNK (Cat. No. ab176662, Abcam, Waltham, MA, USA), this washing step was applied before and after adding 1X rabbit anti-phospho-MKK3 antibody, then 100 µL of HRP-conjugated anti-rabbit IgG solution was added and the plate was incubated for 1 h at room temperature with gentle shaking followed by washing (4 × 350 µL). After that, 100 µL of TMB Substrate was added to each well and incubated for 15–30 min in the dark with gentle shaking. Finally, 100 µL of Stop Solution was added to each well and the plate was shaken for 1 min to mix well. The OD was recorded at 450 nm.

### 3.3. ADME/Toxicity Analysis

The web-based tool ADMETlab 2.0 was used for prediction of the physicochemical, pharmacokinetic, and drug-likeness properties of compound **12b**, with respect to the reference drug sorafenib. ADMETlab 2.0 offers a straightforward approach for comprehensive, accurate, and efficient prediction of ADMET properties, using a high-quality database of 250,000 entries covering 53 endpoints, and a multi-task graph attention framework [69,70].

### 3.4. Docking Studies

Molecular modeling studies were performed using the OpenEye^®^ scientific software (2021.spr) [19,42,90,91,92,93]. A library of pyrazolopyrimidines based on 1,2,5-oxadiaole-2-oxide were minimized using the MMFF94 minimization force field. OMEGA^®^ application was used for the generation of multi-conformers and the whole library was aligned against the reference drug sorafenib, according to shape and electrostatic potential, using the EON module. The VEGFR-2 (PDB code: 3WEZ) receptor was prepared using the Spruce^®^ module, after which the FRED^®^ application was used for docking and generating FRED Chemgauss4 scores. Then, the visualization tool, Vida^®^ application, was employed to display the potential binding interactions of the target compounds to the receptor active site. The Discovery Studio Visualization software was used to generate a 2D depiction of the protein–ligand interaction [67,68]. The docking report was generated using the Docking-Report application through the OpenEye Applications 2021.1.1 command line.

## 4. Conclusions

In this paper, the design, synthesis, and biological evaluation of a new series of pyrazolo[3,4-d]pyrimidines based on a phenylfuroxan scaffold were detailed. One-dose screening of the NCI-60 cell revealed that compounds **12a**–**c** and **14a** had the best MGI% among the tested compounds. The target compound **12b**, as the most active one, showed better anticancer activity, with IC_50_ values of 11.5, 11.6, and 13 µM against the HepG-2, A2780CP, and MDA-MB-231 cell lines, respectively, than the reference anticancer drug sorafenib. Furthermore, compound **12b** showed VEGFR-2-inhibitory activity comparable to that of sorafenib. The mechanistic study revealed that compound **12b** decreased the level of total ERK and its phosphorylated form and led to the down-regulation of the metastatic protein metalloproteinase MMP-9 and the over-expression of the cell-cycle inhibitors p21 and p27. Cell-cycle analysis demonstrated that compound **12b** can arrest cells in the subG1 phase and, thus, induce apoptosis. Compound **12b** was found to inhibit wound healing in the absence of serum, in comparison to DMSO-treated cells, as shown by a wound-healing assay, while quantitative RT-PCR for *E-cadherin* and *N-cadherin* indicated lower expression of neuronal *N-cadherin* and increased expression of epithelial *E-cadherin*, demonstrating the ability of compound **12b** to suppress metastasis. Furthermore, **12b**-treated HepG2 cells expressed a low level of anti-apoptotic *BCL-2* and over-expressed pro-apoptotic *Bax* genes. Intracellular NO measurement indicated that the NO released from **12b** was similar to that from the diazeniumdiolate JS-K reference drug, using DAF-FM DA as a fluorescence probe. The docking results indicated that the most active derivative, **12b**, overlaid well with sorafenib, while the anticancer activity of the less active analogs could be enhanced through further modification of the central aryl linker, which projected towards the protein cavity. Furthermore, the ADMET study revealed similarities between the potential compound **12b** and sorafenib, as well as its passing of the Pfizer acceptance criteria. Therefore, in this study, we presented a novel anticancer lead compound that is worth further investigation and activity improvement.

## Figures and Tables

**Table 1:** IC_50_ values for compound **12b** against different types of cancers.

Cancer	Cell Line	IC_50_ (µM)	
		12b	Sorafenib
Liver	HepG2	11.5 ± 0.5	14 ± 0.4
Ovarian	SKOV-3	18.6 ± 0.2	16.9 ± 0.2
	A2780	15 ± 0.15	10.5 ± 0.5
	A2780CP	11.6 ± 0.1	15.2 ± 0.1
Breast	MDA-MB-231	13 ± 0.2	16.6 ± 0.3
	MCF-7	22.3 ± 0.3	5.1 ± 0.4
Colon	HT-29	15 ± 0.24	11 ± 0.2

± represents the standard deviation from triplicates.

**Table 2:** EON shape and electrostatic potential similarity of target compounds with respect to the query drug sorafenib.

CPD Name	EON ET coul ^a^	EON ET pb ^b^	EON Shape Tanimoto ^c^	EON ET combo ^d^	EON Rank
Sorafenib	1	1	1	2	1
14a	0.153	0.126	0.106	0.232	2
14b	0.155	0.127	0.102	0.229	3
14c	0.153	0.126	0.103	0.228	4
6d	0.067	0.057	0.086	0.143	5
12a	0.023	0.032	0.107	0.139	6
6h	0.056	0.049	0.089	0.138	7
12b	0.012	0.019	0.117	0.136	8
6b	0.055	0.048	0.088	0.135	9
12c	0.016	0.022	0.112	0.135	10
6f	0.045	0.039	0.091	0.13	11
6a	0.02	0.032	0.078	0.11	12
6c	0.072	0.069	0.04	0.109	13
6g	0.065	0.06	0.044	0.104	14
6e	0.013	0.027	0.076	0.104	15

**^a^** Electrostatic Tanimoto using only the coulombic (coul) part of Poisson–Boltzmann (PB) electrostatics. **^b^** Electrostatic Tanimoto using full PB electrostatics. **^c^** Shape Tanimoto between the given molecule and the query. **^d^** Sum of ET-pb and EON shape Tanimoto.

**Table 3:** FRED Chemgauss4 scores of target compounds **6a**–**h**, **12a**–**c**, and **14a**–**c**.

Compound	FRED Chemgauss4 Score
**6e**	−12.8024
**6a**	−11.6995
**6b**	−11.5059
**6g**	−11.4424
**12b**	−11.3747
**6c**	−11.3160
**6h**	−11.1254
**6f**	−11.0437
**14c**	−10.9153
**12a**	−10.7872
**6d**	−10.3713
**14a**	−10.1469
**14b**	−9.9950
**12c**	−9.8026
**Sorafenib**	−18.5752

**Table 4:** Primer sequence for the genes used in RT-PCR.

Gene	Primer Sequence 5′-3′
*Bax*	GTT TCA TCC AGG ATC GAG CAG
	CAT CTT CTT CCA GAT GGT GA
*BCL-2*	CAG AGA CAG CCA GGA GAA ATC A
	TCG CCC TGT GGA TGA CTG A
*E-cadherin*	GAG TGC CAA CTG GAC CA T TCA GTA
	AGT CAC CCA CCT CTA AGG CCA TC
*N-cadherin*	GAG ATC CTA CTG GAC GGT TCG
	TCT TGG CGA ATG ATC TTA GGA
*GAPDH*	ACC ACA GTC CAT GCC ATC AC
	TCC ACC ACC CTG TTG CTG TA

**Table 5:** Antibodies used in the assay.

Antibody	Dilution	Cat. No.
β-actin	1:5000	4970
P27	1:4000	2552
P21	1:2000	2947
pERK	1:2000	4370
ERK	1:2000	4695
MMP9	1:2000	13667
P53	1:5000	9282
Anti-Rabbit IgG-HRP- linked	1:5000	7074

## Data Availability

Data is contained within the article and Supplementary Files.

## Supplementary Materials

The following supplementary materials are available online at https://www.mdpi.com/article/10.3390/ph15020246/s1, Figure S1: ^1^H NMR of 3a, Figure S2: ^1^H NMR of 3b, Figure S3: ^1^H NMR of 3c, Figure S4: ^13^C NMR of 3c, Figure S5: ^1^H NMR of 3d, Figure S6: ^13^C NMR of 3d, Figure S7: ^1^H NMR of 5a, Figure S8: ^13^C NMR of 5a, Figure S9: ^1^H NMR of 5b, Figure S10: ^13^C NMR of 5b, Figure S11: ^1^H NMR of 5c, Figure S12: ^13^C NMR of 5d, Figure S13: ^1^H NMR of 6a, Figure S14: ^13^C NMR of 6a, Figure S15: DEPT135 ^13^C NMR of 6a, Figure S16: ^1^H NMR of 6b, Figure S17: ^13^C NMR of 6b, Figure S18: ^1^H NMR of 6c, Figure S19: ^13^C NMR of 6c, Figure S20: ^1^H NMR of 6d, Figure S21: ^13^C NMR of 6d, Figure S22: ^1^H NMR of 6e, Figure S23: ^1^H NMR of 6f, Figure S24: ^13^C NMR of 6f, Figure S25: ^1^H NMR of 6g, Figure S26: ^13^C NMR of 6g, Figure S27: ^1^H NMR of 6h, Figure S28: ^13^C NMR of 6h, Figure S29: ^1^H NMR of 8b, Figure S30: ^13^C NMR of 8b, Figure S31: ^1^H NMR of 8c, Figure S32: ^13^C NMR of 8c, Figure S33: ^1^H NMR of 9a, Figure S34: ^13^C NMR of 9a, Figure S35: ^1^H NMR of 9b, Figure S36: ^1^H NMR of 9c, Figure S37: ^1^H NMR of 11, Figure S38: ^1^H NMR (D_2_O) of 11, Figure S39: ^13^C NMR of 11, Figure S40: ^1^H NMR of 12a, Figure S41: ^13^C NMR of 12a, Figure S42: DEPT135 ^13^C NMR of 12a, Figure S43: ^1^H NMR of 12b, Figure S44: ^1^H NMR of 12c, Figure S45: ^13^C NMR of 12c, Figure S46: ^1^H NMR of 14a, Figure S47: ^13^C NMR of 14a, Figure S48: ^1^H NMR of 14b, Figure S49: ^13^C NMR of 14b, Figure S50: ^1^H NMR of 14c, Figure S51: ^13^C NMR of 14c, Figure S52: ^1^H NMR of 15a, Figure S53: ^13^C NMR of 15a, Figure S54: ^1^H NMR of 15b, Figure S55: ^13^C NMR of 15b, Figure S56: One dose mean graph of 6a, Figure S57: One dose mean graph of 6b, Figure S58: One dose mean graph of 6c, Figure S59: One dose mean graph of 6d, Figure S60: One dose mean graph of 6e, Figure S61: One dose mean graph of 6f, Figure S62: One dose mean graph of 6g, Figure S63: One dose mean graph of 6h, Figure S64: One dose mean graph of 11, Figure S65: One dose mean graph of 12a, Figure S66: One dose mean graph of 12b, Figure S67: One dose mean graph of 12c, Figure S68: One dose mean graph of 13a, Figure S69: One dose mean graph of 13b, Figure S70: One dose mean graph of 13c, Figure S71: One dose mean graph of 14a, Figure S72: One dose mean graph of 14b, Figure S73: One dose mean graph of 14c, Figure S74: Docking poses of some target compounds inside VEGFR-2 active site: (A) Compound 6a; (B) Compound 14a; (C) Compound 12a, Figure S75: FRED docking report of 12b in VEGFR-2, showing fitness in the binding pocket and residues in contact, Figure S76: Radar graph of physicochemical properties of (A) compound 12b and (B) sorafenib, obtained from ADMETlab 2.0, Table S1: NCI-60 cell line growth percentage screening results for target compounds 6a–h and 12a–c, Table S2: NCI-60 cell line growth percentage screening results for compounds 11, 13a–c, and 14a–c, Table S3: ADMET properties of compound 12b, predicted using the web-based ADMETlab 2.0 tool.
