# Supplementary material for: Design, Synthesis, and Biological Evaluation of a Novel VEGFR-2 Inhibitor Based on a 1,2,5-Oxadiazole-2-Oxide Scaffold with MAPK Signaling Pathway Inhibition
Source: Pharmaceuticals (Basel). 2022 Feb 18;15(2):246. doi: 10.3390/ph15020246 (PMC8880564; doi:10.3390/ph15020246)
Supplement: Supplementary file 1 [file pharmaceuticals-15-00246-s001.zip › pharmaceuticals-1578450-supplementary.pdf]

Supporting information

1 **Spectral data**

2 **NCI-60 cell line screening**

3 **Docking studies**

4 **ADMET analysis**

1 Spectral data

1.1 3a

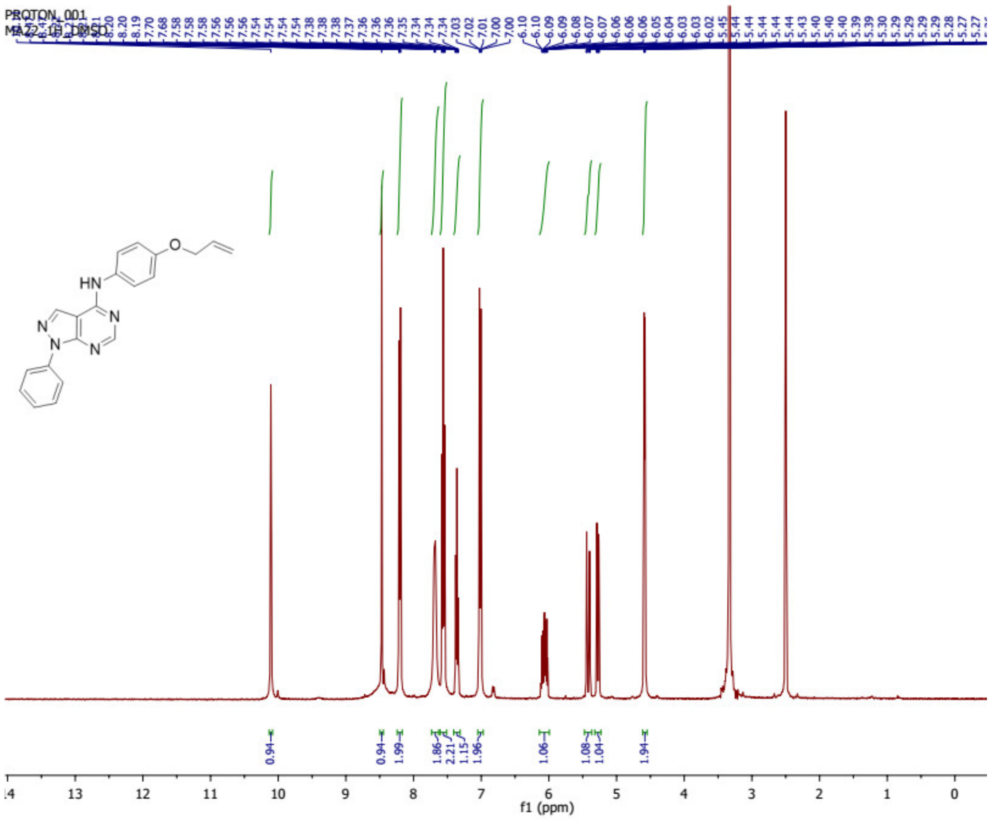

Figure S1. ^{1}H NMR of **3a**.

1.2 3b

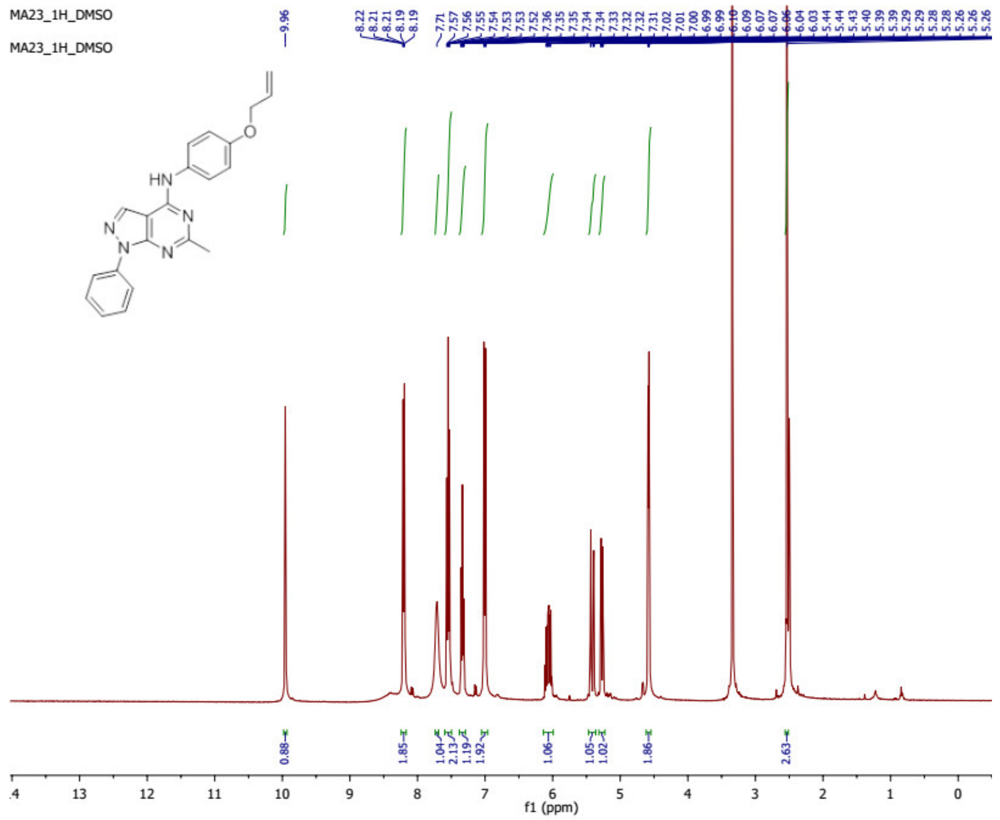

Figure S2. ^{1}H NMR of **3b**.

1.3 3c

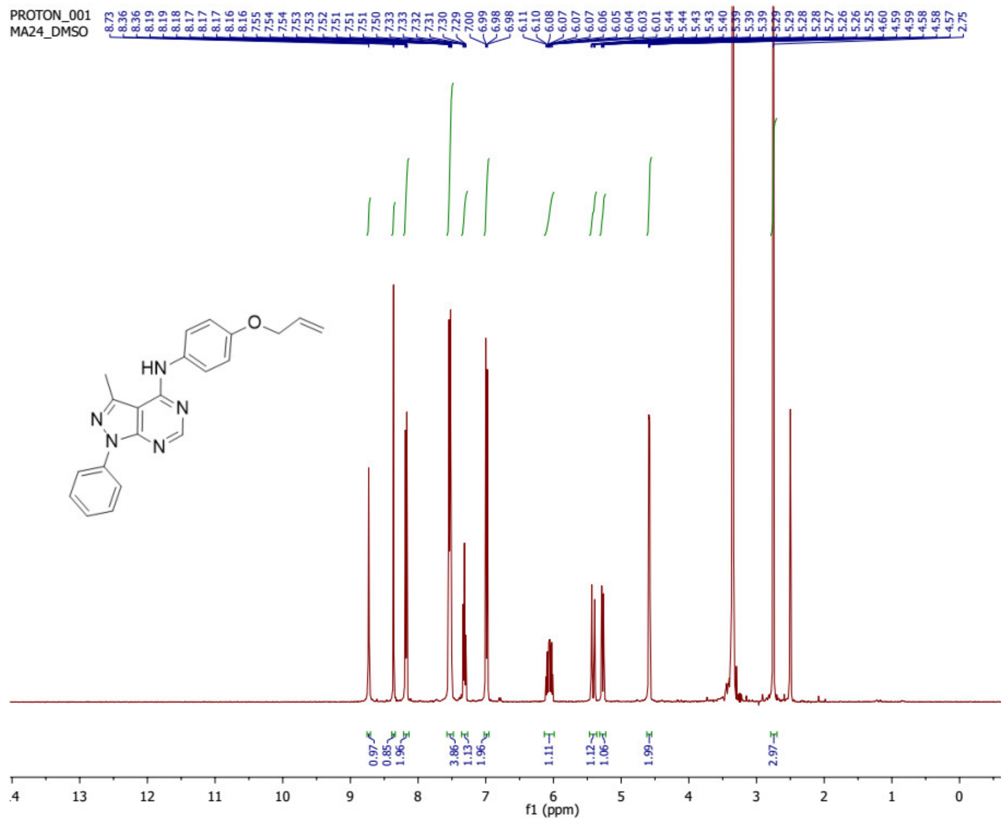

Figure S3. ^{1}H NMR of **3c**.

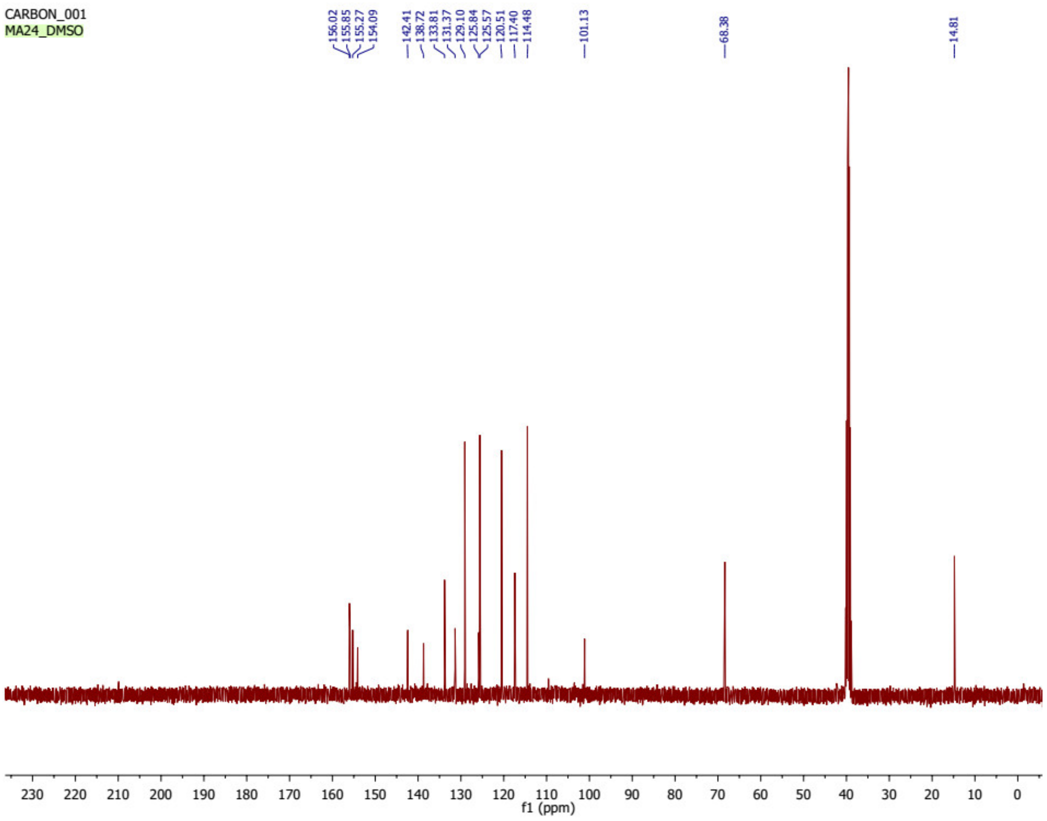

Figure S4. ^{13}C NMR of **3c**.

1.4 3d

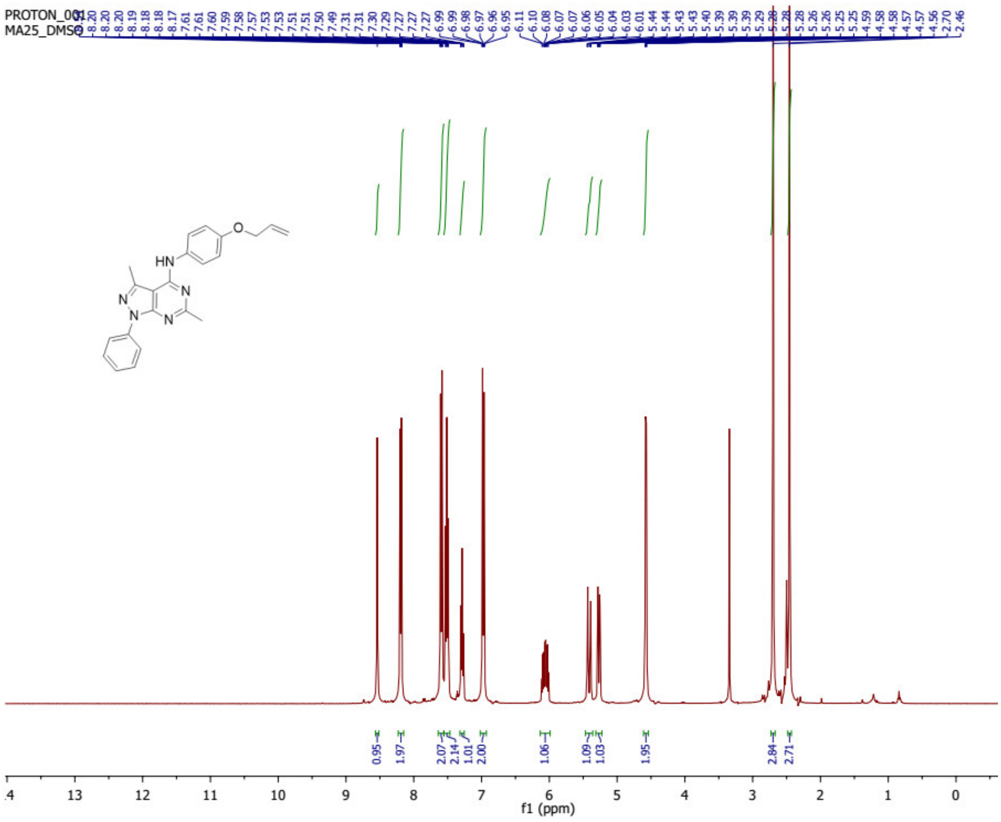

Figure S5. ^{1}H NMR of **3d**.

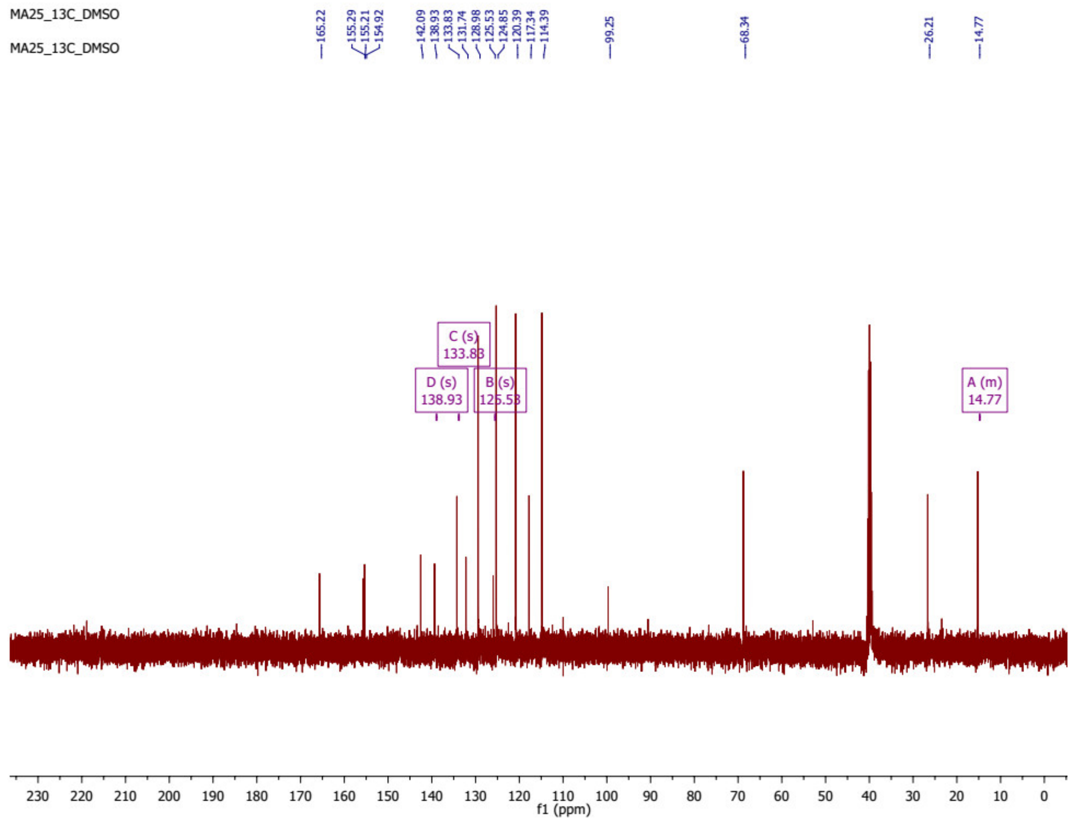

Figure S6. ^{13}C NMR of **3d**.

1.5 5a

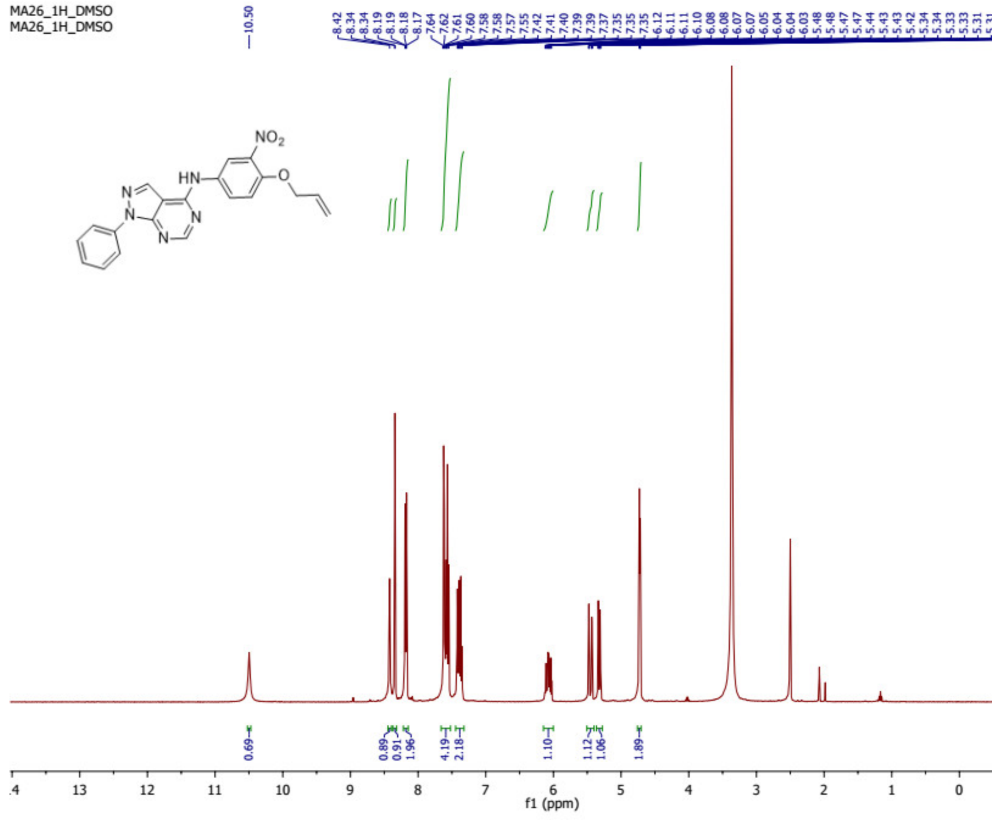

Figure S7. ^{1}H NMR of **5a**.

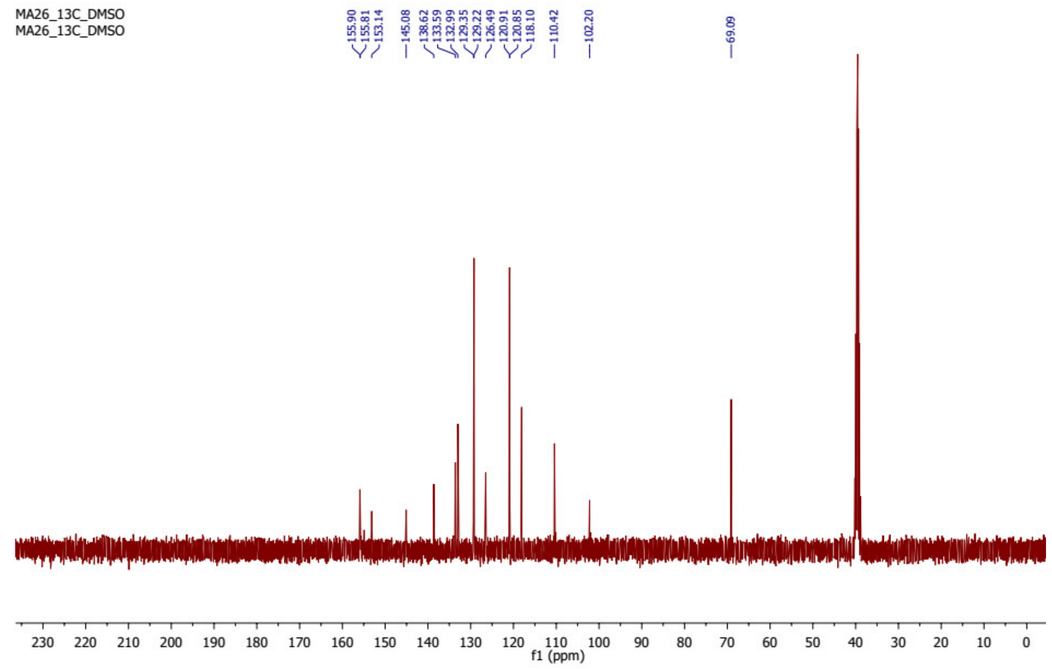

Figure S8. ^{13}C NMR of **5a**.

1.6 5b

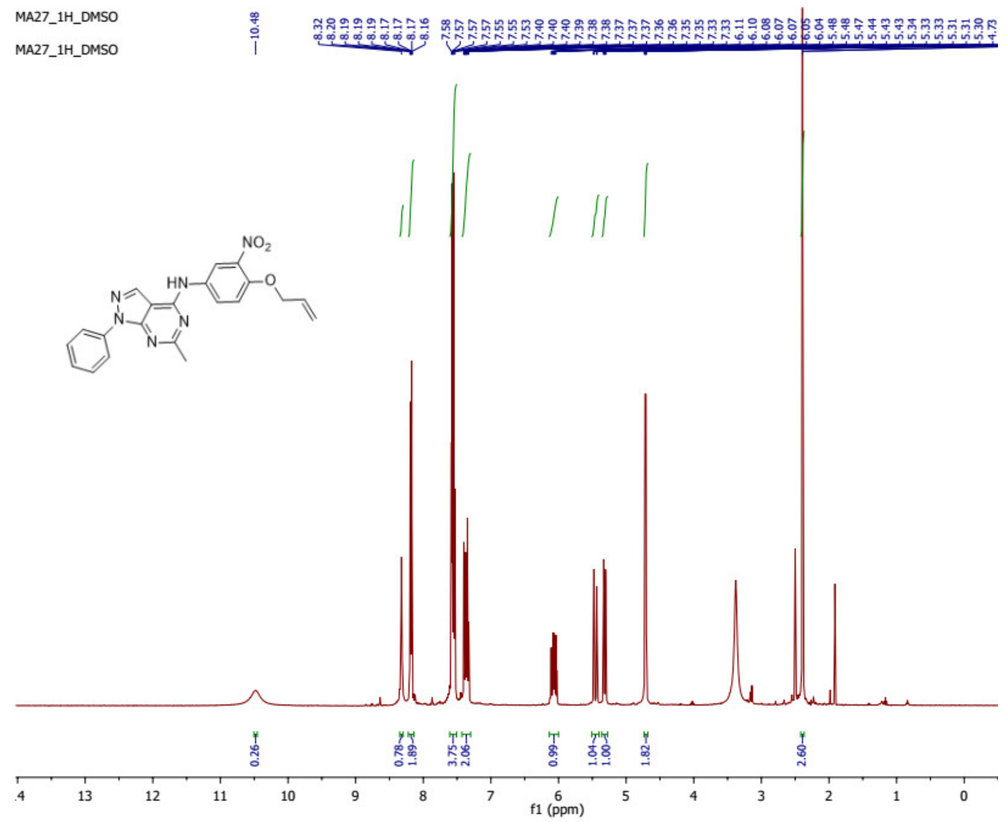

Figure S9. ^{1}H NMR of **5b**.

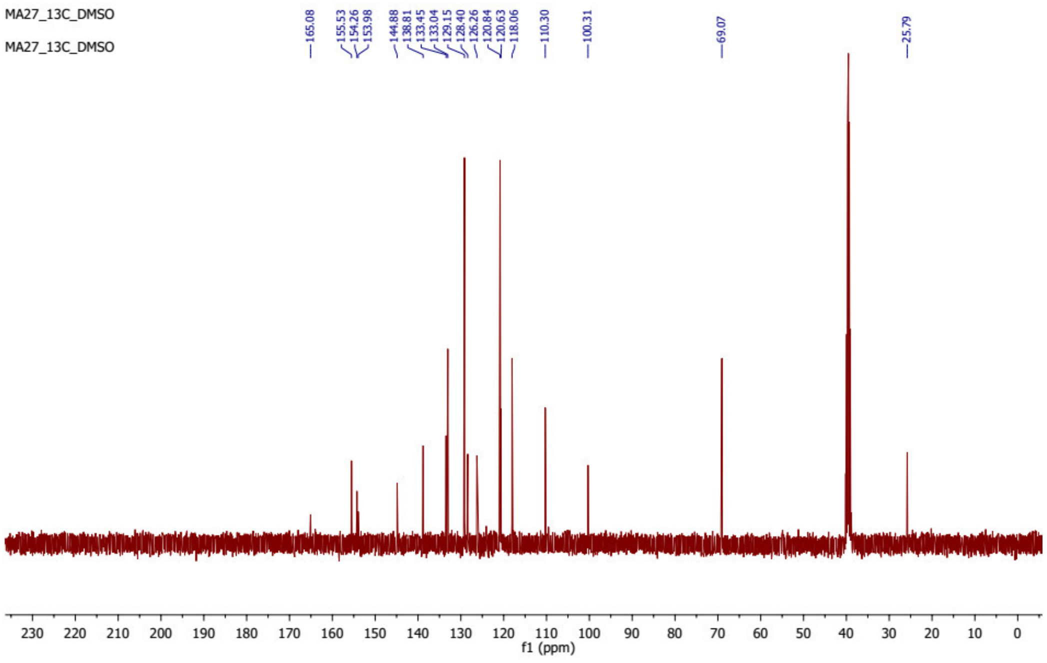

Figure S10. ^{13}C NMR of **5b**.

1.7 5c

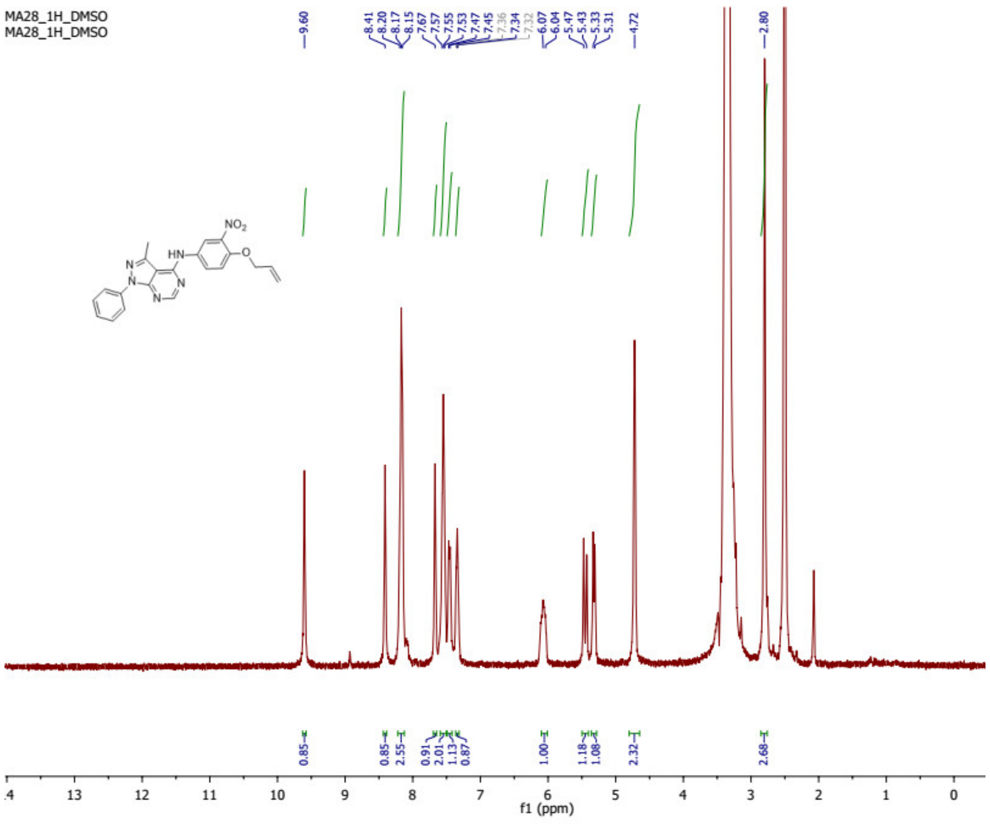

Figure S11. ^{1}H NMR of **5c**.

1.8 5d

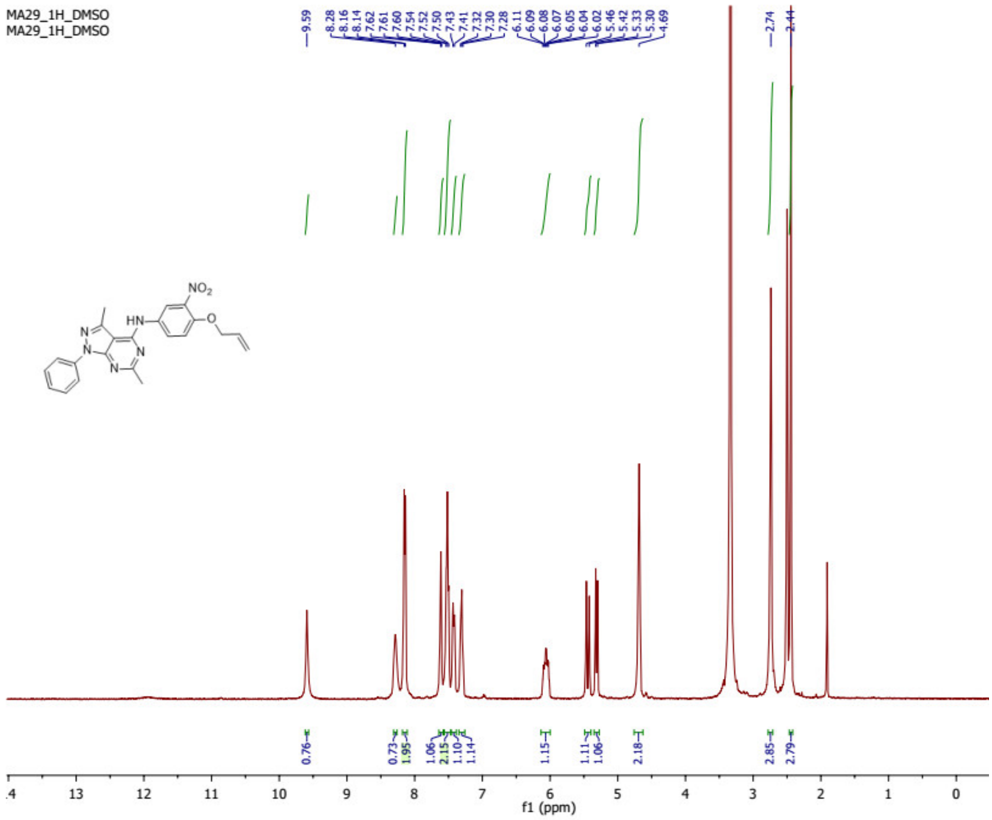

Figure S12. ^{13}C NMR of **5d**.

1.9 6a

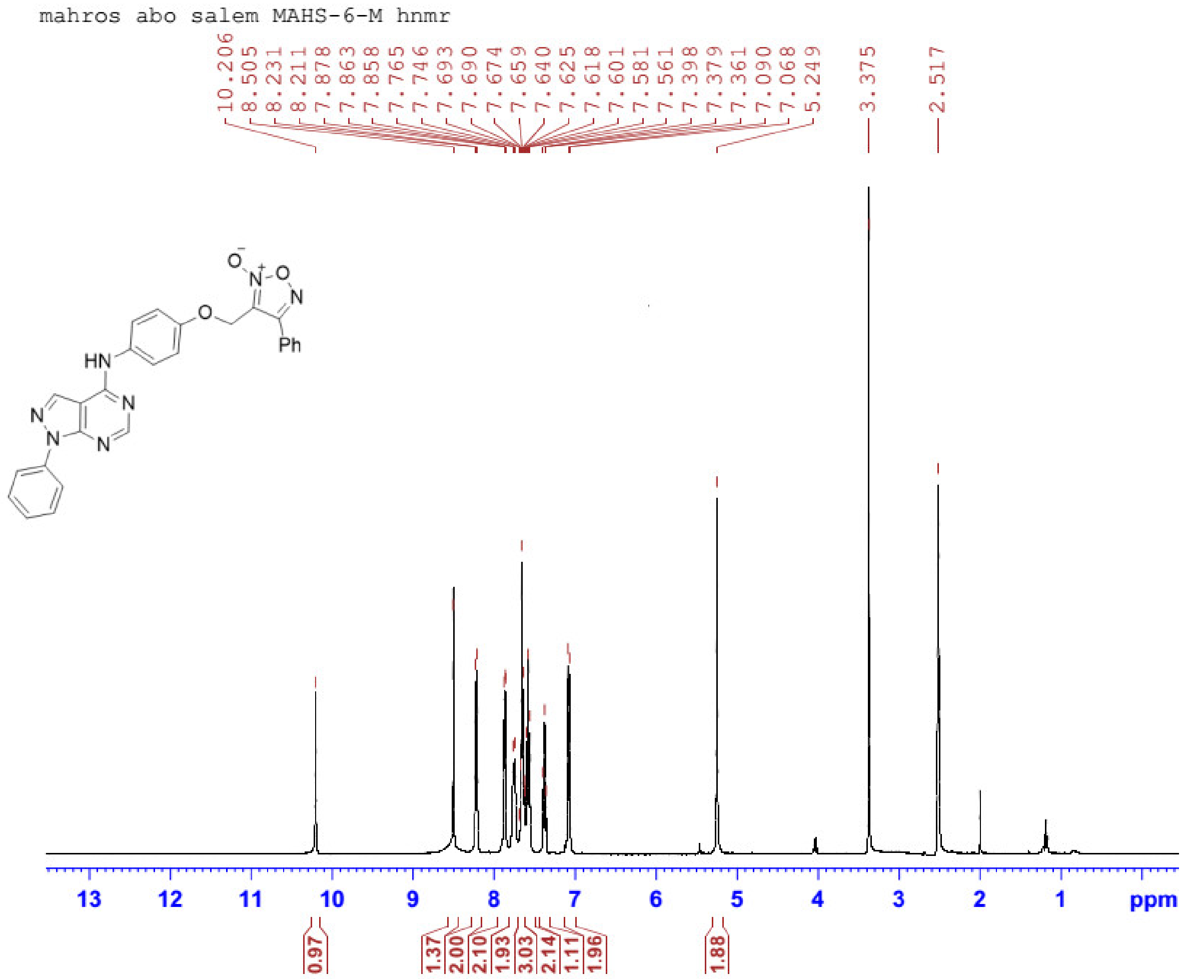

Figure S13. ¹H NMR of **6a**.

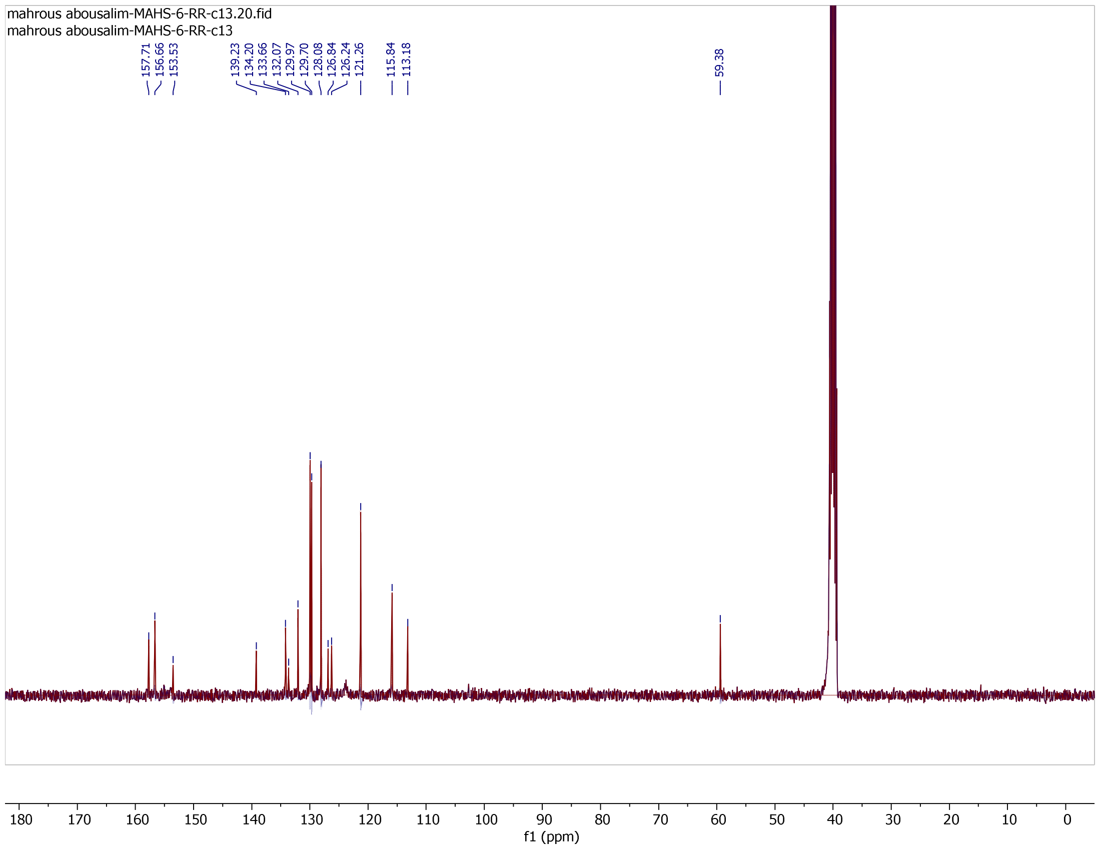

Figure S14. ¹³C NMR of **6a**.

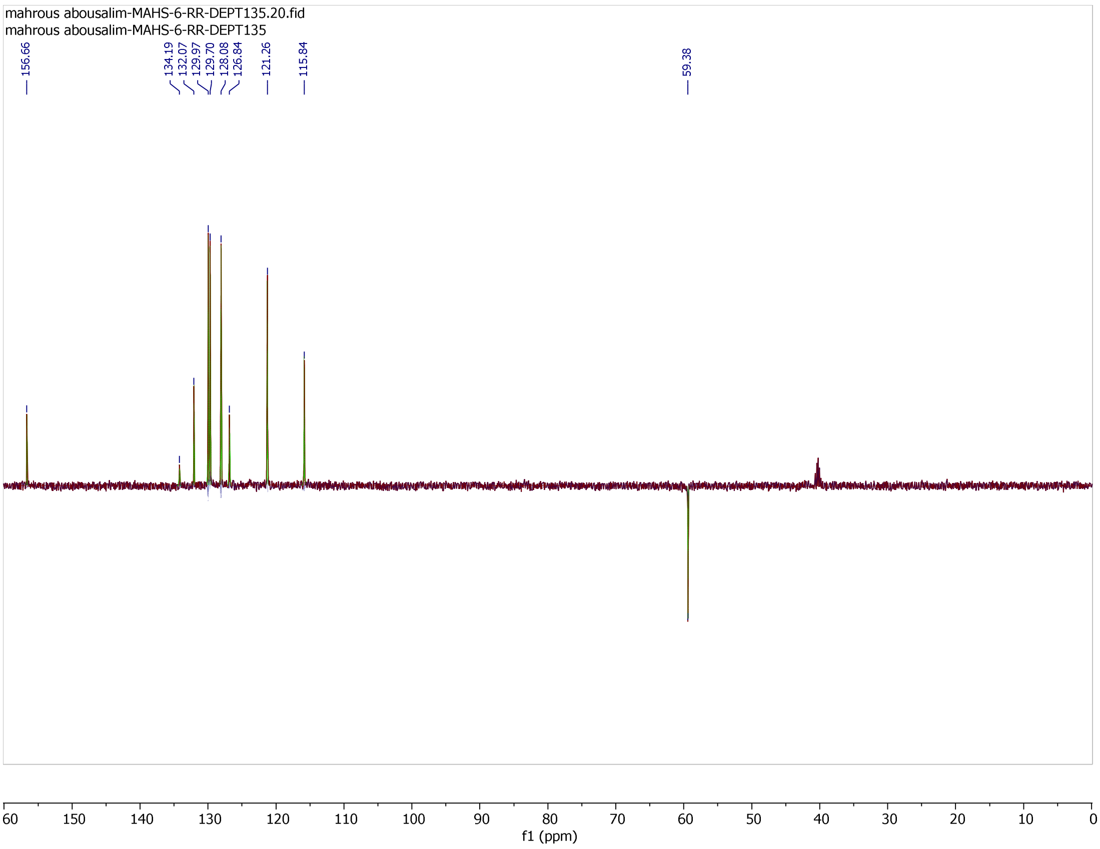

Figure S15. DEPT135 ^{13}C NMR of **6a**.

1.10 6b

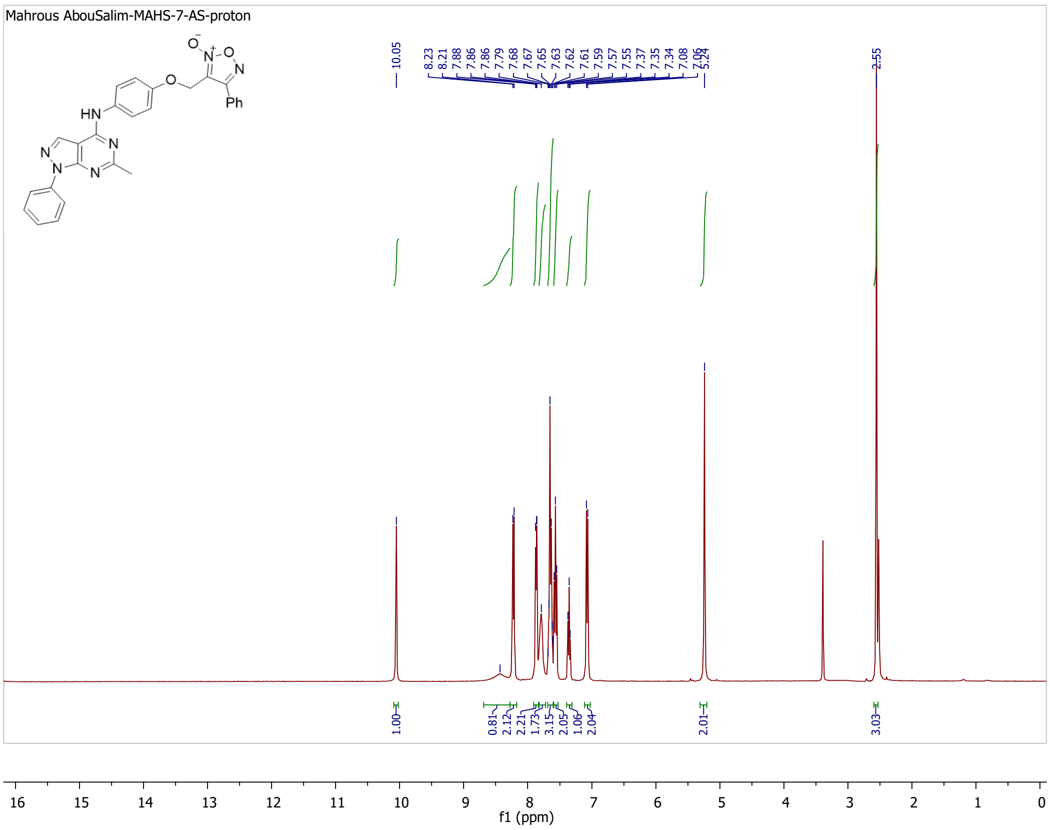

Figure S16. ^{1}H NMR of **6b**.

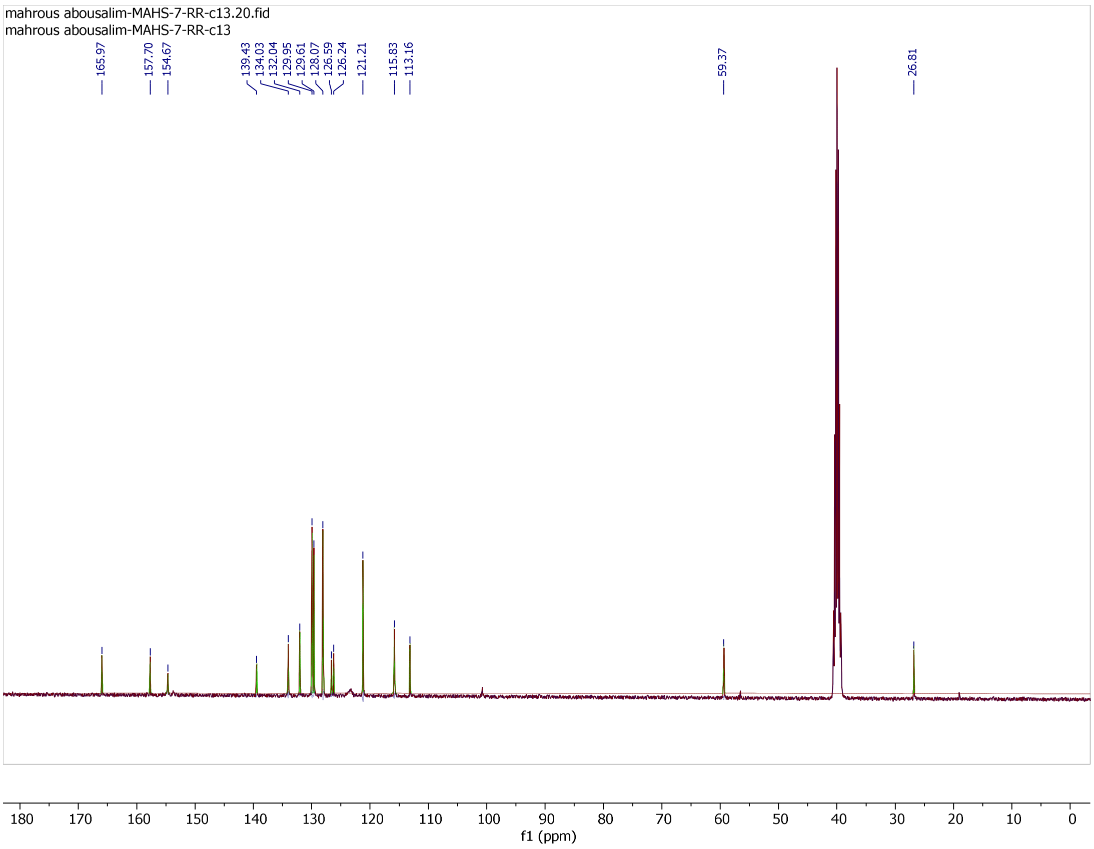

Figure S17. ^{13}C NMR of **6b**.

1.11 6c

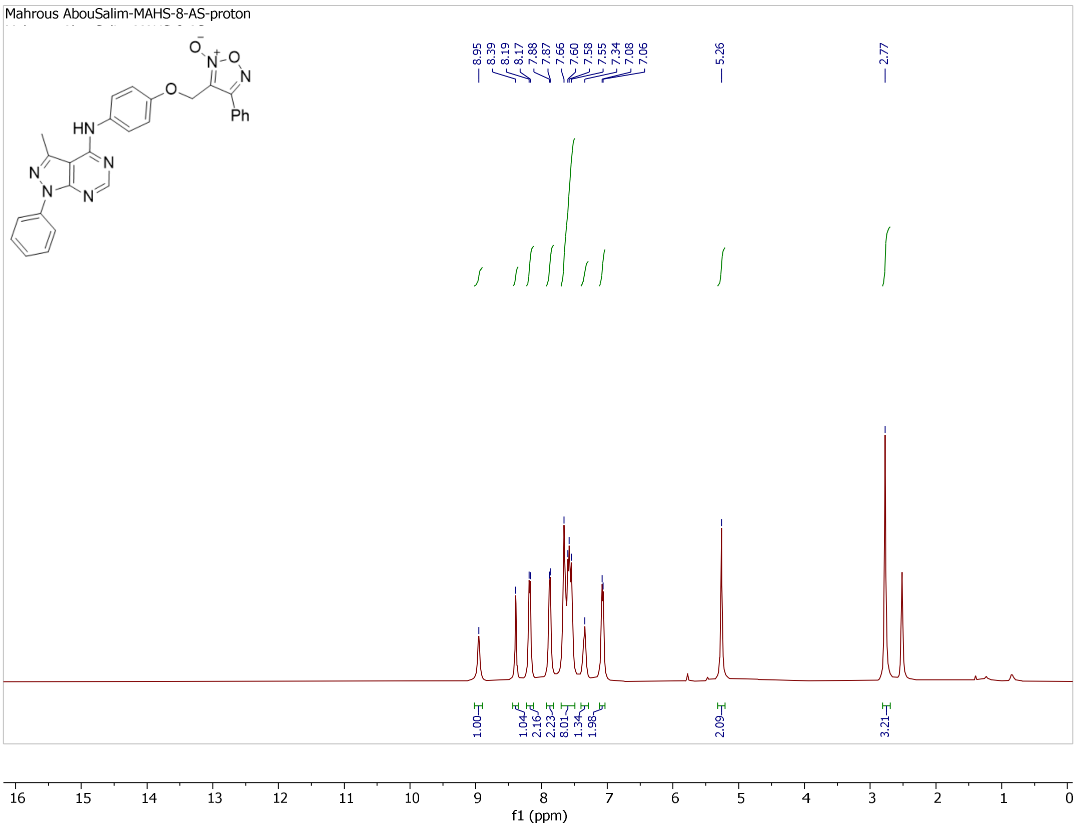

Figure S18. ^{1}H NMR of **6c**.

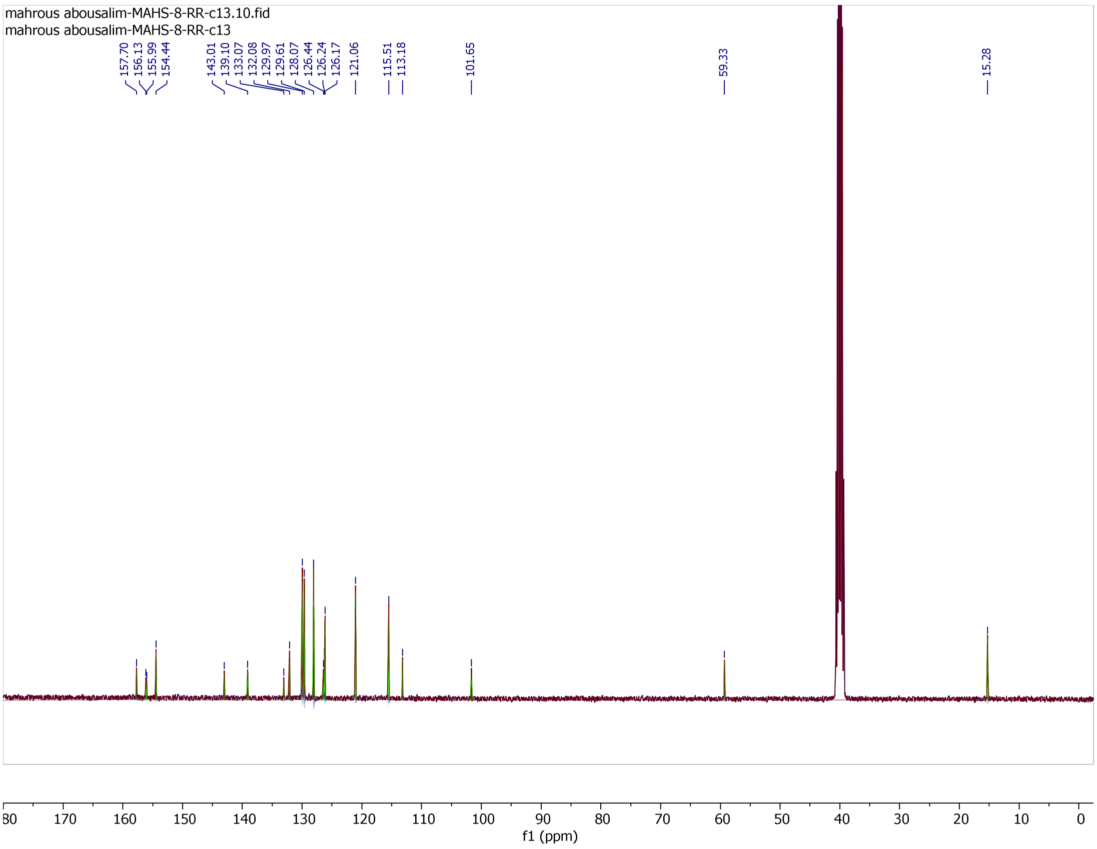

Figure S19. ^{13}C NMR of **6c**.

1.12 6d

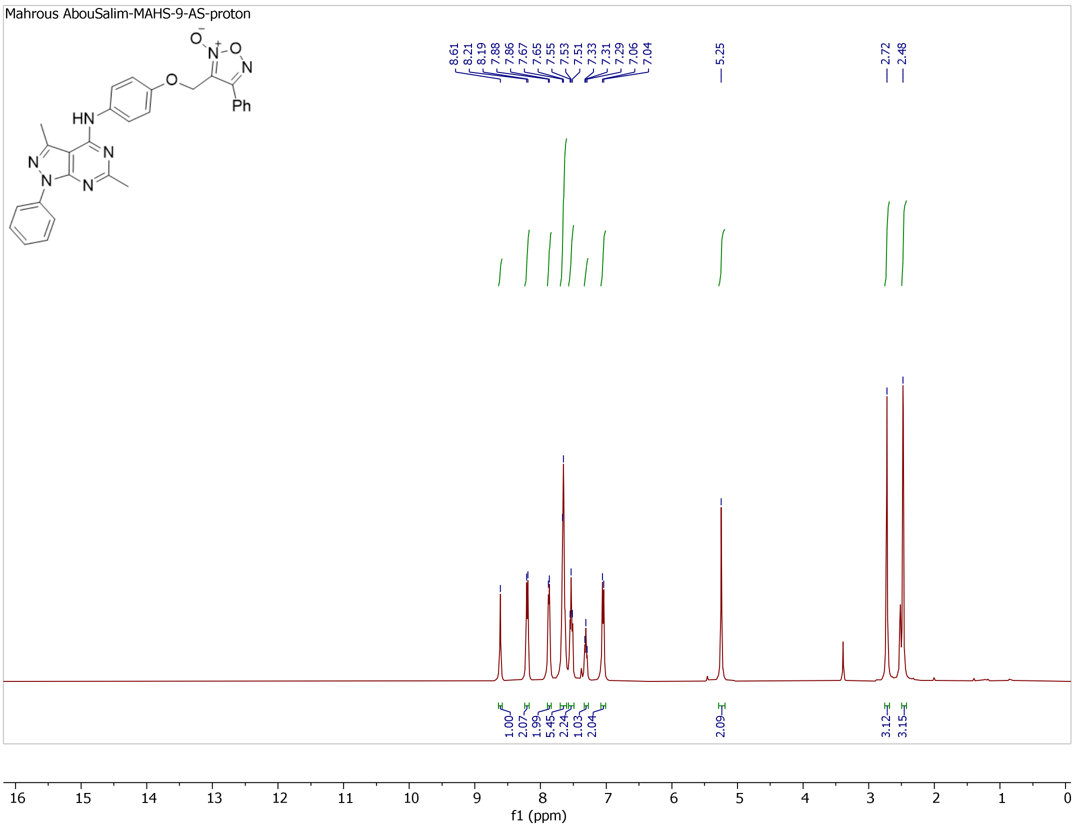

Figure S20. ^{1}H NMR of **6d**.

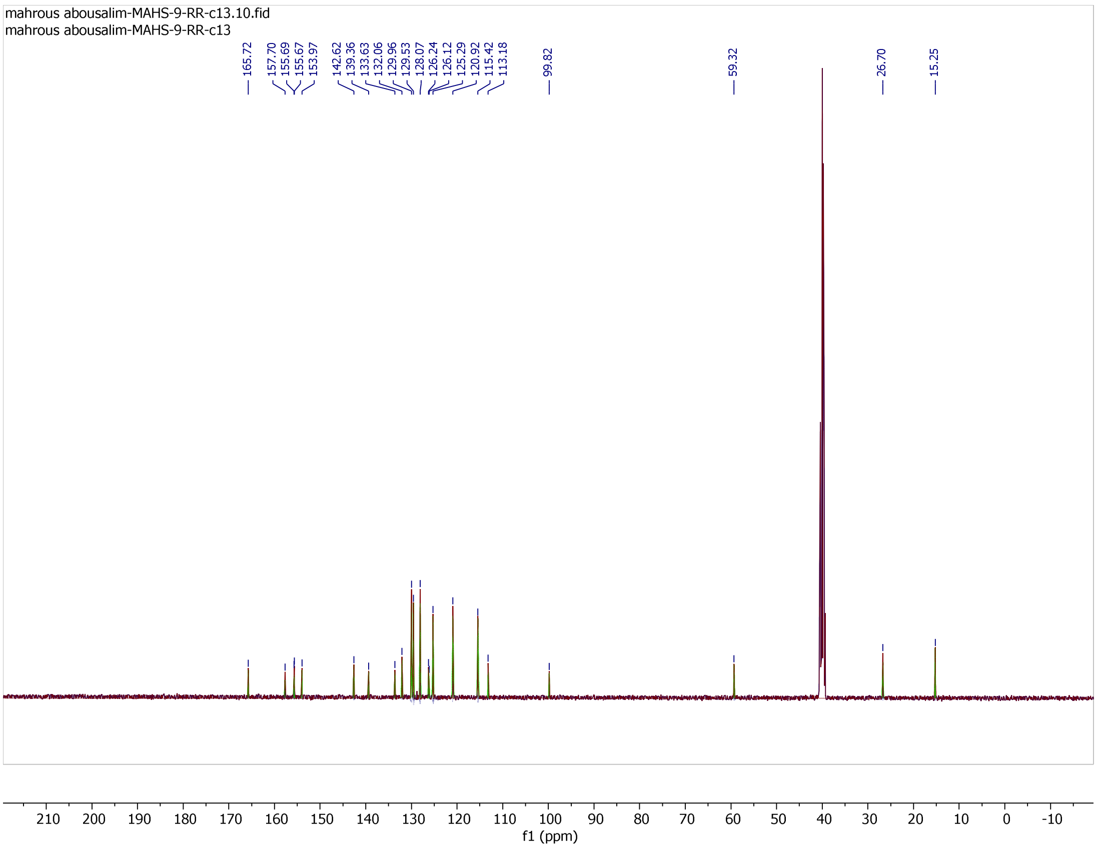

Figure S21. ^{13}C NMR of **6d**.

1.13 6e

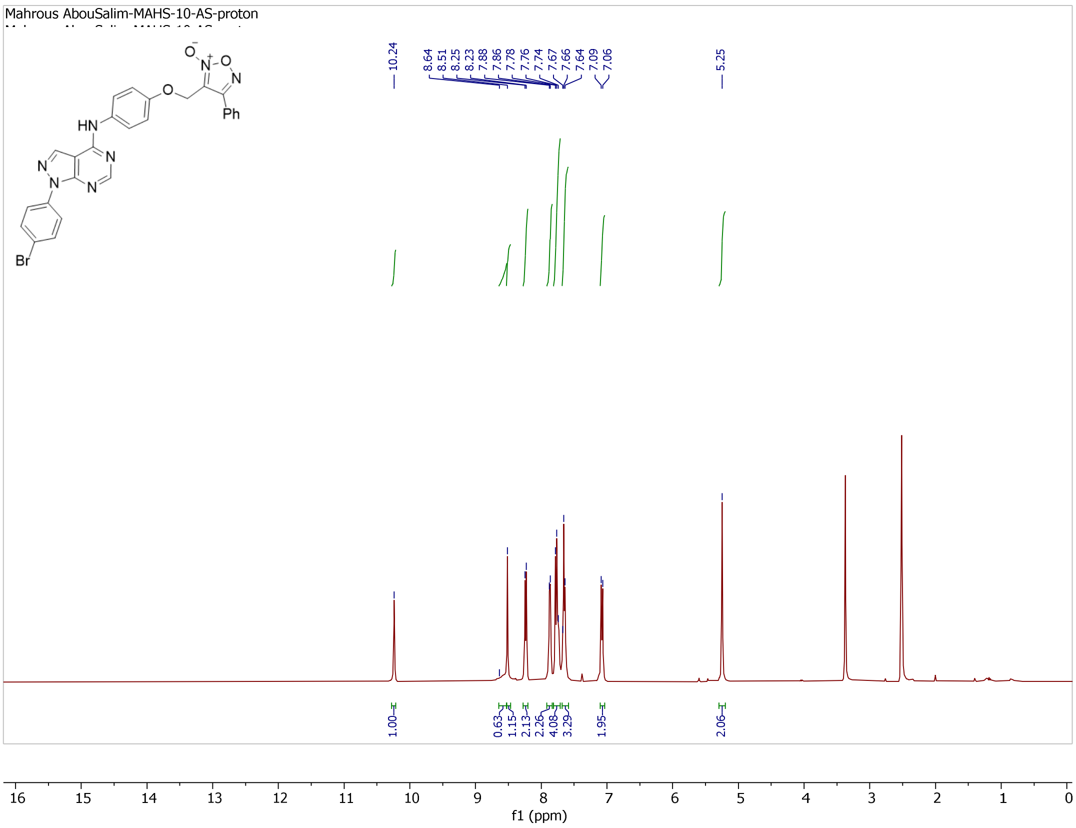

Figure S22. ^{1}H NMR of **6e**.

1.14 6f

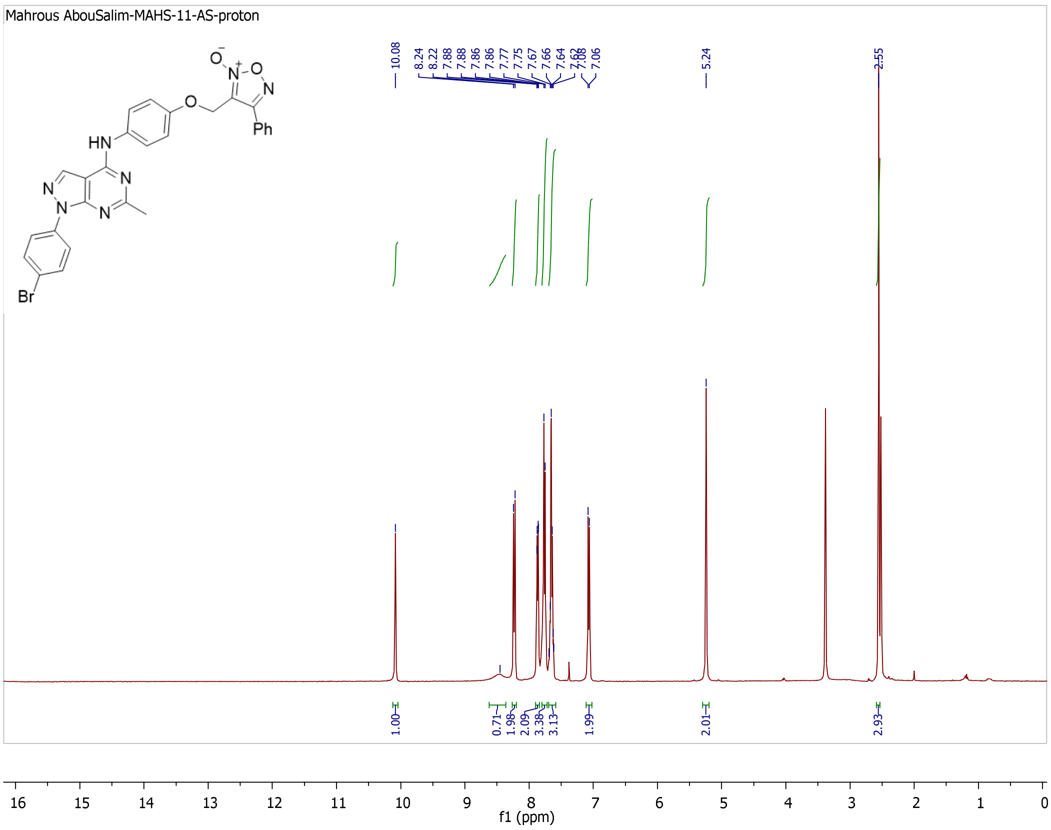

Figure S23. ^{1}H NMR of **6f**.

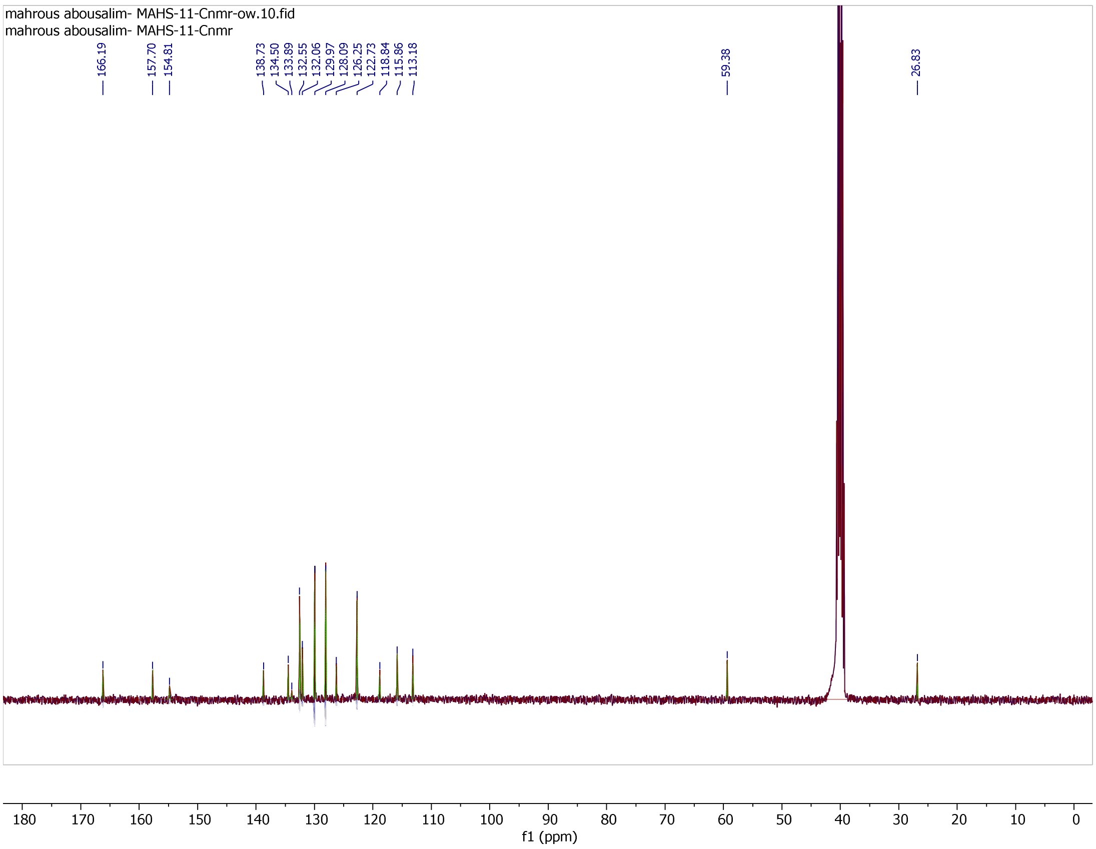

Figure S24. ^{13}C NMR of **6f**.

1.15 6g

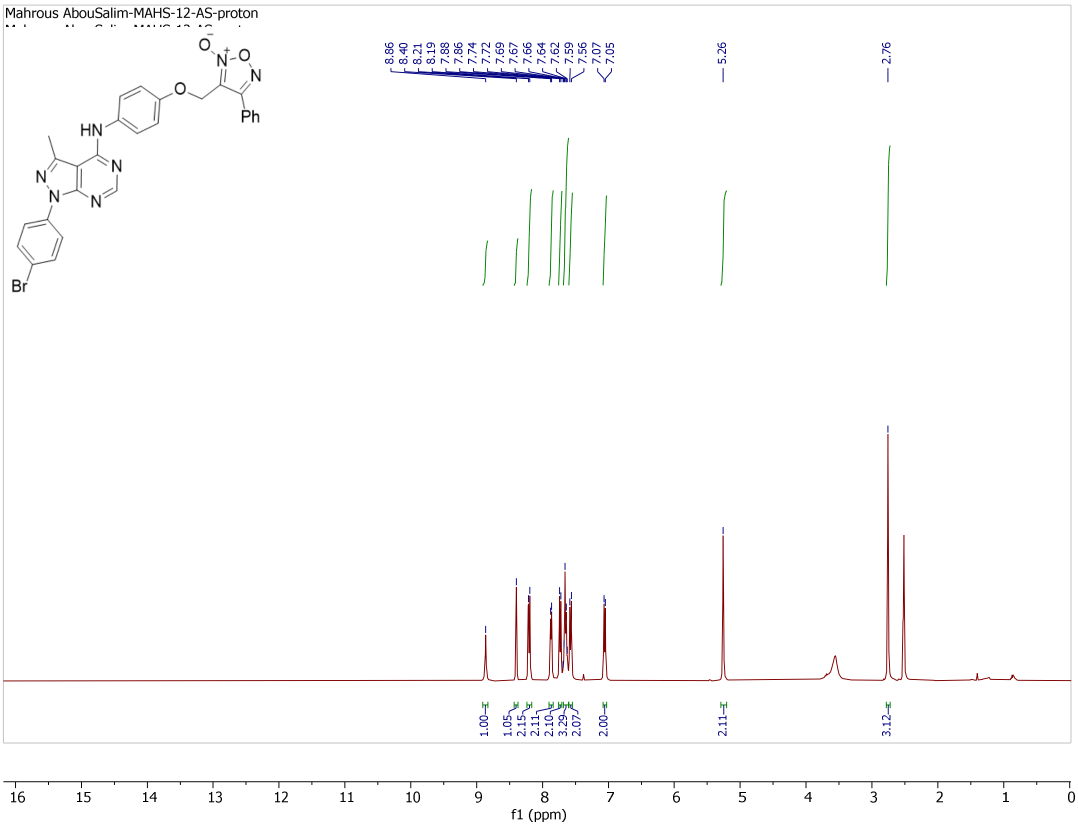

Figure S25. ^{1}H NMR of **6g**.

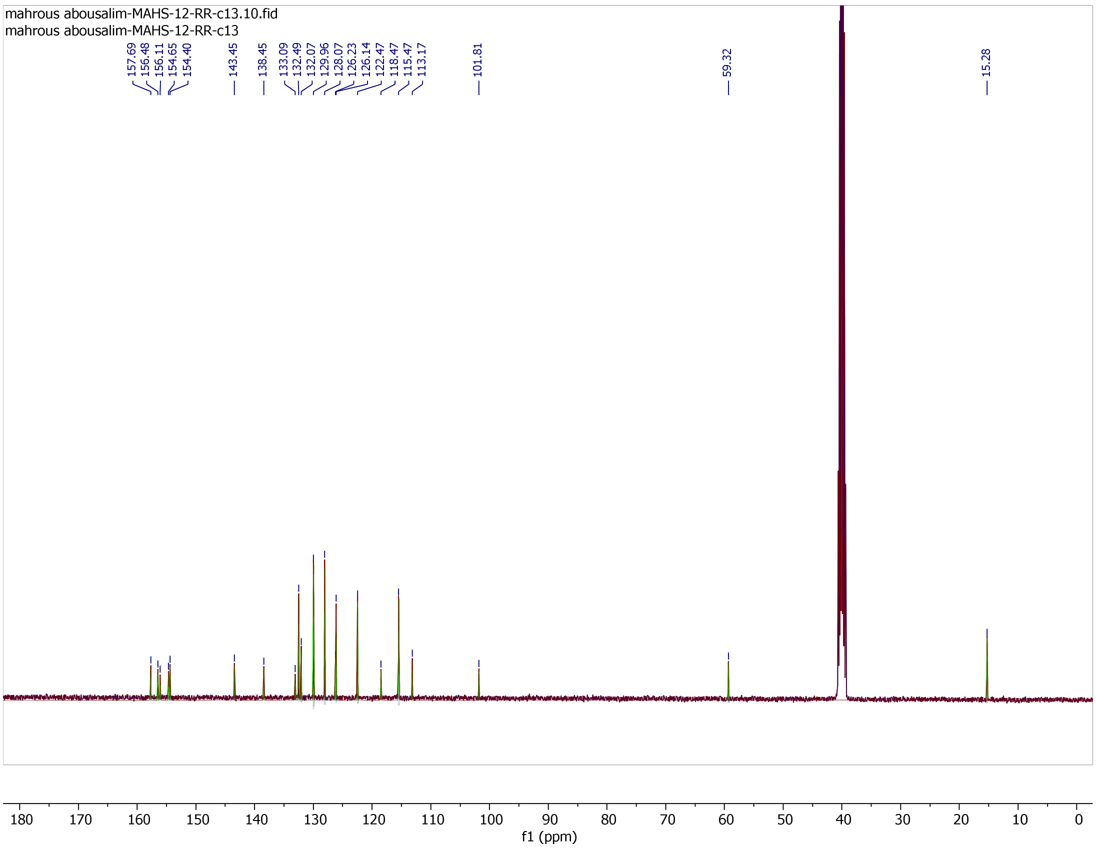

Figure 26. ^{13}C NMR of **6g**.

1.16 6h

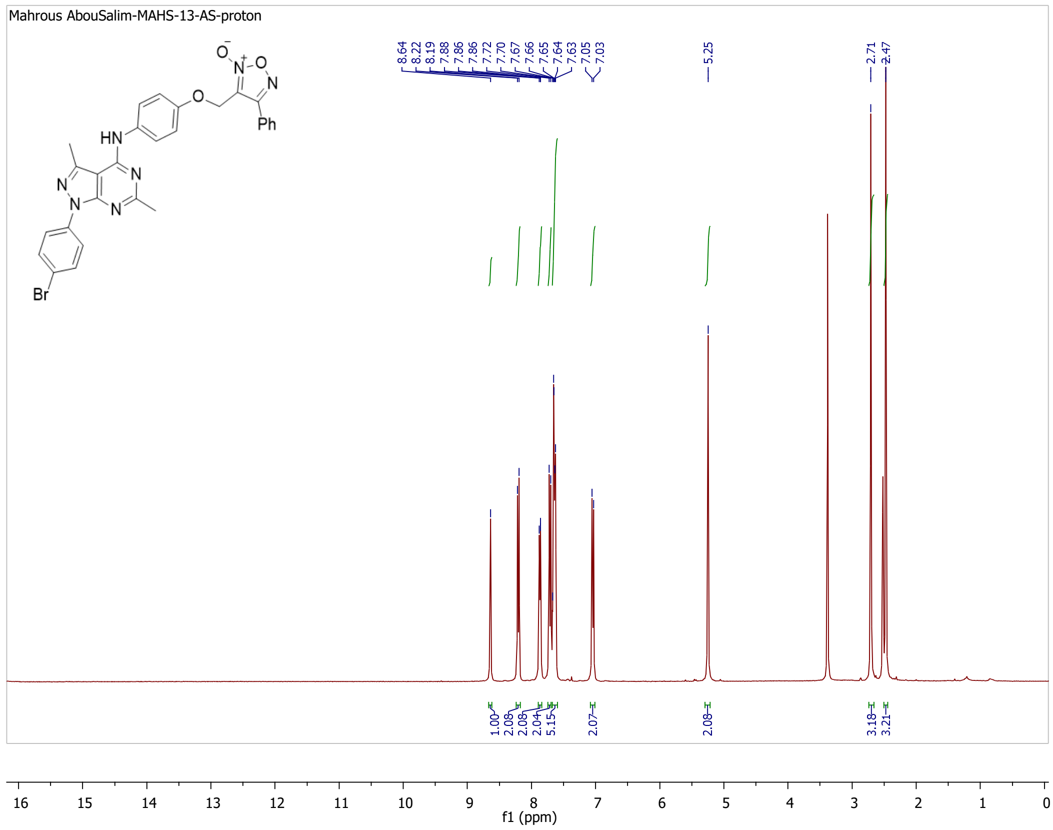

Figure S27. ¹H NMR of **6h**.

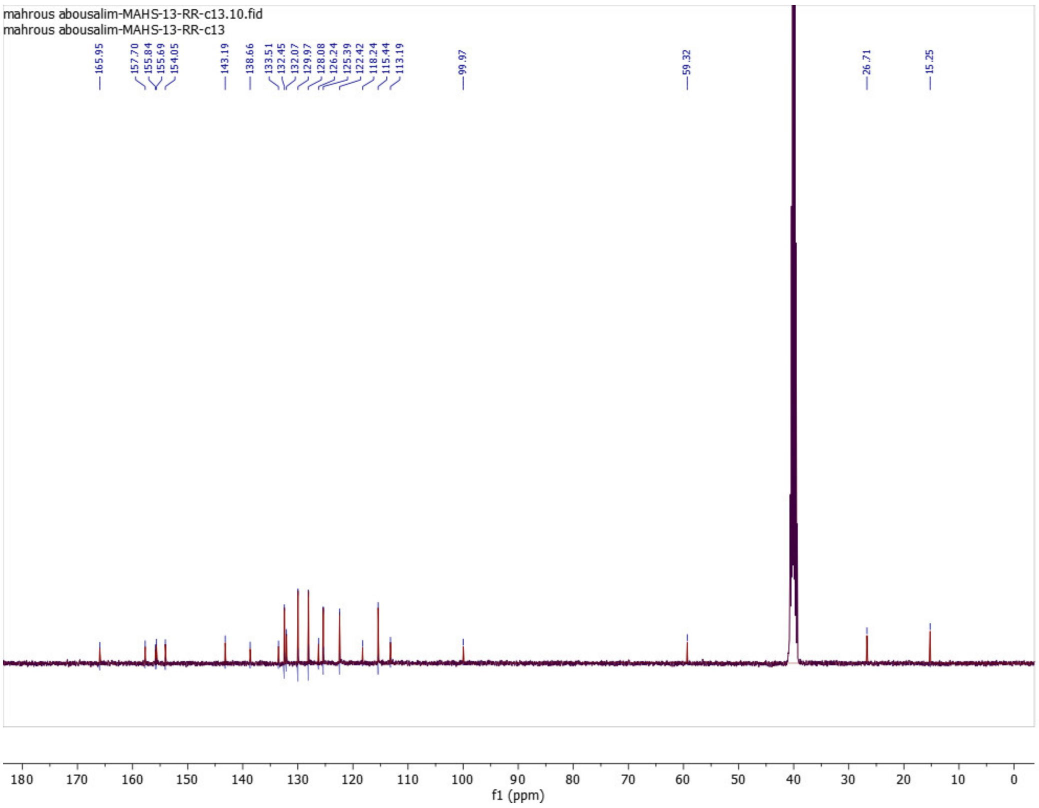

Figure S28. ¹³C NMR of **6h**.

1.17 8b

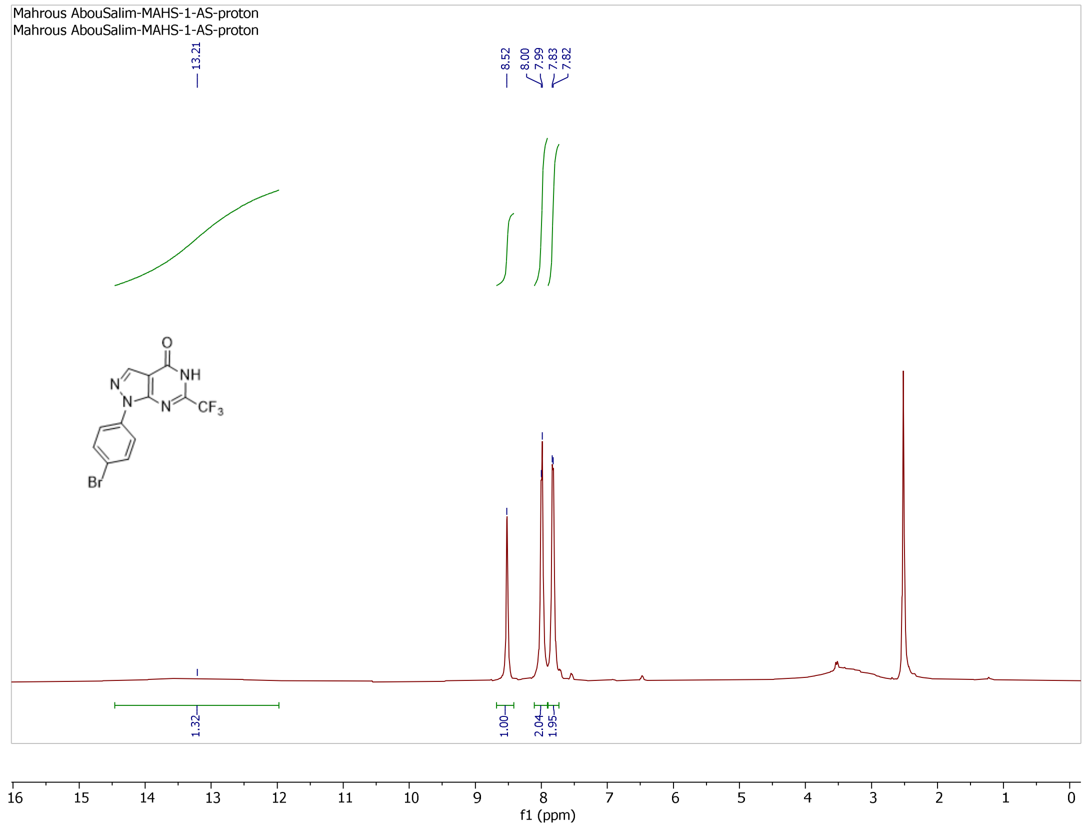

Figure S29. ^{1}H NMR of **8b**.

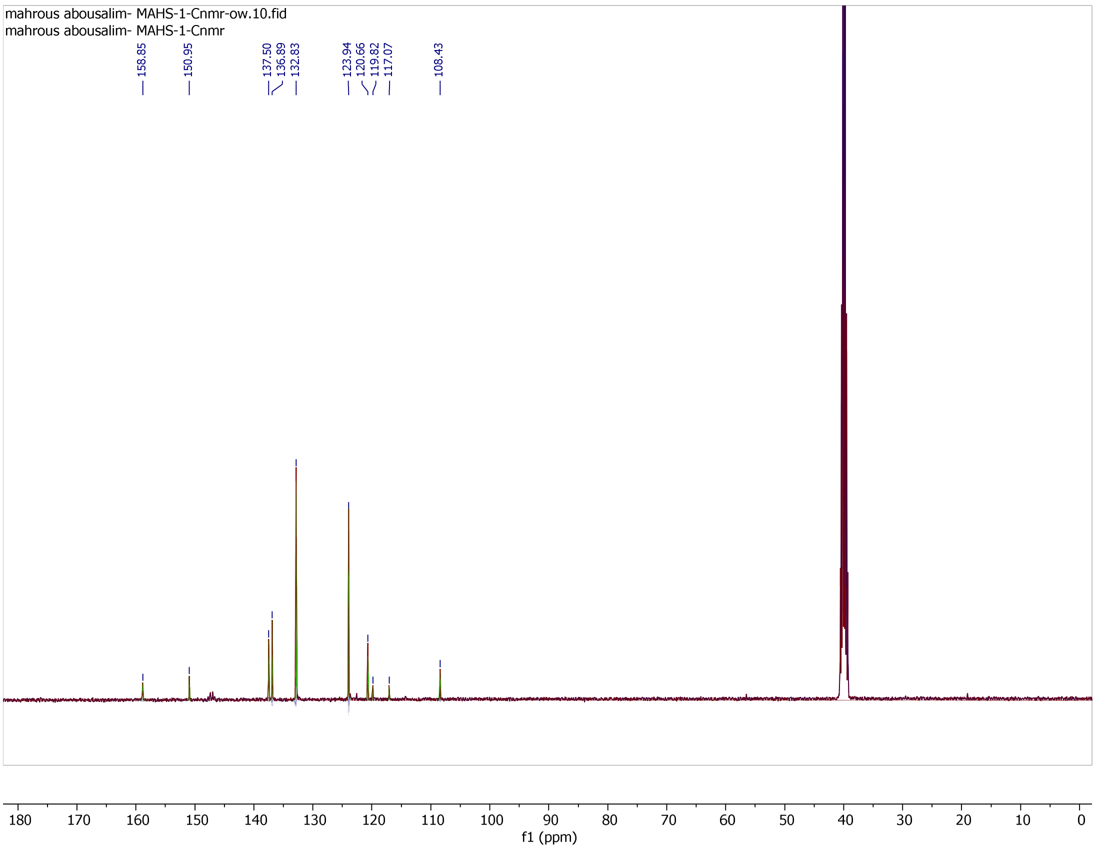

Figure S30. ^{13}C NMR of **8b**.

1.18 8c

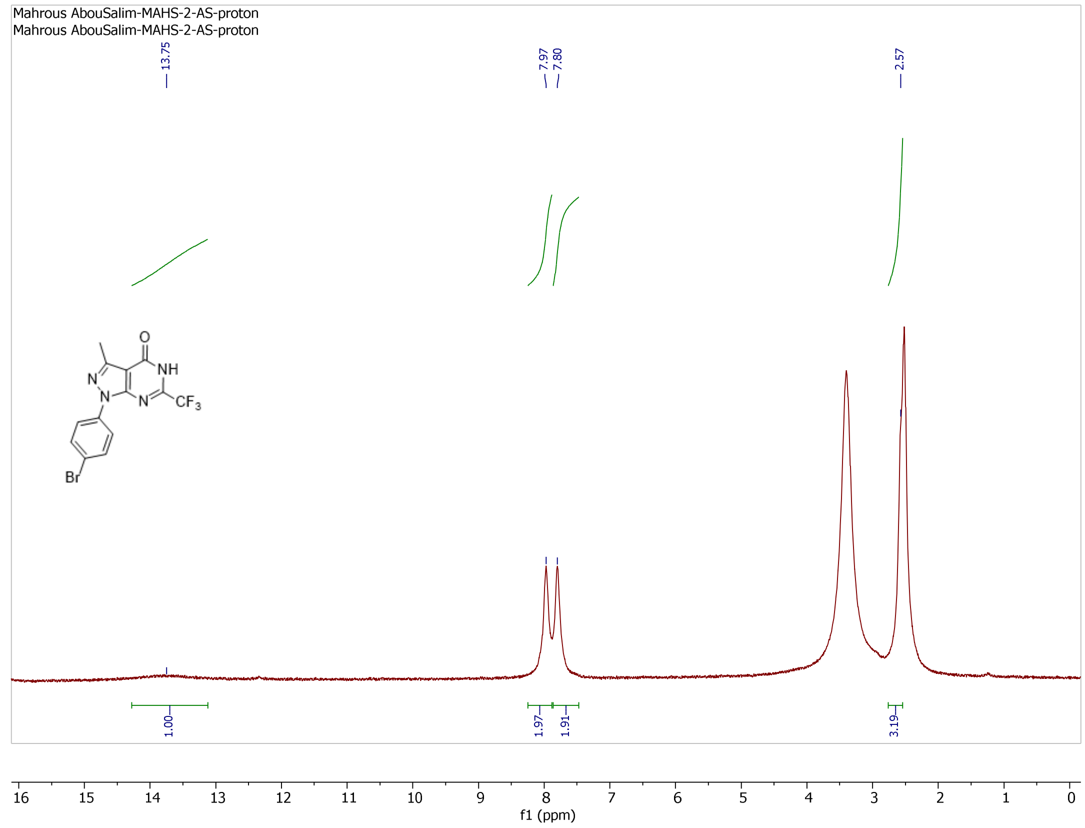

Figure S31. ^{1}H NMR of **8c**.

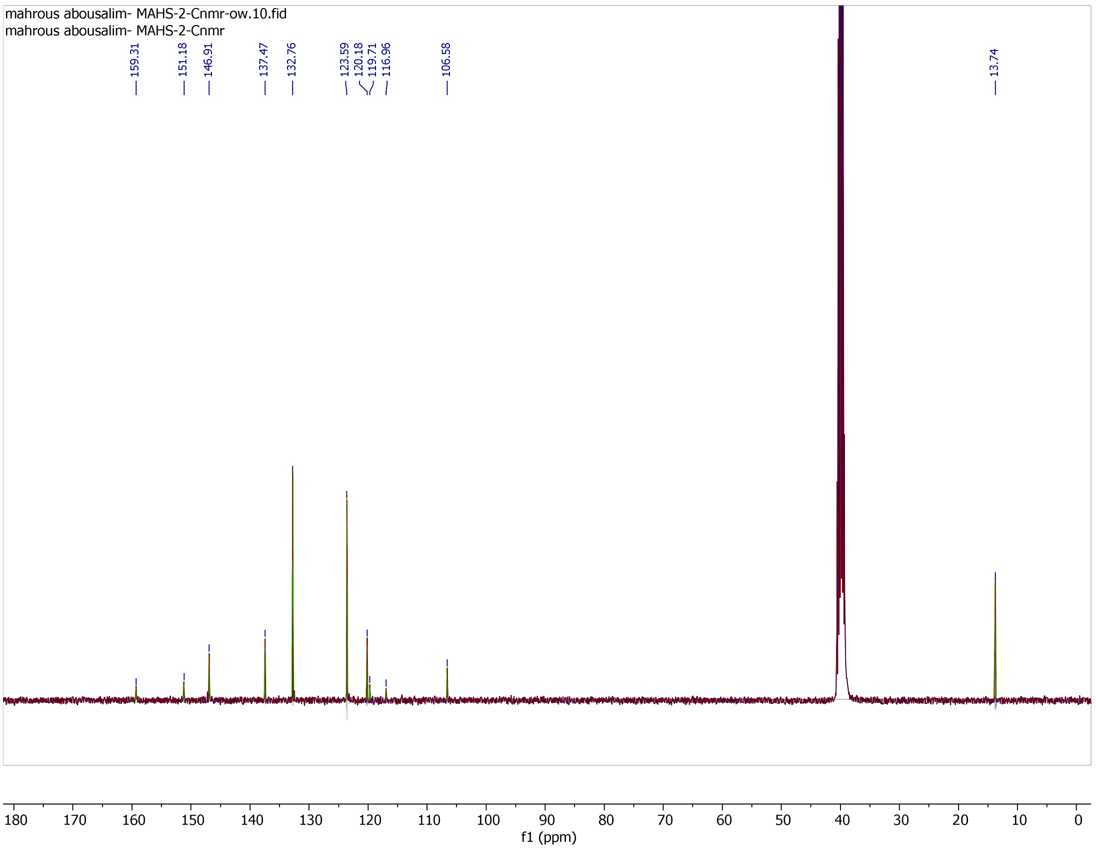

Figure S32. ^{13}C NMR of **8c**.

1.19 9a

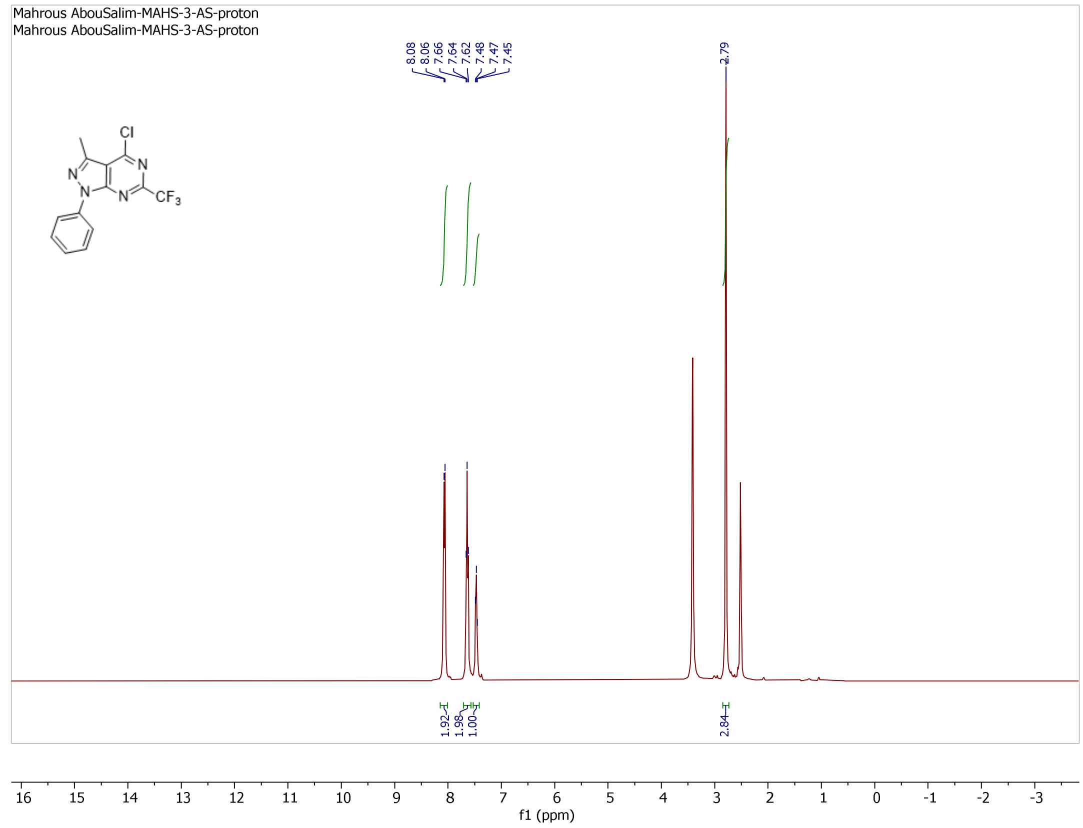

Figure S33. ^{1}H NMR of **9a**.

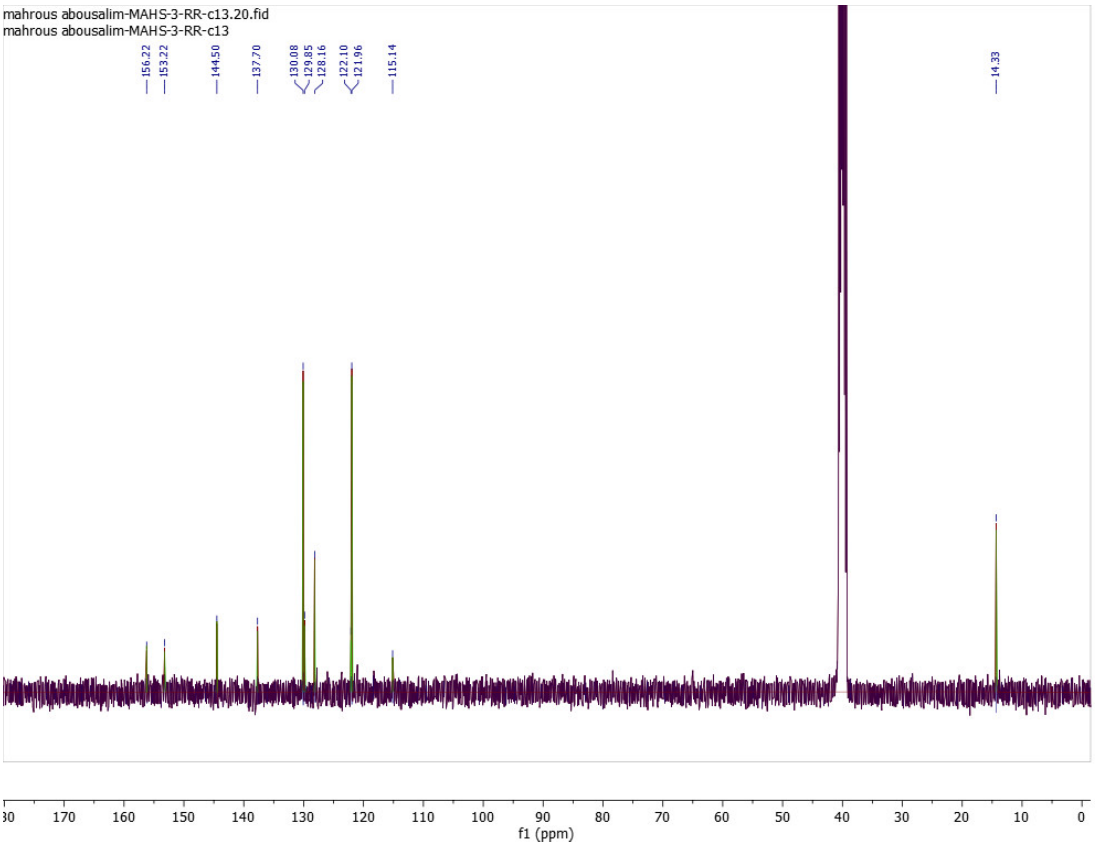

Figure S34. ^{13}C NMR of **9a**.

1.20 9b

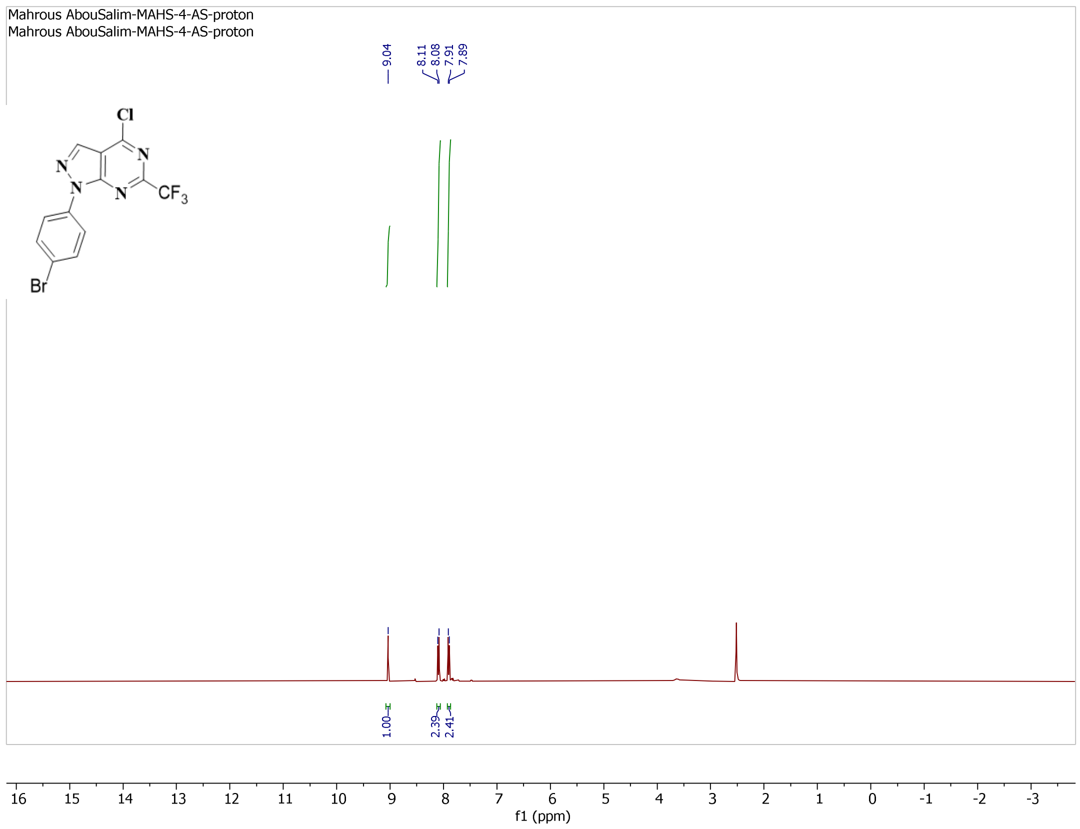

Figure S35. ^{1}H NMR of **9b**.

1.21 9c

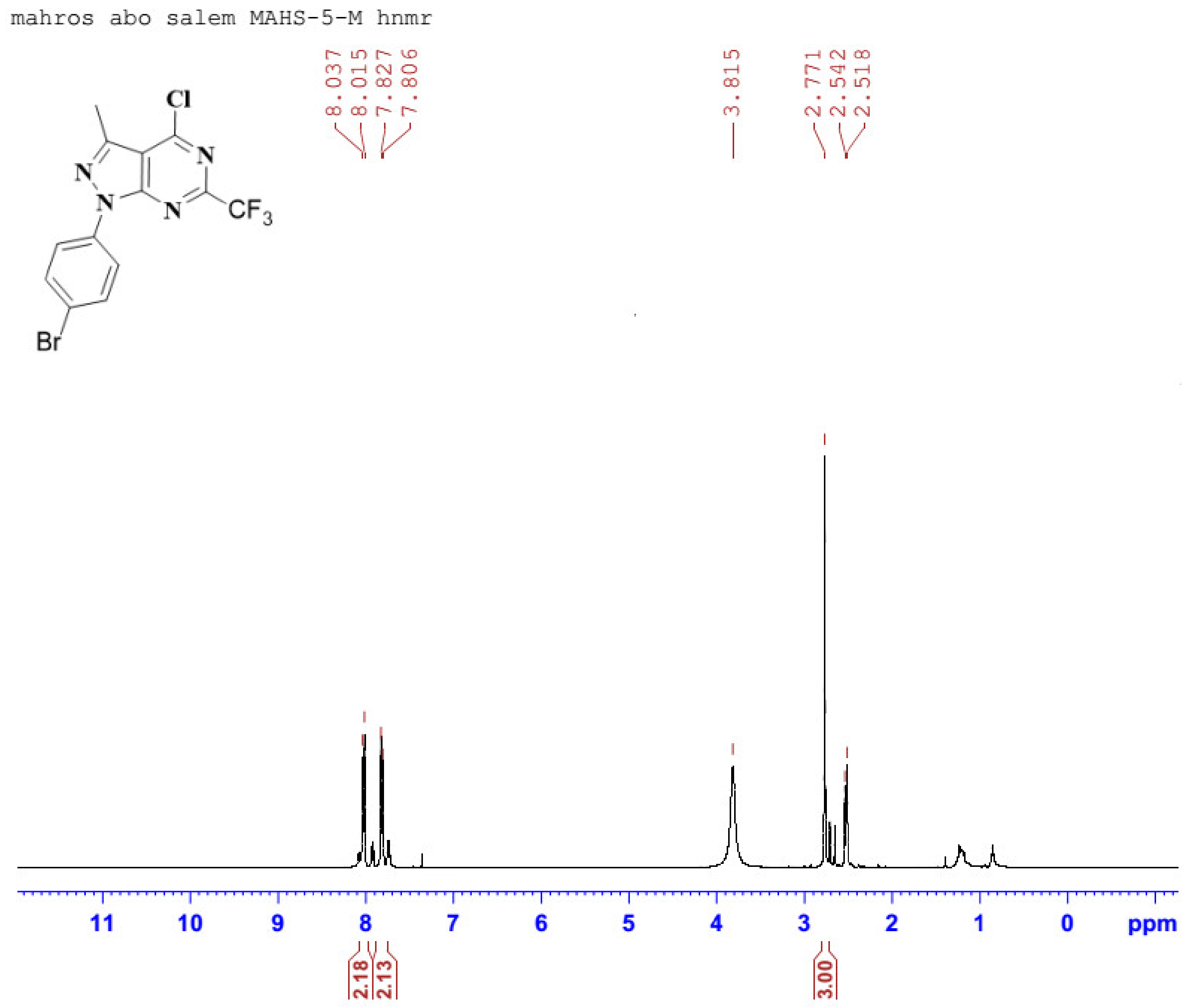

Figure S36. ^{1}H NMR of **9c**.

1.22 11

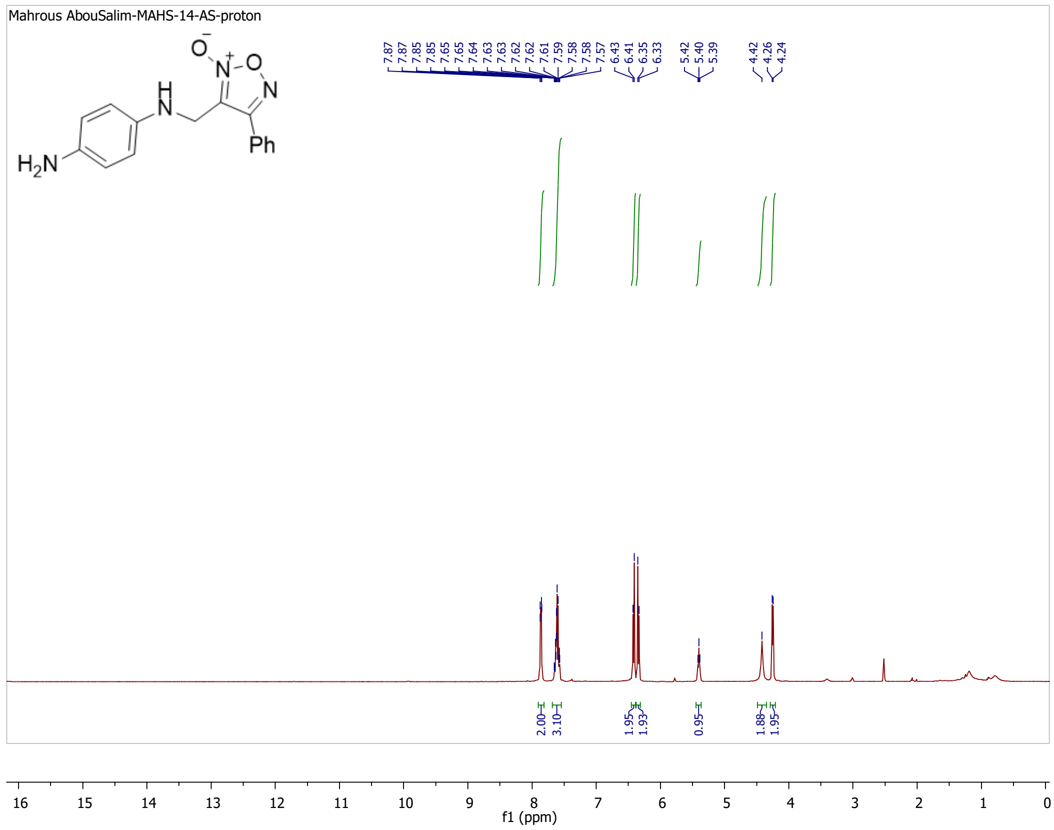

Figure S37. ¹H NMR of **11**.

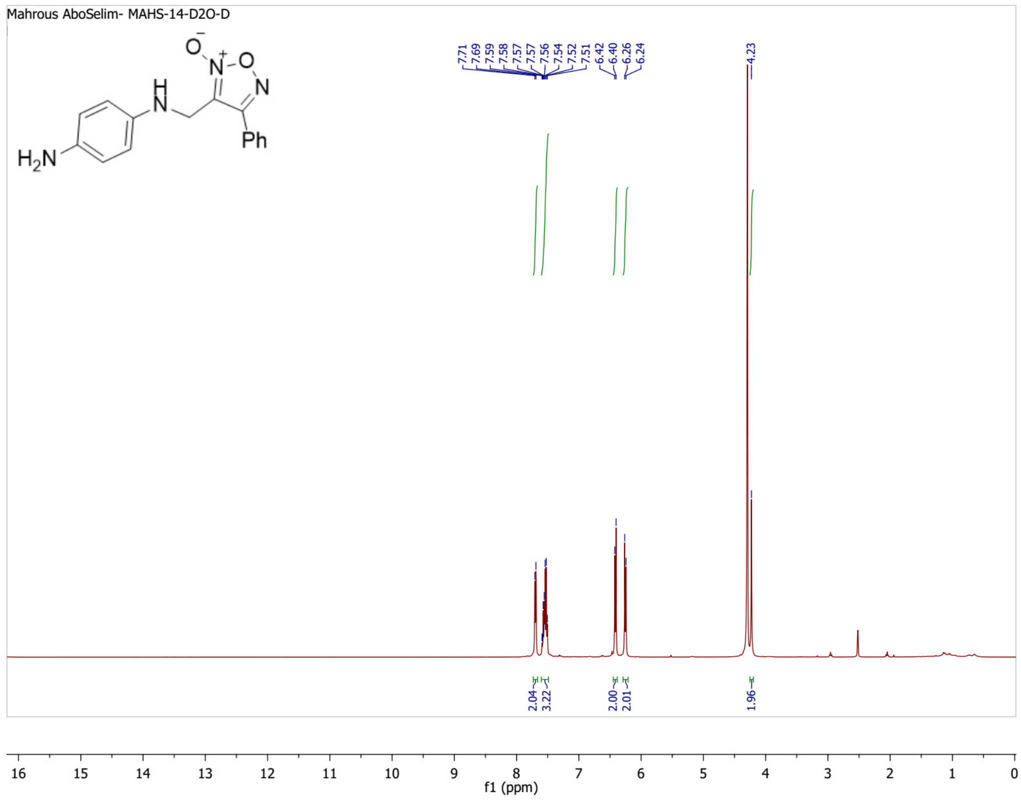

Figure S38. ¹H NMR (D_2O) of **11**.

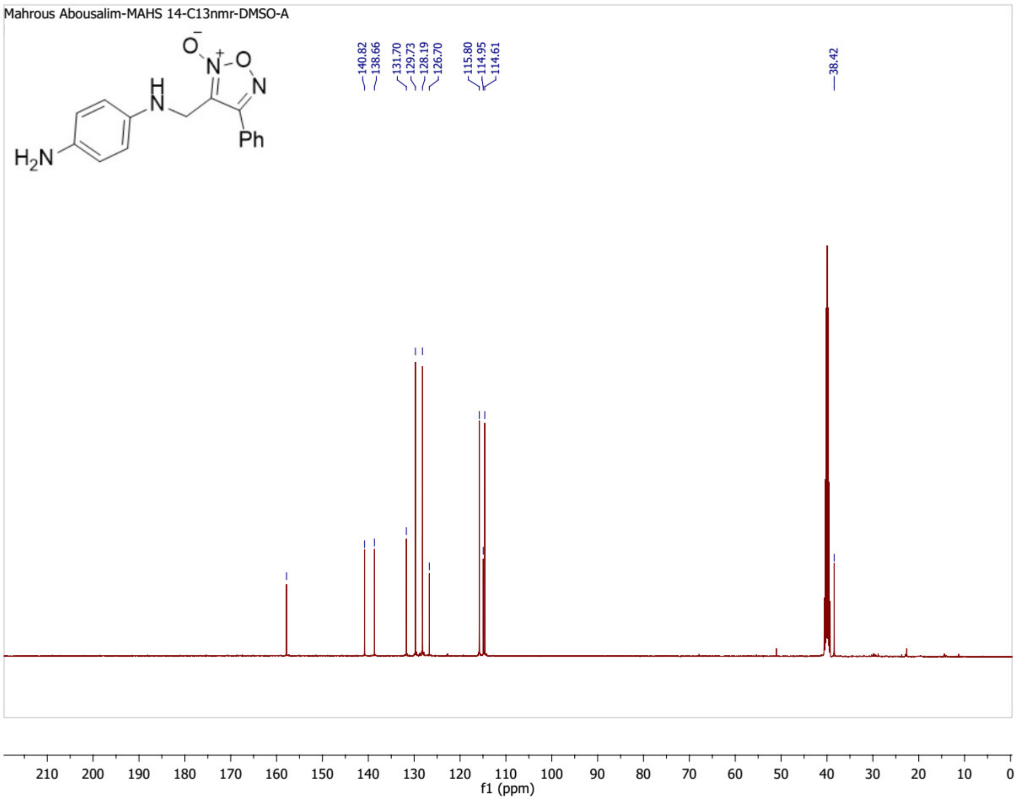

Figure S39. ^{13}C NMR of **11**.

1.23 12a

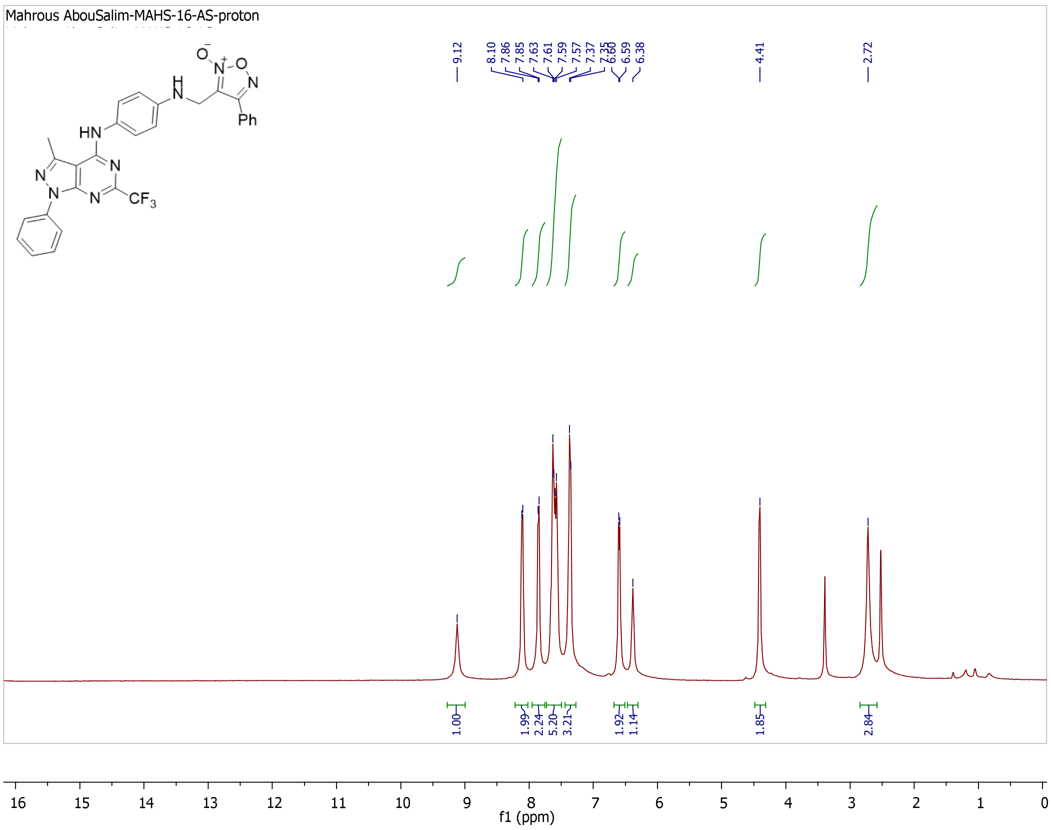

Figure S40. ^{1}H NMR of **12a**.

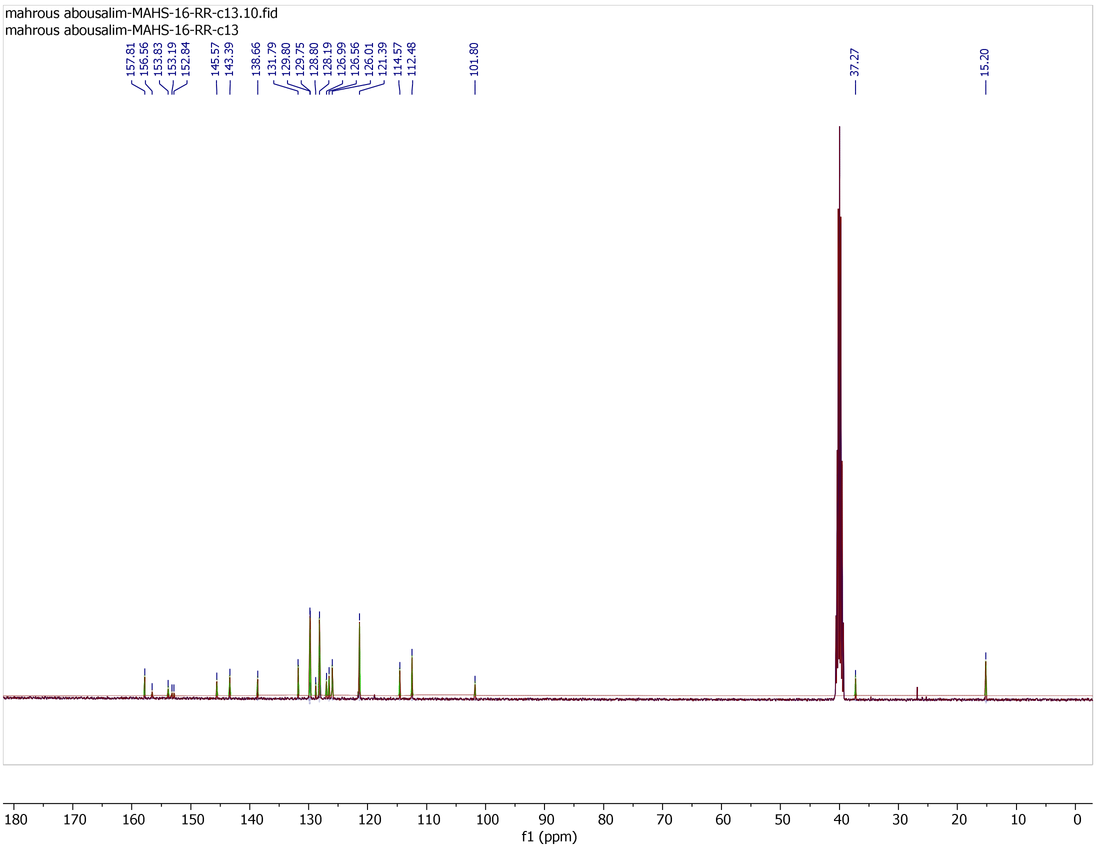

Figure S41. ^{13}C NMR of **12a**.

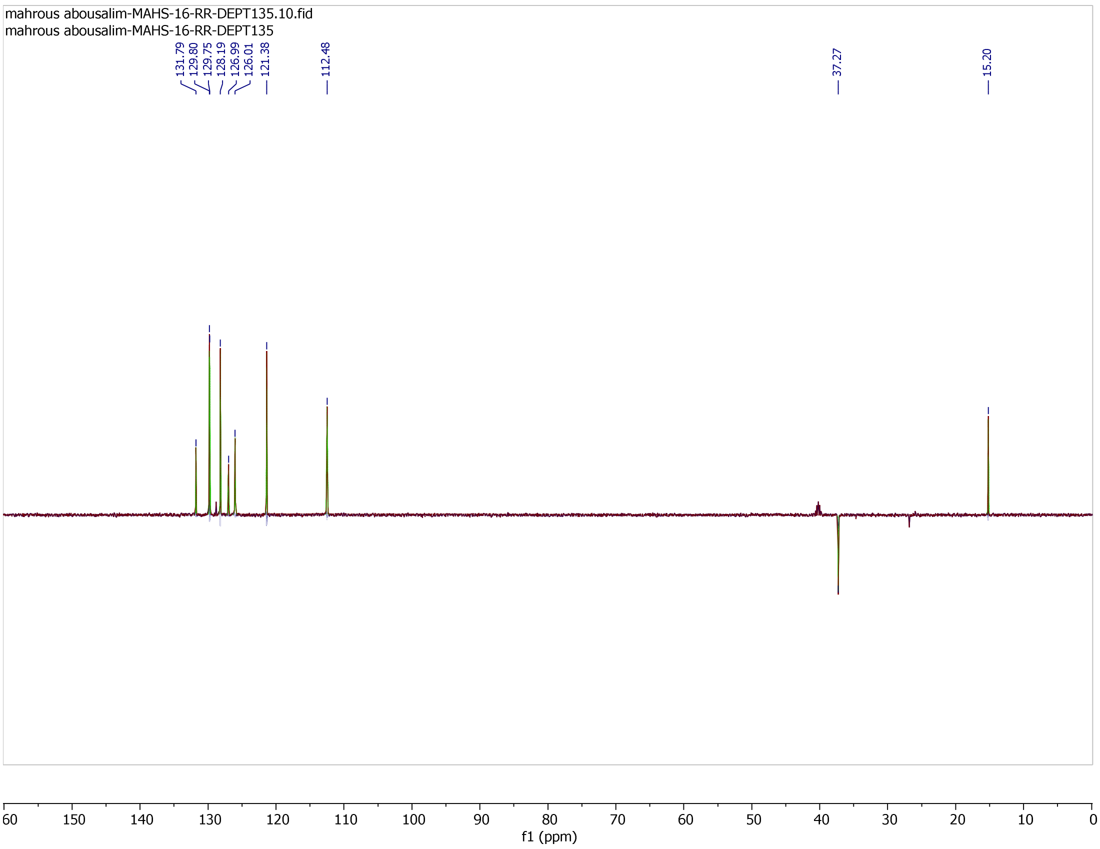

Figure S42. DEPT135 ^{13}C NMR of **12a**.

1.24 12b

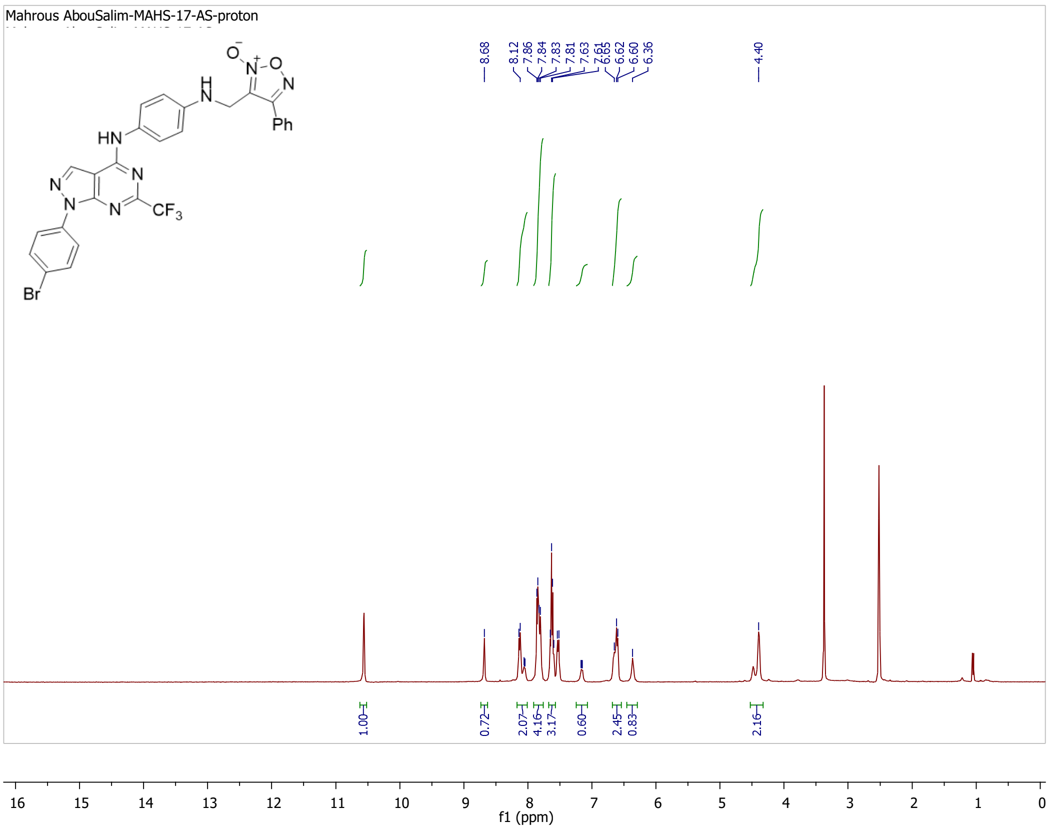

Figure S43. ^{1}H NMR of **12b**.

1.25 12c

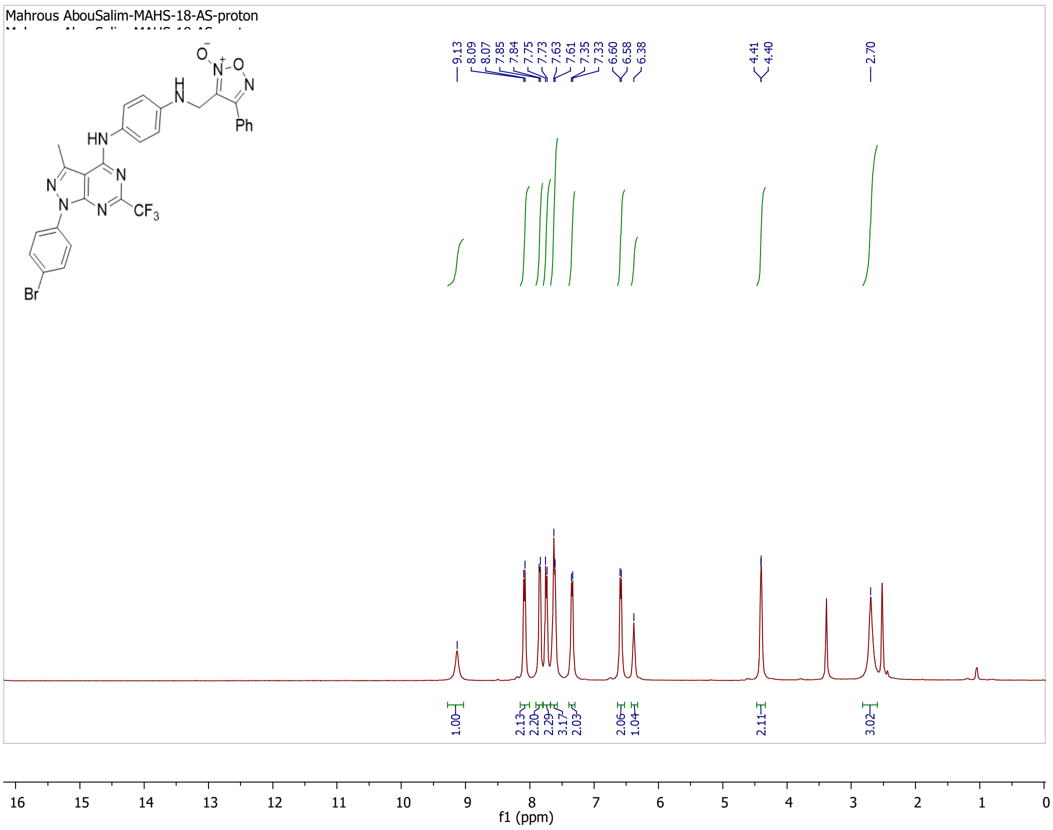

Figure S44. ^{1}H NMR of **12c.**

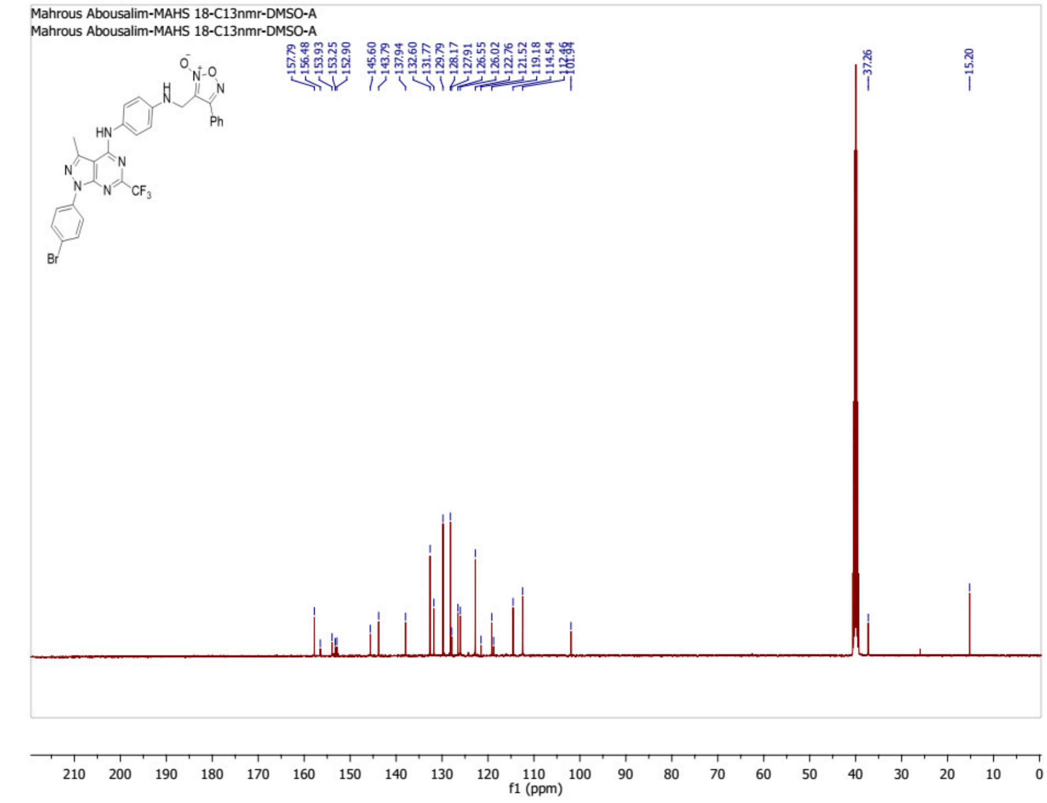

Figure ***S45*****.** ^{13}C NMR of ***12c***.

1.26 14a

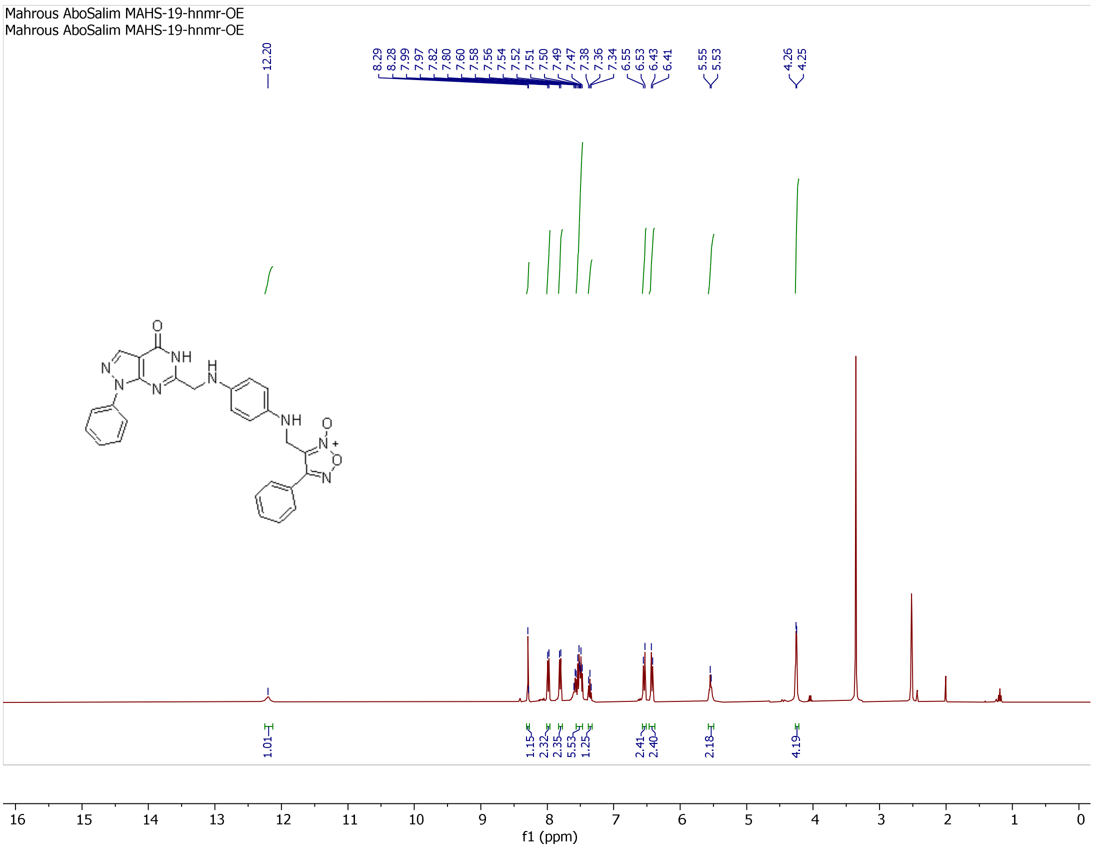

Figure S46. ^{1}H NMR of **14a**.

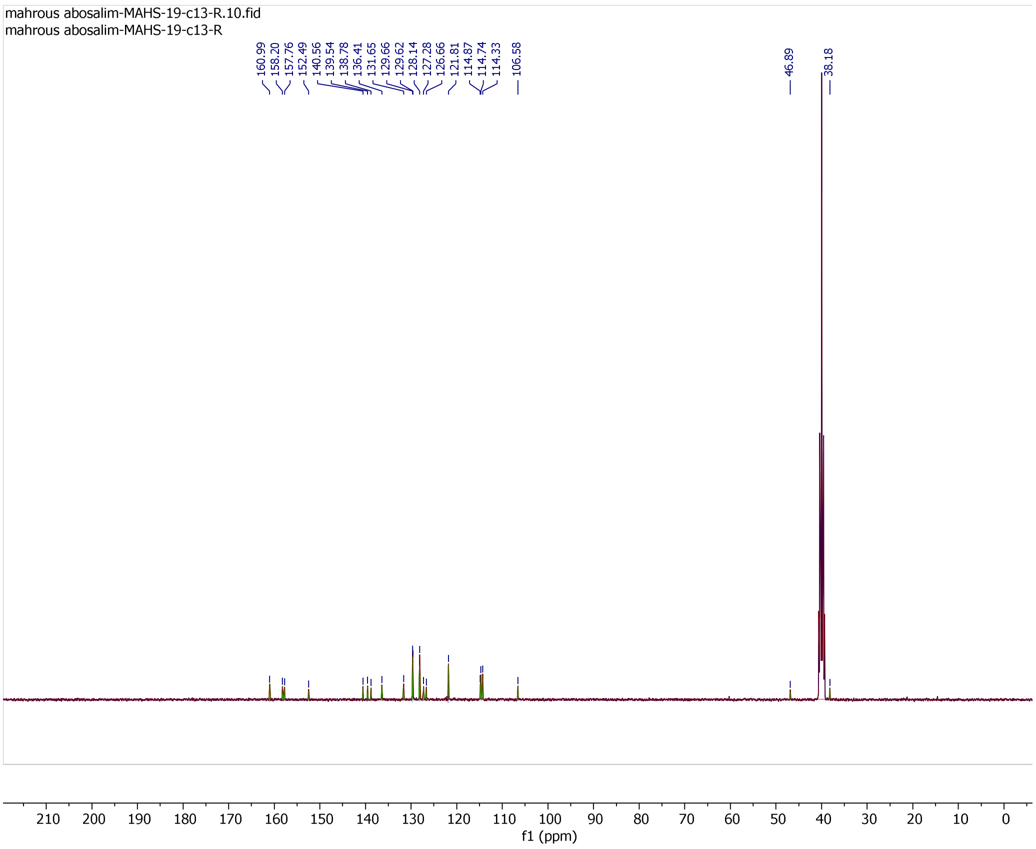

Figure S47. 13C NMR of ***14a***.

1.27 14b

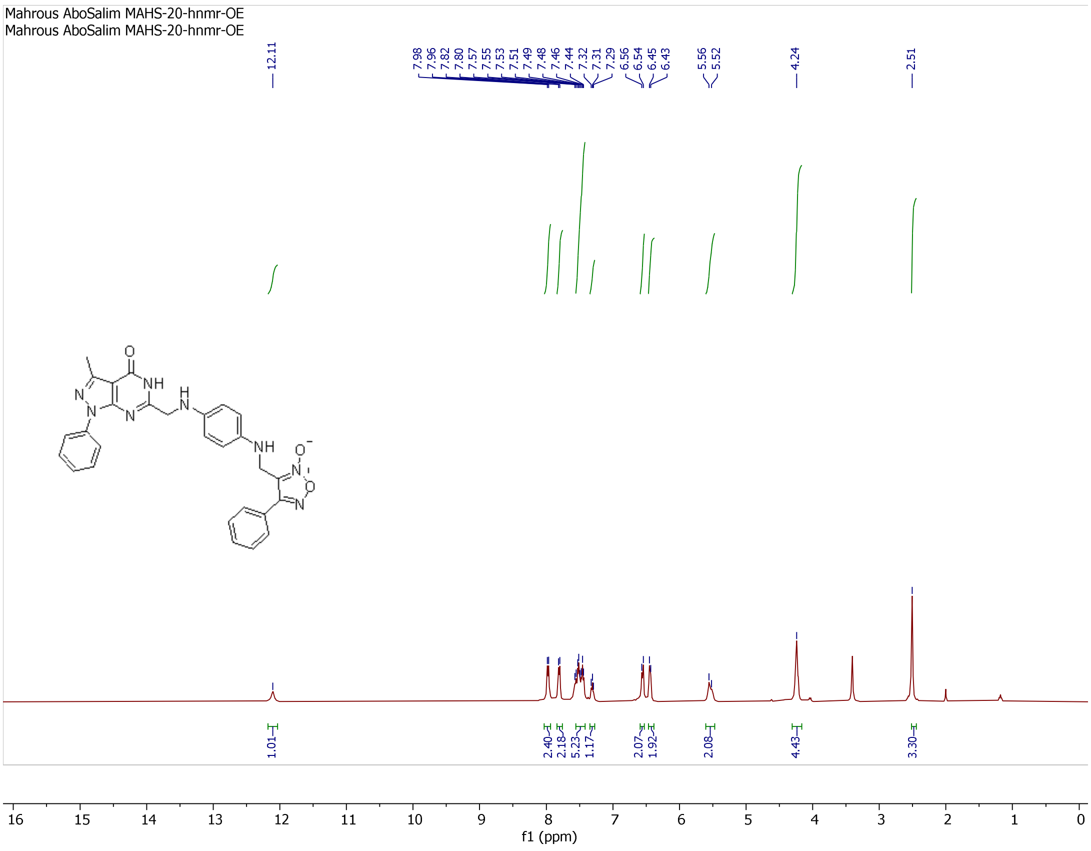

Figure S48. 1H NMR of **14b**.

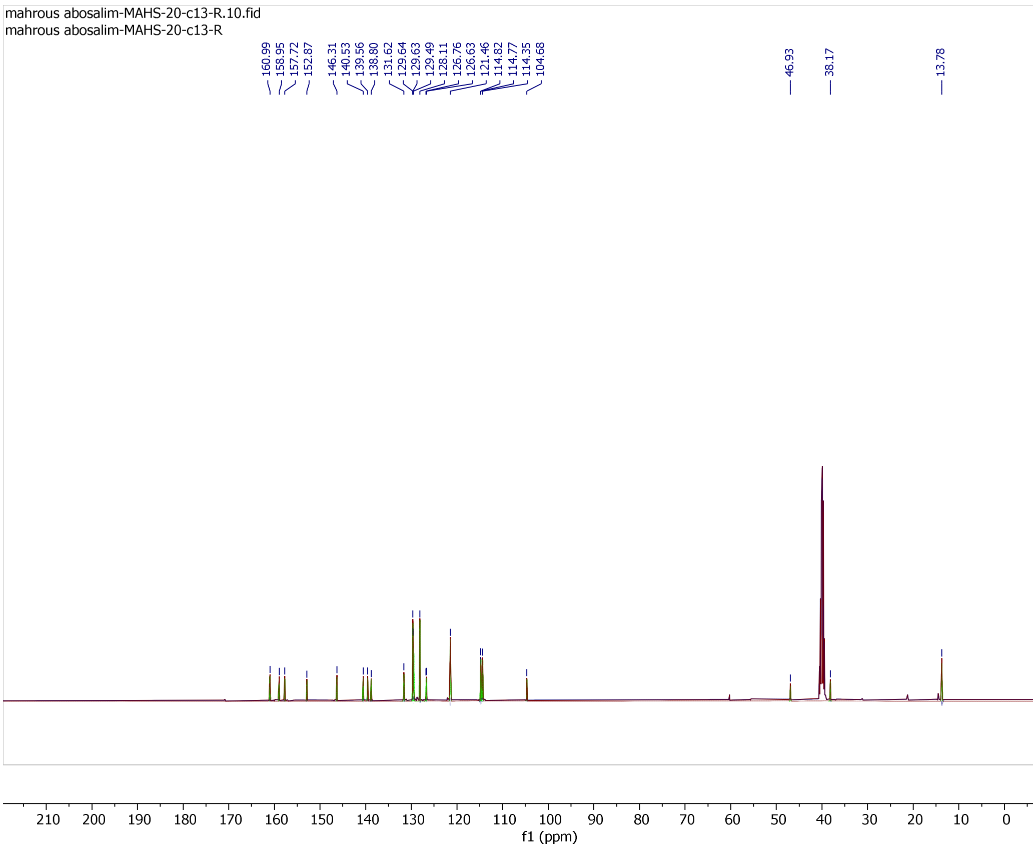

Figure S49. ^{13}C NMR of **14b**.

1.28 14c

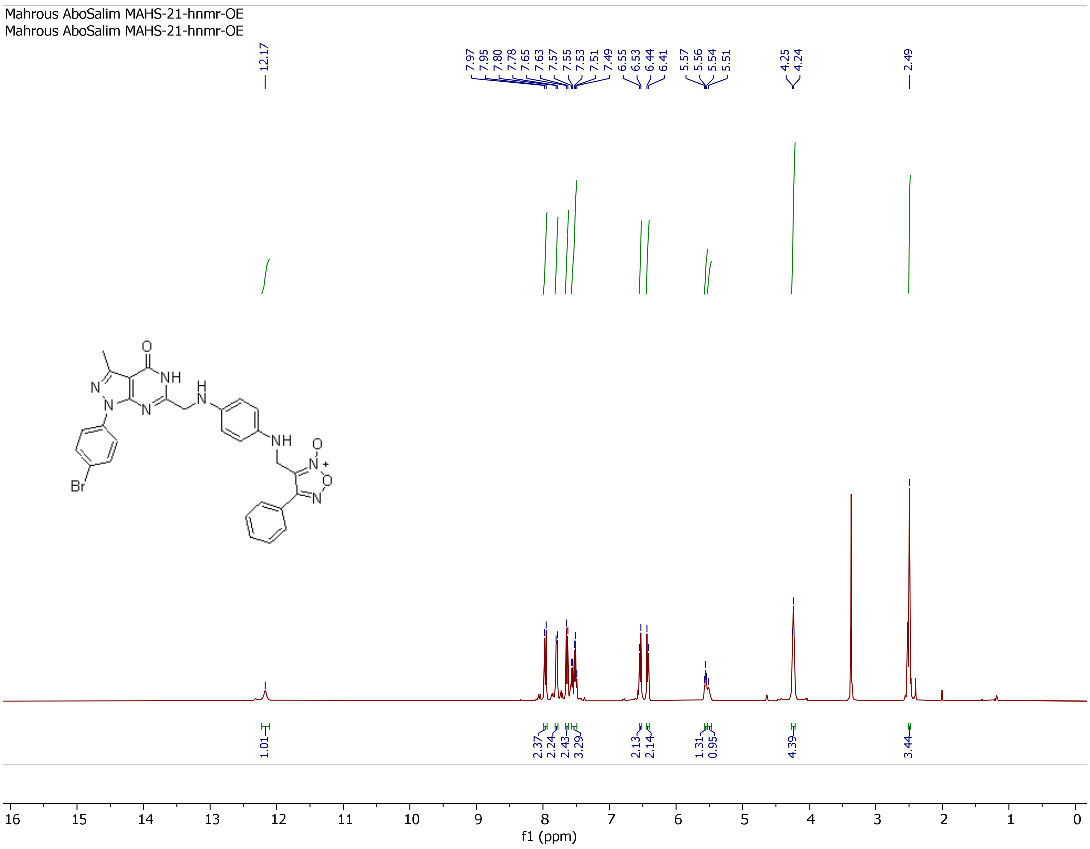

Figure S50. ^{1}H NMR of **14c**.

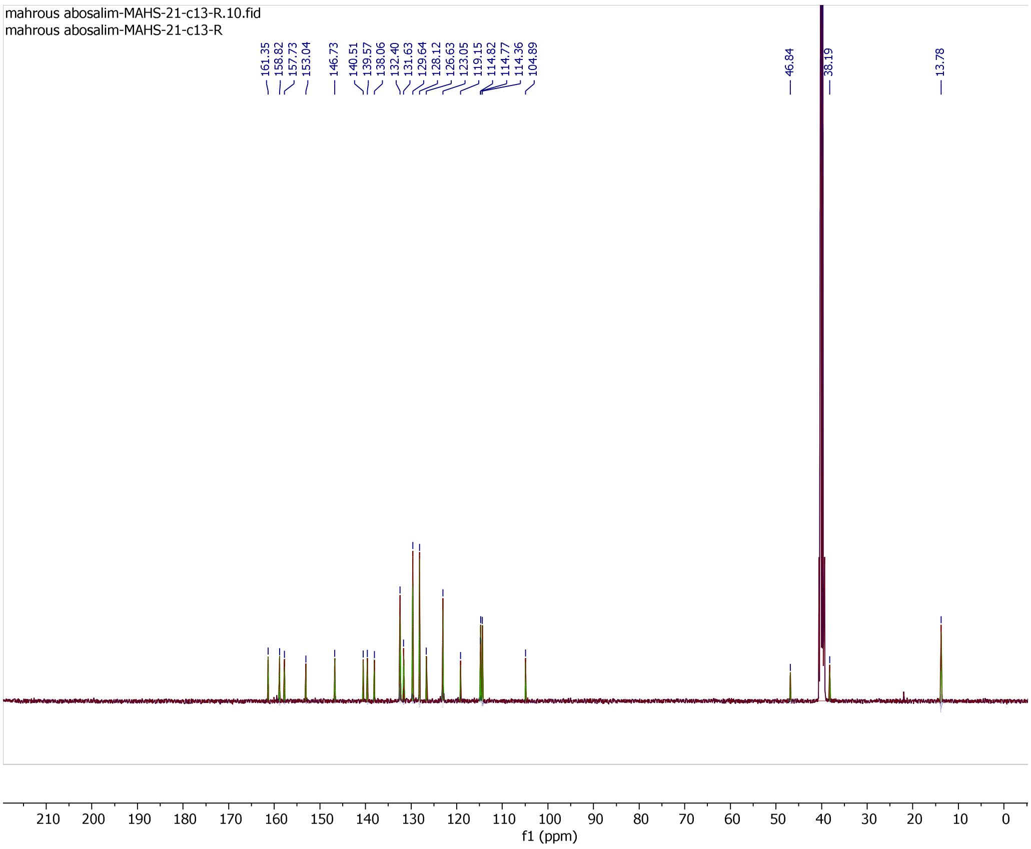

Figure *S51.* ^{13}C NMR of ***14c***.

1.29 15a

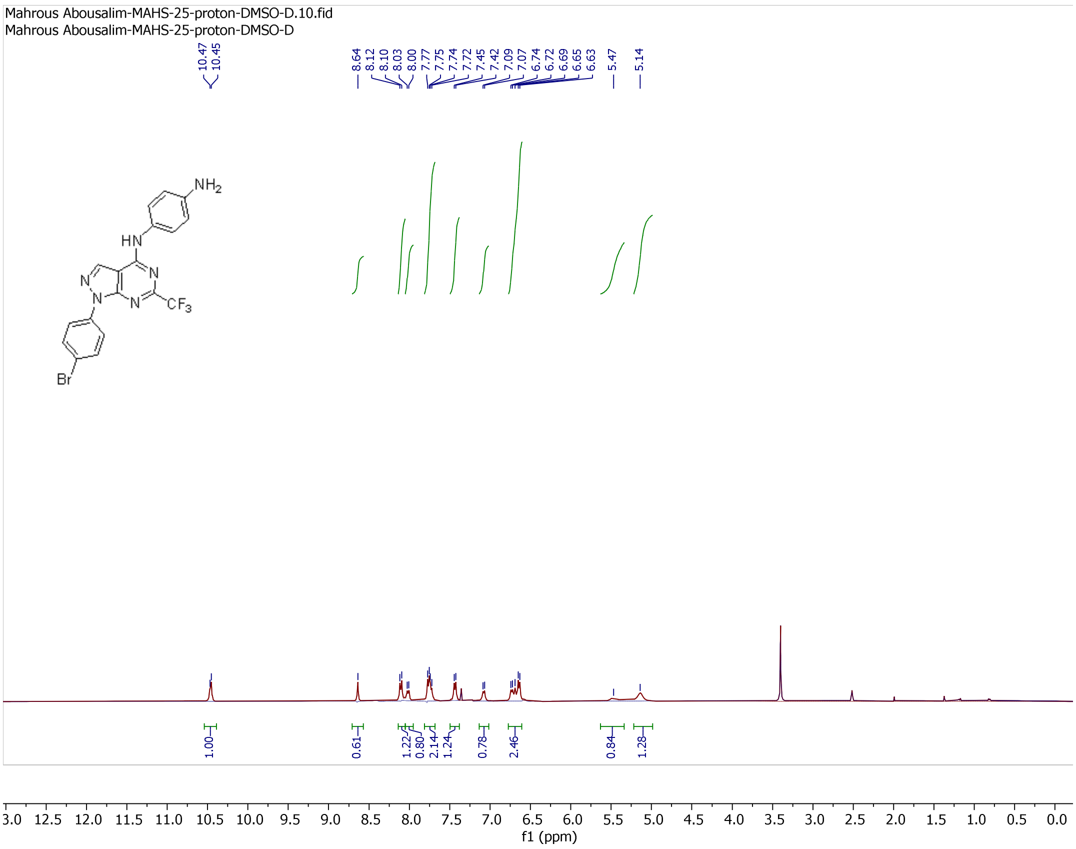

Figure S52. ^{1}H NMR of **15a**.

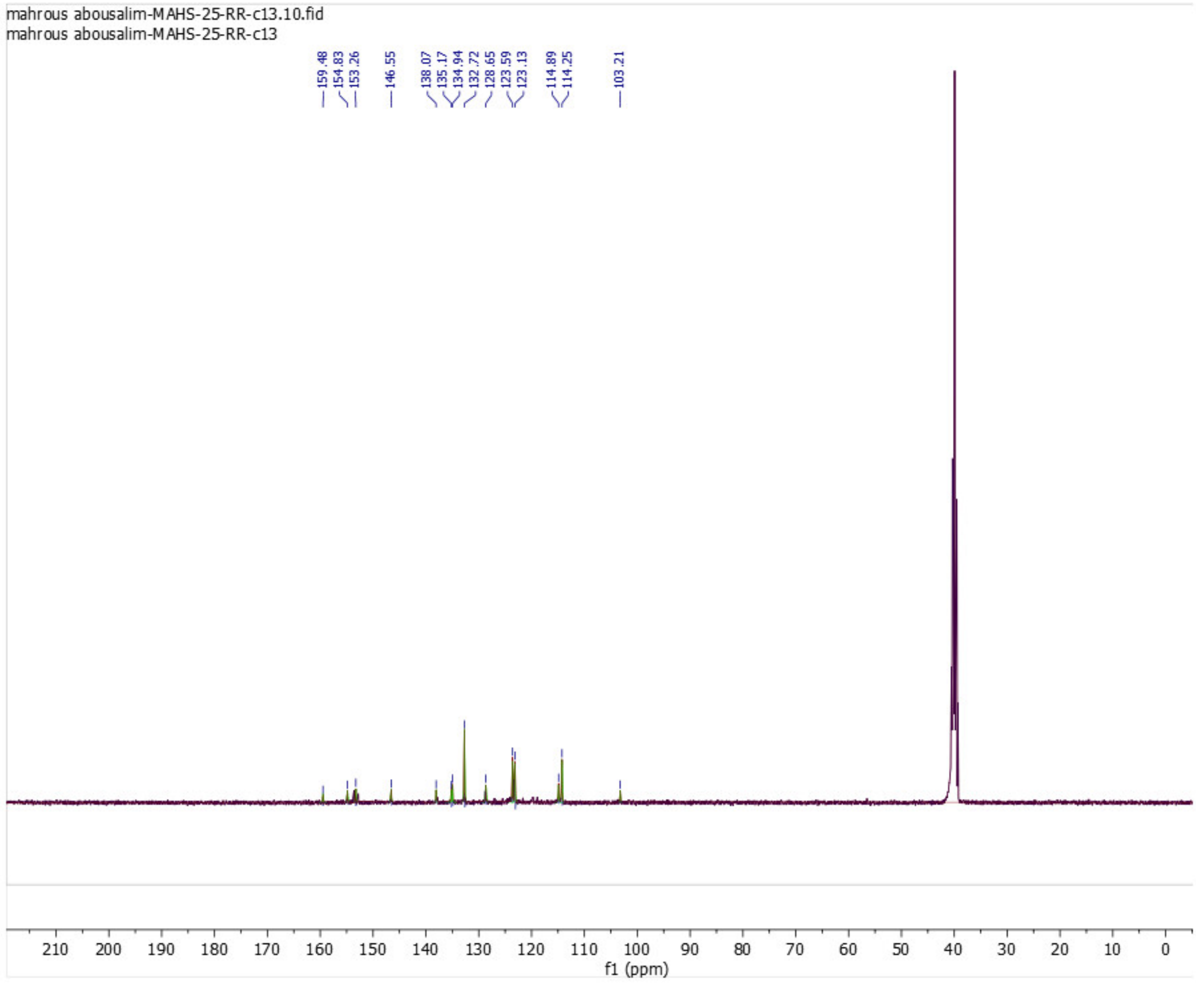

Figure S53. ^{13}C NMR of **15a**.

1.30 15b

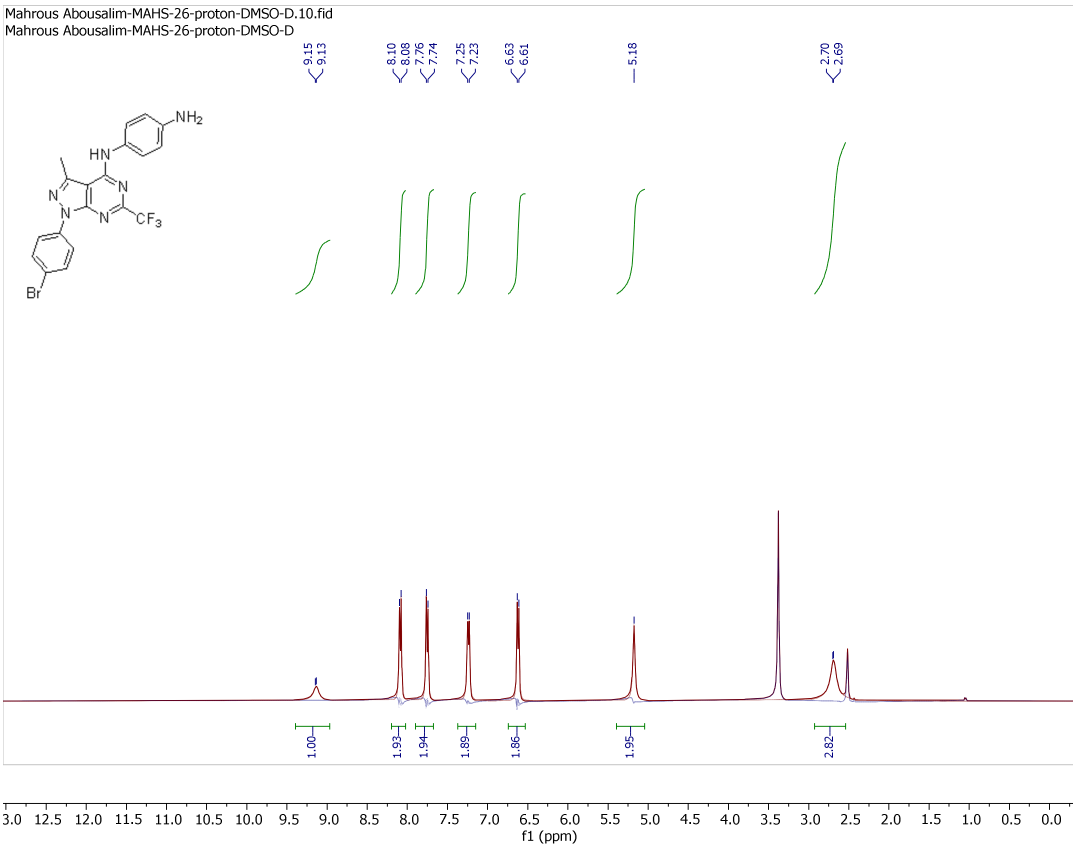

Figure S54. ^{1}H NMR of **15b**.

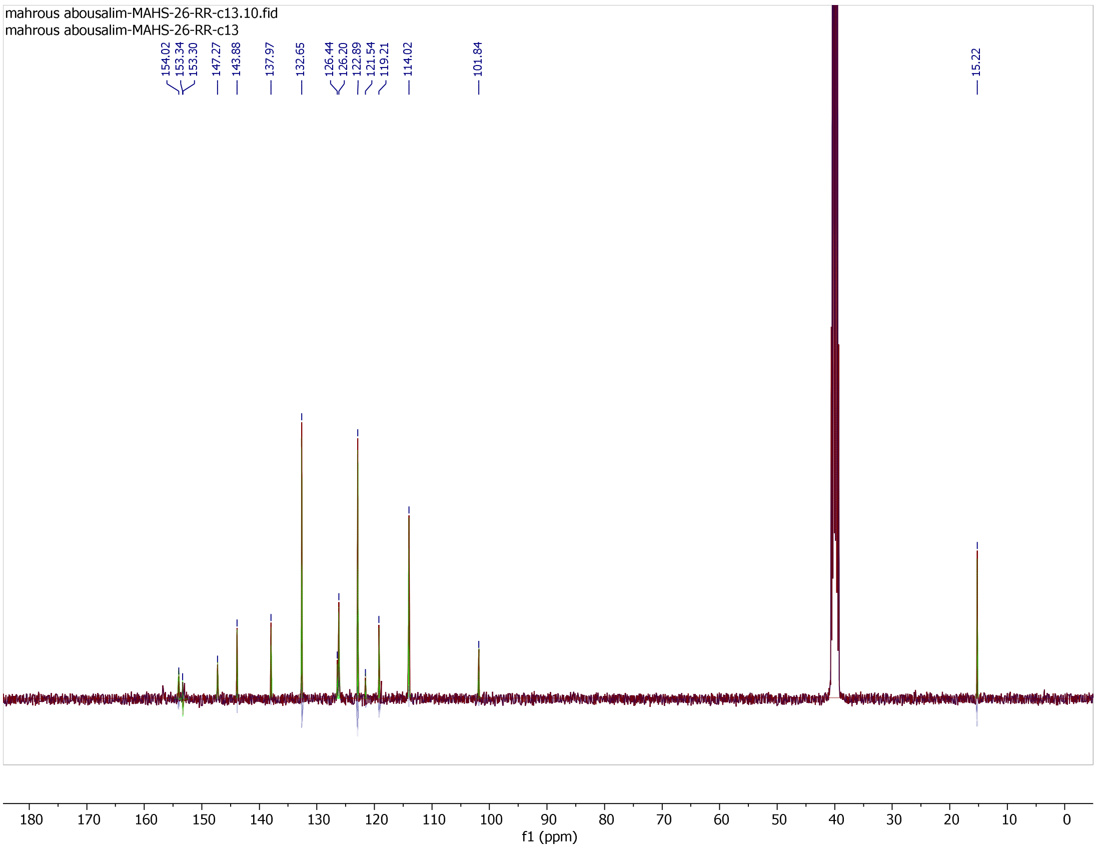

Figure S55. ^{13}C NMR of **15b**.

2 NCI-60 cell line screening

Table S1. NCI-60 cell line growth percentage screening results for target compounds **6a–h** and **12a–c**.

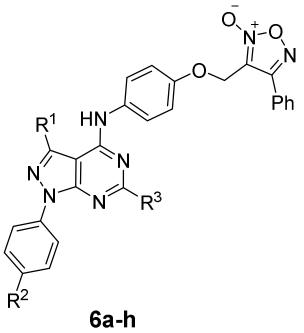

6a-h

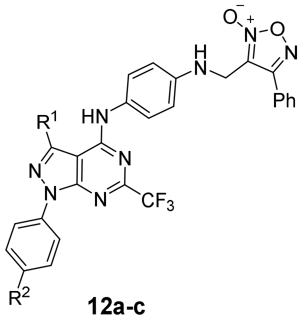

12a-c

Panel name	Cell name	6a	6b	6c	6d	6e	6f	6g	6h	12a	12b	12c
Leukemia	CCRF-CEM	91.04	102.25	97.37	112.57	107.88	104.28	98.11	103.16	59.23	48.49	60.4
	HL-60(TB)	94.67	122.25	118.81	111.45	96.09	100.13	115.37	70.79	77.18	79.82	83.19
	K-562	88	111.46	90.21	99.35	98.54	91.14	103.09	83.62	39.8	22.77	51.35
	MOLT-4	87.43	117.37	92.87	110.12	103.05	91.55	97.69	87.63	56.39	46.97	60.31
	RPMI-8226	104.16	114.03	93.99	106.01	111.23	115.2	108.22	90.59	83.41	73.8	91.13
	SR	85.21	89.14	73.92	102.41	97.95	87.08	120.7	72	58.67	43.47	55.48
Non-Small Cell Lung Cancer	A549/ATCC	96	100.55	99.3	103.09	93.53	94.86	97.27	96.4	76.64	64.44	82.38
	EKVX	98.92	93.09	90.37	101.7	96.35	91.4	83.47	92.26	73.16	65.4	71.11
	HOP-62	107.4	102.98	105.46	106.91	104.86	110.13	94.01	103.88	93.1	77.94	68.33
	HOP-92	114.93	91.42	91.18	103.5	108.9	111.33	86.53	94.56	90.13	68.36	82.26
	NCI-H226	111.11	94.74	97.32	101.32	104.26	107.9	89.92	108.63	85.58	76.99	74.96
	NCI-H23	104.58	101.56	98.12	102.1	100.58	95.84	94.95	96.57	96.74	72.37	91.64
	NCI-H322M	108.65	99.46	90.96	99.86	97.89	90.8	94.49	94.39	77.61	74.07	85.06
	NCI-H460	73.59	110.9	109.35	111.7	110.94	103.18	100.15	100.25	52.95	35.58	46.57
	NCI-H522	99.18	96.28	84.78	104.58	96.1	92.48	92.02	84.04	91.36	72.57	83.51
Colon Cancer	COLO 205	104.07	114.17	116.19	106.94	104.65	118.82	116.74	94.42	73.59	74.58	73.25
	HCC-2998	106.2	108.15	105.91	107.21	106.26	104.05	104.45	105.94	85.58	75.1	96.07
	HCT-116	92.96	99.24	81.85	103.76	92.74	97.47	90.71	71.46	41.11	46.67	31.88
	HCT-15	54.21	96.4	91.51	103.97	94.88	98.14	97.15	78.98	41.95	38.07	34.6
	HT29	96.01	105.66	93.24	119.1	94.52	97.26	101.19	90.03	61.53	43.9	70.41
	KM12	102.78	101.83	99.82	103.66	103.18	101.14	98.6	97.69	56.73	41.24	74.26
	SW-620	105.91	120.29	106.71	118.65	114.8	108.63	106.99	101.06	85.99	84.76	79.72
CNS Cancer	SF-268	103.42	102.96	96.49	105.79	100.82	99.92	104.45	94.7	68.61	64.95	73.2
	SF-295	98.39	97.09	99.05	107.96	100.94	102.15	87.58	105.37	76.59	54.52	57.45
	SF-539	95.73	101.85	96.18	99.82	98.37	94.25	90.41	96.08	78.06	68.29	59.37
	SNB-19	104.92	99.29	100.81	99.6	103.12	91.49	99.19	94.47	88	78.94	84.13
	U251	107.44	103.53	97.24	105.73	101.51	99	102.06	96.36	69.93	73.17	54.13
Melanoma	LOX IMVI	94.26	97.58	100.63	96.64	110.64	104.53	94.96	98.91	57.95	27.94	48.54
	MALME-3M	90.01	99.35	98.19	101.49	95.27	105.63	91.61	96.03	91.04	93.53	77.7

Panel name	Cell name	6a	6b	6c	6d	6e	6f	6g	6h	12a	12b	12c
	M14	103.71	102.97	90.27	96.17	101.79	94.24	94	89.47	68.7	68.18	71.9
	MDA-MB-435	114.31	99.7	93.4	104.96	94.48	94.48	101.38	96.74	78.65	72.77	86.57
	SK-MEL-2	104.56	109.32	102.54	120.98	99.16	113.67	101.23	96.89	105.94	99.8	103.84
	SK-MEL-28	104.38	100.93	101.78	101.92	99.32	103.33	99.45	101.07	92.4	89.22	89.51
	SK-MEL-5	101.72	102.79	90.37	97.99	95.87	99.66	96.52	91.48	70.32	86	61.69
	UACC-257	98.86	101.17	104.84	109.22	103.6	101.48	97.09	99.8	102.82	102.46	95.7
	UACC-62	102.94	88.41	81.38	89.61	89.07	84.79	80.3	80.24	64.87	64.92	62.51
Ovarian Cancer	IGROV1	95.05	101.42	97.12	106.25	105.56	101.06	88.15	99.02	77.88	60.07	59.62
	OVCAR-3	95.93	108.51	105.27	113.83	116.58	113.86	111.9	104.26	105.05	91.97	91.56
	OVCAR-4	130.15	101.92	90.57	102.62	100.33	99.53	95.72	96.99	70.76	78.52	81.28
	OVCAR-5	110.22	105.87	103.77	100.77	98.76	99.29	99.26	102.49	93.72	77.15	89.54
	OVCAR-8	105.02	105.16	101.28	107.45	104.85	106.4	93.92	102.21	89.7	72.77	75.76
	NCI/ADR-RES	102.17	103.18	91.74	102.3	106.51	105.74	93.83	101.88	83	66.38	88.75
	SK-OV-3	101.8	109.92	104.32	109.35	104.43	113.29	96.19	108.54	96.87	88.84	82.71
Renal Cancer	786-0	115.3	95.78	106.76	107.35	96.13	82.25	121.94	106.67	92.39	67.15	88.11
	A498	103	101.84	86.35	111.89	93.98	87.19	106.36	109.59	94.43	97.4	116.68
	ACHN	104.77	104.8	97.31	95.53	103.23	101.44	88.01	98.67	77.73	56.91	68.07
	CAKI-1	99.36	87.57	85.97	101.17	82.14	81.89	84.89	83.77	53.03	31.1	55.05
	RXF 393	98.39	110.52	112.21	118.64	118.82	121.41	131.01	135.54	86.8	51.18	89.2
	SN12C	97.31	100.82	87.9	98.39	96.43	94.79	91.26	96.43	77.71	57.98	76.16
	TK-10	103.11	94.81	110.09	125.68	106.77	97.66	122.57	114.06	94.39	86.83	109.09
	UO-31	94.17	95.03	77.03	88.52	84.68	82.73	81.52	74.04	57.52	43.42	50.26
Prostate Cancer	PC-3	98.13	103.14	83.52	99.01	96.95	102.74	90.94	67.39	45.65	48.22	46.96
	DU-145	107.32	104.51	105.81	111.53	107.32	103.93	109.58	102.44	82.25	70.76	84.78
Breast Cancer	MCF7	96.12	92.38	83.01	95.41	87.88	85.3	89.04	87.38	58.52	38.79	68.23
	MDA-MB-231/ATCC	97.55	106	95.65	101.12	107.57	106.3	84.9	99.97	84.4	69.7	65.9
	HS 578T	98.73	109.67	97.53	101.62	109.81	93.64	93.41	94.75	77.88	77.61	83.15
	BT-549	95.38	94.95	101.3	102.15	95.83	94.5	104.23	91.1	103.19	97.5	97.42
	T-47D	96	105.6	71.97	104.36	99.88	97.15	98.47	81.39	80.45	68.83	85.62
	MDA-MB-468	106.81	94.26	97.08	107.05	105.66	111.1	97.42	116.06	75.83	71.95	84.2
**Mean**		100.06	102.34	96.2	104.91	101.07	99.74	98.42	95.43	76.8	66.83	74.78
**Delta**		45.85	14.77	24.23	16.39	18.93	17.85	18.12	28.04	37	44.06	42.9
**Range**		75.94	34.68	46.84	37.16	36.68	39.52	50.71	68.15	66.14	79.69	84.8

Table S2. NCI-60 cell line growth percentage screening results for compounds **11, 13a–c,** and **14a–c**.

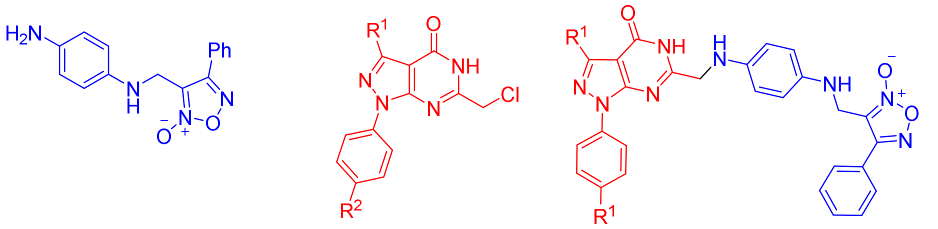

11 **13a-c** **14a-c**

Panel name	Cell name	11	13a	13b	13c	14a	14b	14c
Leukemia	CCRF-CEM	83.37	101.21	ND	83.65	57.91	78.76	105.22
	HL-60(TB)	95.51	108.73	33.07	ND	63.09	93.68	103.2
	K-562	99.1	87.53	19.16	75.57	49.77	75.8	110.66
	MOLT-4	91.79	103.59	79.63	ND	44.02	61.55	87.17
	RPMI-8226	97.48	82.53	ND	-53.91	61.94	78.99	111.75
	SR	103.87	87.13	39.76	ND	45.67	73.05	95.15
Non-Small Cell Lung Cancer	A549/ATCC	95.09	98.55	97.67	96.91	63.76	74.31	97.39
	EKVX	108.98	99.95	99.24	99.52	51.31	83.05	98.93
	HOP-62	135.92	125.45	98.34	98.54	104.53	109.59	116.09
	HOP-92	116.12	100.94	117.39	100.66	85.39	93.19	107.67
	NCI-H226	114.2	110.08	111.37	101.58	77.15	100.85	108.98
	NCI-H23	104.56	101.47	98.81	102.56	63.38	86.6	103.09
	NCI-H322M	102.53	101.08	102.74	104.14	80.21	88.99	104.06
	NCI-H460	101.49	101.49	102.13	108.94	64.19	89.95	106.97
	NCI-H522	93.47	102.7	89.79	85.46	60.75	80.86	90.77
Colon Cancer	COLO 205	120.75	113.67	110.35	102.99	101.27	116.22	114.51
	HCC-2998	117.73	105.37	107.9	105.26	84.43	110.82	112.32
	HCT-116	98.42	94.89	24.88	98.34	49.64	78.98	93.76
	HCT-15	104	99.88	90.57	103.06	71.19	80.57	97.31
	HT29	110.92	114.56	105.88	103.6	92.01	107.47	110.02
	KM12	103.2	100.52	53.31	83.63	73.35	94.46	105.52
	SW-620	114.98	101.71	85.06	109.62	92.79	109.92	111.22
CNS Cancer	SF-268	101.06	99.94	106.41	99.42	73.46	84.94	95.24
	SF-295	117.43	109.84	109.05	100.36	71.76	102.54	115.3
	SF-539	103.88	97.09	36.89	76.97	92.62	99.39	101.75
	SNB-19	109.54	96.35	89.43	97.13	94.01	100.52	101.91
	U251	101.65	93.65	93.92	99.72	76.08	94.56	99.13
el an o m	LOX IMVI	107.14	102.65	100.54	98.49	85.52	97.63	101.98

Panel name	Cell name	11	13a	13b	13c	14a	14b	14c
	MALME-3M	100	-38.51	2.09	-9.8	63.61	74.09	92.51
	M14	102.33	110.15	102.62	105.69	80	105.91	106.53
	MDA-MB-435	90.2	95.67	98.99	101.41	70.69	91.56	101.36
	SK-MEL-2	104.62	103.78	97.9	103.15	77.81	100.37	106.96
	SK-MEL-28	100.57	-89.82	104.62	99.87	77.17	99.23	102.96
	SK-MEL-5	111.85	106.69	109.98	102.63	85.29	108.26	103.56
	UACC-257	99.18	98.96	98.75	101.87	39.03	90.08	101.25
	UACC-62	95.53	96.17	97.9	100.63	60.63	98.47	105.22
Ovarian Cancer	IGROV1	88.73	96.21	96.16	95.09	52.51	64.36	85.58
	OVCAR-3	111.29	95.9	97.39	97.37	73.46	87.63	93.9
	OVCAR-4	117.84	100.13	90.89	115.85	94.45	101.91	127.06
	OVCAR-5	115.66	110.66	109.54	115.89	64.29	95.44	110.28
	OVCAR-8	131.45	114.21	112.48	102.24	108.67	117.17	111.71
	NCI/ADR-RES	104.44	103.77	102.43	101.76	79.36	92.4	101.27
	SK-OV-3	105.07	106.32	104.73	104.49	60.57	85.44	106.58
Renal Cancer	786-0	140.04	121.81	122.34	105.65	101.89	125.21	119.28
	A498	108.09	77.86	4.55	-88.76	85.95	97.94	104.38
	ACHN	104.06	96.68	90.25	94.79	70.42	88.76	96.91
	CAKI-1	108.4	97.61	99.62	96.32	55.06	67.06	90.2
	RXF 393	134.38	94.11	99.19	119.04	98.44	107.68	116.98
	SN12C	108.69	99.78	95.71	100.14	78.95	89.43	98.75
	TK-10	155.84	154.35	63.25	111.26	102.29	136.8	132.5
	UO-31	98.87	-20.87	-86.33	-90.14	55.94	72.36	83.36
Prostate Cancer	PC-3	95.12	104.61	102.15	96.35	63.34	77.68	96.61
	DU-145	111.76	98.62	83.61	110.05	74.63	90.74	106.83
Breast Cancer	MCF7	85.52	79.53	16.43	82.32	50.05	77	88.1
	MDA-MB-231/ATCC	103.56	101.02	88.41	94.07	72.55	82.67	96.45
	HS 578T	108.57	110.78	120.34	107.81	85.78	103.67	107.75
	BT-549	103.01	94.08	100.47	100.62	62.13	95.93	98.09
	T-47D	100.8	95.24	65.98	91.53	42.5	75.49	103.34
	MDA-MB-468	85.34	96.59	26.64	94.03	33.2	66.94	94.39
	**Mean**	106.53	94.15	82.85	88.31	72.07	91.78	103.35
	**Delta**	23.16	183.97	169.18	178.45	38.87	30.23	19.99
	**Range**	72.47	244.17	208.67	209.18	75.47	75.25	49.14

2.1 6a

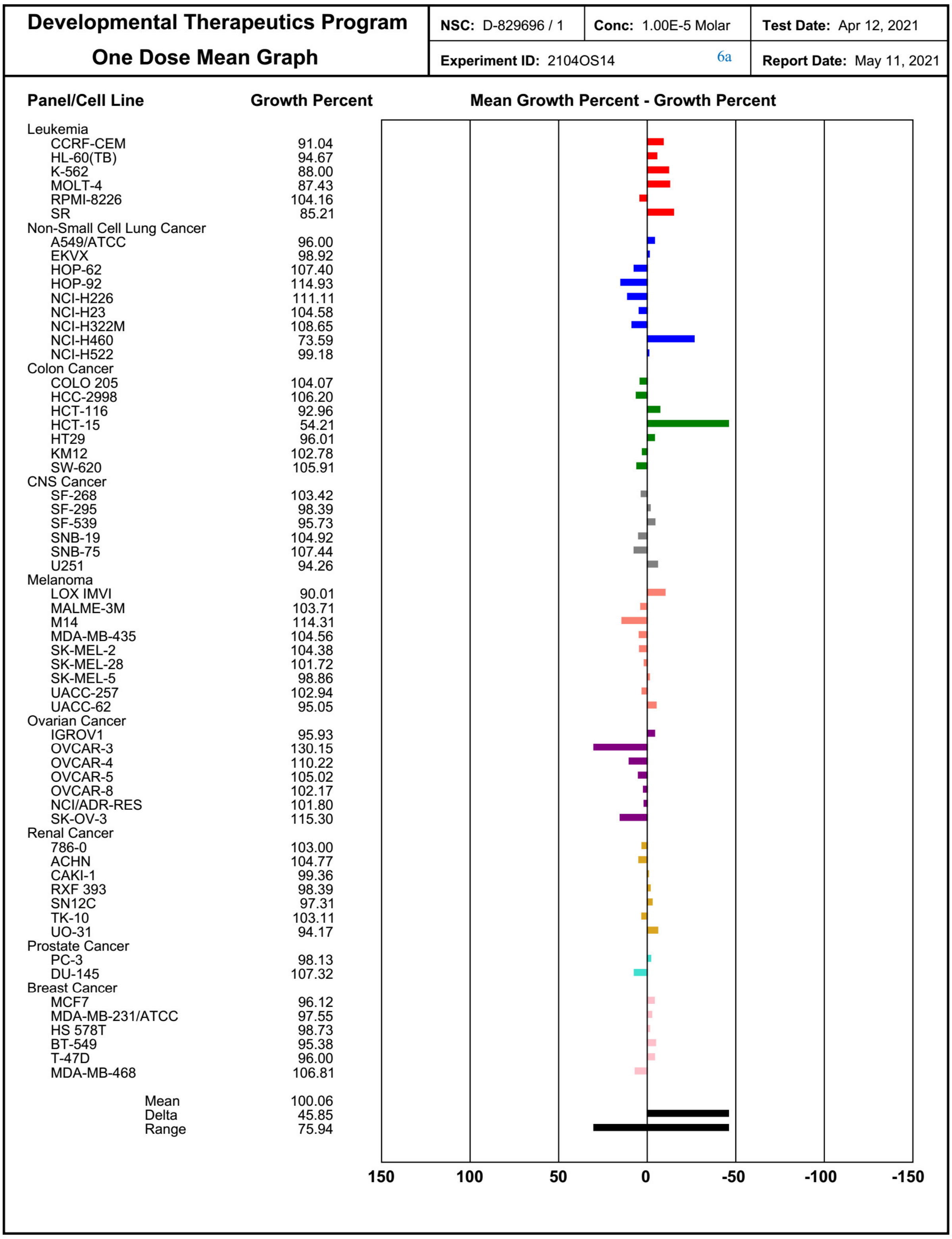

Figure S56. One dose mean graph of **6a**.

2.2 6b

Developmental Therapeutics Program

One Dose Mean Graph

**NSC:** D-828311 / 1	**Conc:** 1.00E-5 Molar	**Test Date:** Mar 01, 2021	
**Experiment ID:** 2103OS90	6b	**Report Date:** Mar 22, 2021	

Panel/Cell Line	Growth Percent
**Leukemia**	
CCRF-CEM	102.25
HL-60(TB)	122.25
K-562	111.46
MOLT-4	117.37
RPMI-8226	114.03
SR	89.14
**Non-Small Cell Lung Cancer**	
A549/ATCC	100.55
EKVX	93.09
HOP-62	102.98
HOP-92	91.42
NCI-H226	94.74
NCI-H23	101.56
NCI-H322M	99.46
NCI-H460	110.90
NCI-H522	96.28
**Colon Cancer**	
COLO 205	114.17
HCC-2998	108.15
HCT-116	99.24
HCT-15	96.40
HT29	105.66
KM12	101.83
SW-620	120.29
**CNS Cancer**	
SF-268	102.96
SF-295	97.09
SF-539	101.85
SNB-19	99.29
U251	103.53
**Melanoma**	
LOX IMVI	97.58
MALME-3M	99.35
M14	102.97
MDA-MB-435	99.70
SK-MEL-2	109.32
SK-MEL-28	100.93
SK-MEL-5	102.79
UACC-257	101.17
UACC-62	88.41
**Ovarian Cancer**	
IGROV1	101.42
OVCAR-3	108.51
OVCAR-4	101.92
OVCAR-5	105.87
OVCAR-8	105.16
NCI/ADR-RES	103.18
SK-OV-3	109.92
**Renal Cancer**	
786-0	95.78
A498	101.84
ACHN	104.80
CAKI-1	87.57
RXF 393	110.52
SN12C	100.82
TK-10	94.81
UO-31	95.03
**Prostate Cancer**	
PC-3	103.14
DU-145	104.51
**Breast Cancer**	
MCF7	92.38
MDA-MB-231/ATCC	106.00
HS 578T	109.67
BT-549	94.95
T-47D	105.60
MDA-MB-468	94.26
Mean	102.34
Delta	14.77
Range	34.68

Mean Growth Percent - Growth Percent

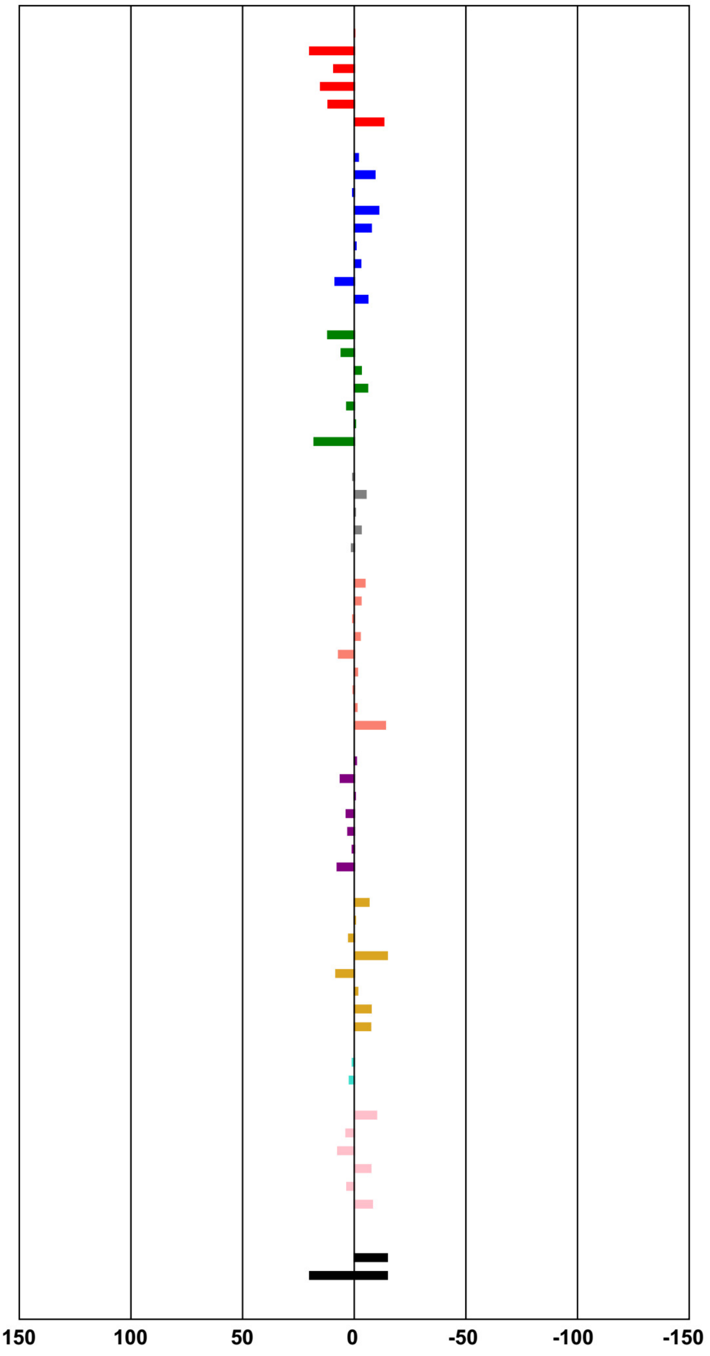

Figure S57. One dose mean graph of **6b**.

2.3 6c

Developmental Therapeutics Program	NSC: D-828310 / 1	Conc: 1.00E-5 Molar	Test Date: Mar 01, 2021
One Dose Mean Graph	Experiment ID: 2103OS90	6c	Report Date: Mar 22, 2021

Panel/Cell Line	Growth Percent
**Leukemia**	
CCRF-CEM	97.37
HL-60(TB)	118.81
K-562	90.21
MOLT-4	92.87
RPMI-8226	93.99
SR	73.92
**Non-Small Cell Lung Cancer**	
A549/ATCC	99.30
EKVX	90.37
HOP-62	105.46
HOP-92	91.18
NCI-H226	97.32
NCI-H23	98.12
NCI-H322M	90.96
NCI-H460	109.35
NCI-H522	84.78
**Colon Cancer**	
COLO 205	116.19
HCC-2998	105.91
HCT-116	81.85
HCT-15	91.51
HT29	93.24
KM12	99.82
SW-620	106.71
**CNS Cancer**	
SF-268	96.49
SF-295	99.05
SF-539	96.18
SNB-19	100.81
U251	97.24
**Melanoma**	
LOX IMVI	100.63
MALME-3M	98.19
M14	90.27
MDA-MB-435	93.40
SK-MEL-2	102.54
SK-MEL-28	101.78
SK-MEL-5	90.37
UACC-257	104.84
UACC-62	81.38
**Ovarian Cancer**	
IGROV1	97.12
OVCAR-3	105.27
OVCAR-4	90.57
OVCAR-5	103.77
OVCAR-8	101.28
NCI/ADR-RES	91.74
SK-OV-3	104.32
**Renal Cancer**	
786-0	106.76
A498	86.35
ACHN	97.31
CAKI-1	85.97
RXF 393	112.21
SN12C	87.90
TK-10	110.09
UO-31	77.03
**Prostate Cancer**	
PC-3	83.52
DU-145	105.81
**Breast Cancer**	
MCF7	83.01
MDA-MB-231/ATCC	95.65
HS 578T	97.53
BT-549	101.30
T-47D	71.97
MDA-MB-468	97.08
Mean	96.20
Delta	24.23
Range	46.84

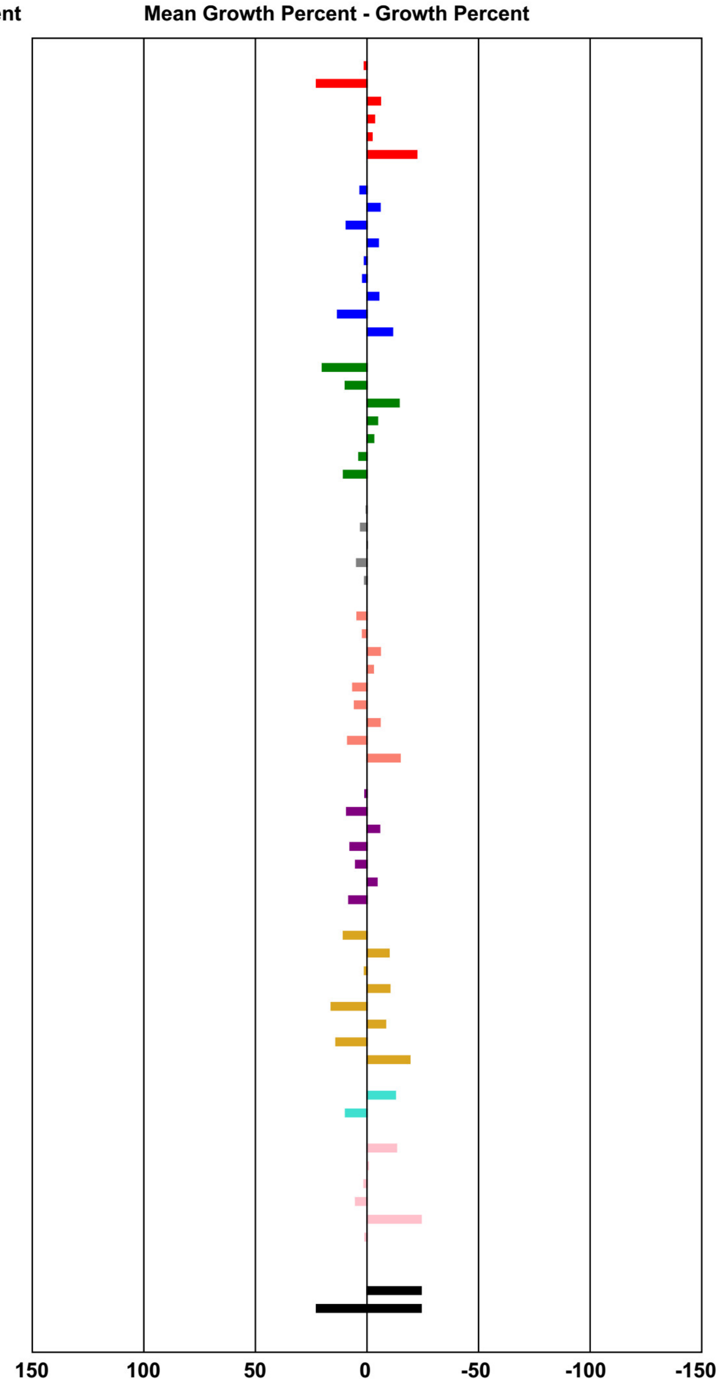

Figure S58. One dose mean graph of **6c**.

2.4 6d

Developmental Therapeutics Program

One Dose Mean Graph

**NSC:** D-828309 / 1	**Conc:** 1.00E-5 Molar	**Test Date:** Mar 01, 2021
**Experiment ID:** 2103OS90 6d		**Report Date:** Mar 22, 2021

Panel/Cell Line	Growth Percent
Leukemia	
CCRF-CEM	112.57
HL-60(TB)	111.45
K-562	99.35
MOLT-4	110.12
RPMI-8226	106.01
SR	102.41
Non-Small Cell Lung Cancer	
A549/ATCC	103.09
EKVX	101.70
HOP-62	106.91
HOP-92	103.50
NCI-H226	101.32
NCI-H23	102.10
NCI-H322M	99.86
NCI-H460	111.70
NCI-H522	104.58
Colon Cancer	
COLO 205	106.94
HCC-2998	107.21
HCT-116	103.76
HCT-15	103.97
HT29	119.10
KM12	103.66
SW-620	118.65
CNS Cancer	
SF-268	105.79
SF-295	107.96
SF-539	99.82
SNB-19	99.60
U251	105.73
Melanoma	
LOX IMVI	96.64
MALME-3M	101.49
M14	96.17
MDA-MB-435	104.96
SK-MEL-2	120.98
SK-MEL-28	101.92
SK-MEL-5	97.99
UACC-257	109.22
UACC-62	89.61
Ovarian Cancer	
IGROV1	106.25
OVCAR-3	113.83
OVCAR-4	102.62
OVCAR-5	100.77
OVCAR-8	107.45
NCI/ADR-RES	102.30
SK-OV-3	109.35
Renal Cancer	
786-0	107.35
A498	111.89
ACHN	95.53
CAKI-1	101.17
RXF 393	118.64
SN12C	98.39
TK-10	125.68
UO-31	88.52
Prostate Cancer	
PC-3	99.01
DU-145	111.53
Breast Cancer	
MCF7	95.41
MDA-MB-231/ATCC	101.12
HS 578T	101.62
BT-549	102.15
T-47D	104.36
MDA-MB-468	107.05
Mean	104.91
Delta	16.39
Range	37.16

Mean Growth Percent - Growth Percent

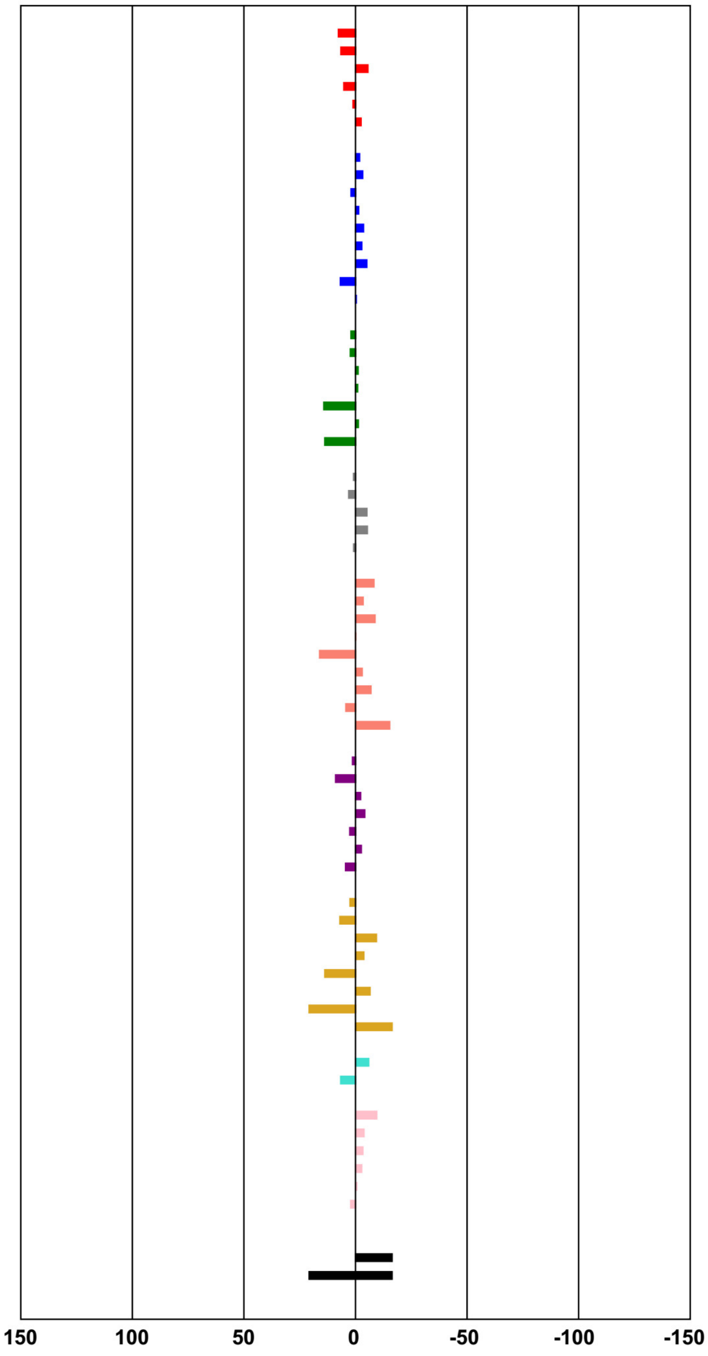

Figure S59. One dose mean graph of **6d**.

2.5 6e

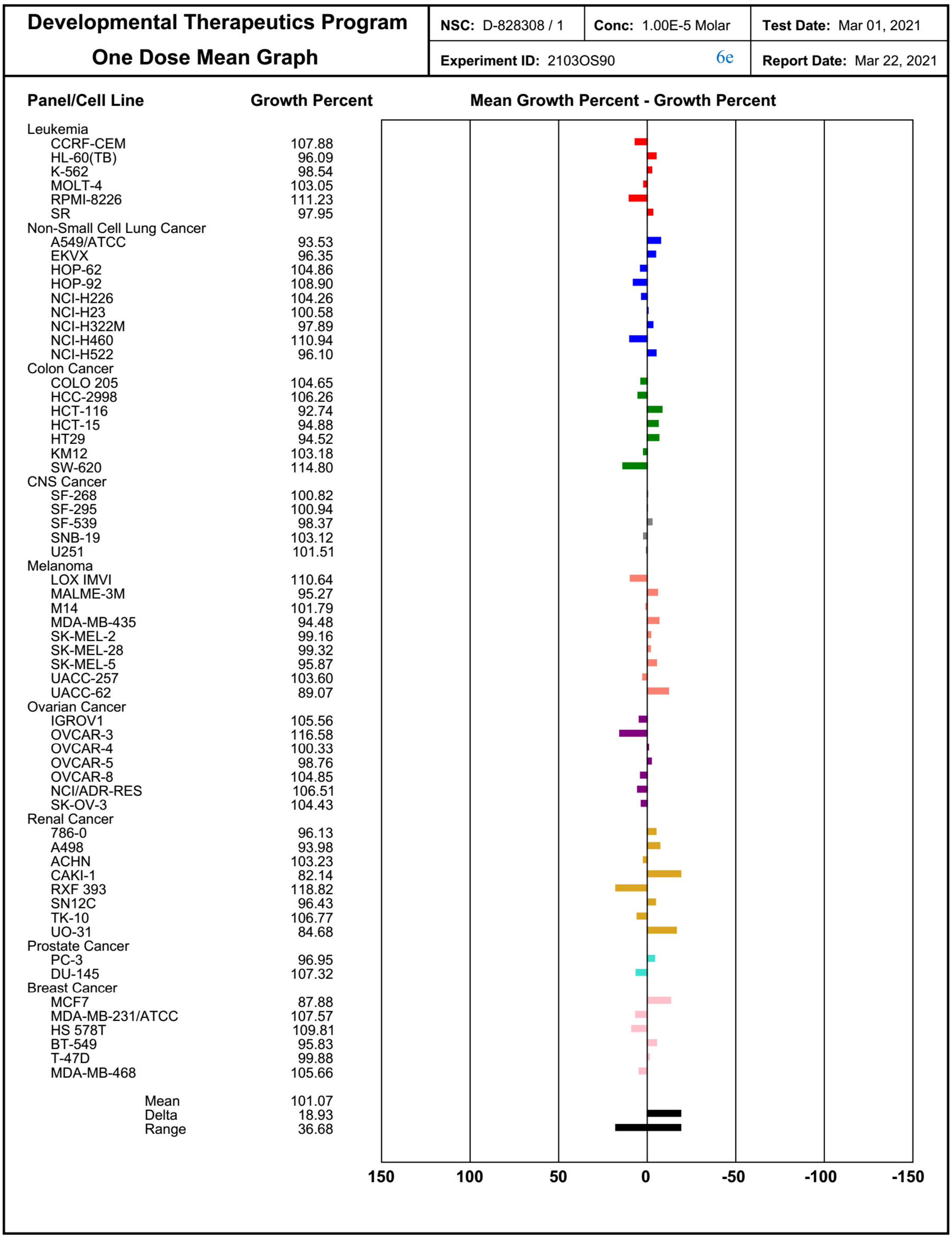

Developmental Therapeutics Program | **NSC:** D-828308 / 1 | **Conc:** 1.00E-5 Molar | **Test Date:** Mar 01, 2021

One Dose Mean Graph | **Experiment ID:** 2103OS90 | 6e | **Report Date:** Mar 22, 2021

Panel/Cell Line	Growth Percent
**Leukemia**	
CCRF-CEM	107.88
HL-60(TB)	96.09
K-562	98.54
MOLT-4	103.05
RPMI-8226	111.23
SR	97.95
**Non-Small Cell Lung Cancer**	
A549/ATCC	93.53
EKVX	96.35
HOP-62	104.86
HOP-92	108.90
NCI-H226	104.26
NCI-H23	100.58
NCI-H322M	97.89
NCI-H460	110.94
NCI-H522	96.10
**Colon Cancer**	
COLO 205	104.65
HCC-2998	106.26
HCT-116	92.74
HCT-15	94.88
HT29	94.52
KM12	103.18
SW-620	114.80
**CNS Cancer**	
SF-268	100.82
SF-295	100.94
SF-539	98.37
SNB-19	103.12
U251	101.51
**Melanoma**	
LOX IMVI	110.64
MALME-3M	95.27
M14	101.79
MDA-MB-435	94.48
SK-MEL-2	99.16
SK-MEL-28	99.32
SK-MEL-5	95.87
UACC-257	103.60
UACC-62	89.07
**Ovarian Cancer**	
IGROV1	105.56
OVCAR-3	116.58
OVCAR-4	100.33
OVCAR-5	98.76
OVCAR-8	104.85
NCI/ADR-RES	106.51
SK-OV-3	104.43
**Renal Cancer**	
786-0	96.13
A498	93.98
ACHN	103.23
CAKI-1	82.14
RXF 393	118.82
SN12C	96.43
TK-10	106.77
UO-31	84.68
**Prostate Cancer**	
PC-3	96.95
DU-145	107.32
**Breast Cancer**	
MCF7	87.88
MDA-MB-231/ATCC	107.57
HS 578T	109.81
BT-549	95.83
T-47D	99.88
MDA-MB-468	105.66
Mean	101.07
Delta	18.93
Range	36.68

Figure S60. One dose mean graph of **6e**.

2.6 6f

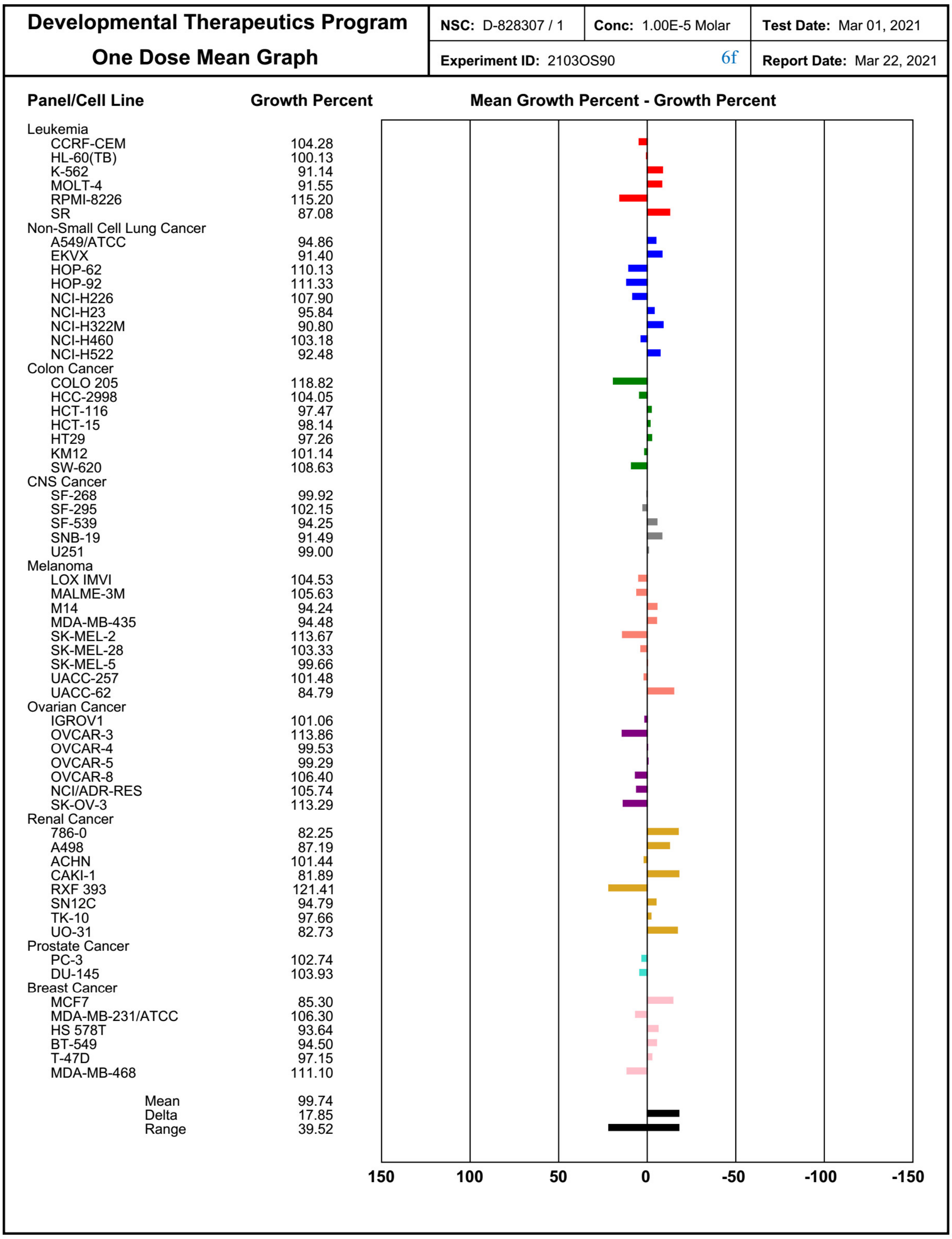

Figure S61. One dose mean graph of **6f**.

2.7 6g

Developmental Therapeutics Program One Dose Mean Graph	NSC: D-828306 / 1	Conc: 1.00E-5 Molar	Test Date: Mar 01, 2021
	Experiment ID: 2103OS90	6g	Report Date: Mar 22, 2021

Panel/Cell Line	Growth Percent
Leukemia	
CCRF-CEM	98.11
HL-60(TB)	115.37
K-562	103.09
MOLT-4	97.69
RPMI-8226	108.22
SR	120.70
Non-Small Cell Lung Cancer	
A549/ATCC	97.27
EKVX	83.47
HOP-62	94.01
HOP-92	86.53
NCI-H226	89.92
NCI-H23	94.95
NCI-H322M	94.49
NCI-H460	100.15
NCI-H522	92.02
Colon Cancer	
COLO 205	116.74
HCC-2998	104.45
HCT-116	90.71
HCT-15	97.15
HT29	101.19
KM12	98.60
SW-620	106.99
CNS Cancer	
SF-268	104.45
SF-295	87.58
SF-539	90.41
SNB-19	99.19
U251	102.06
Melanoma	
LOX IMVI	94.96
MALME-3M	91.61
M14	94.00
MDA-MB-435	101.38
SK-MEL-2	101.23
SK-MEL-28	99.45
SK-MEL-5	96.52
UACC-257	97.09
UACC-62	80.30
Ovarian Cancer	
IGROV1	88.15
OVCAR-3	111.90
OVCAR-4	95.72
OVCAR-5	99.26
OVCAR-8	93.92
NCI/ADR-RES	93.83
SK-OV-3	96.19
Renal Cancer	
786-0	121.94
A498	106.36
ACHN	88.01
CAKI-1	84.89
RXF 393	131.01
SN12C	91.26
TK-10	122.57
UO-31	81.52
Prostate Cancer	
PC-3	90.94
DU-145	109.58
Breast Cancer	
MCF7	89.04
MDA-MB-231/ATCC	84.90
HS 578T	93.41
BT-549	104.23
T-47D	98.47
MDA-MB-468	97.42
Mean	98.42
Delta	18.12
Range	50.71

Mean Growth Percent - Growth Percent

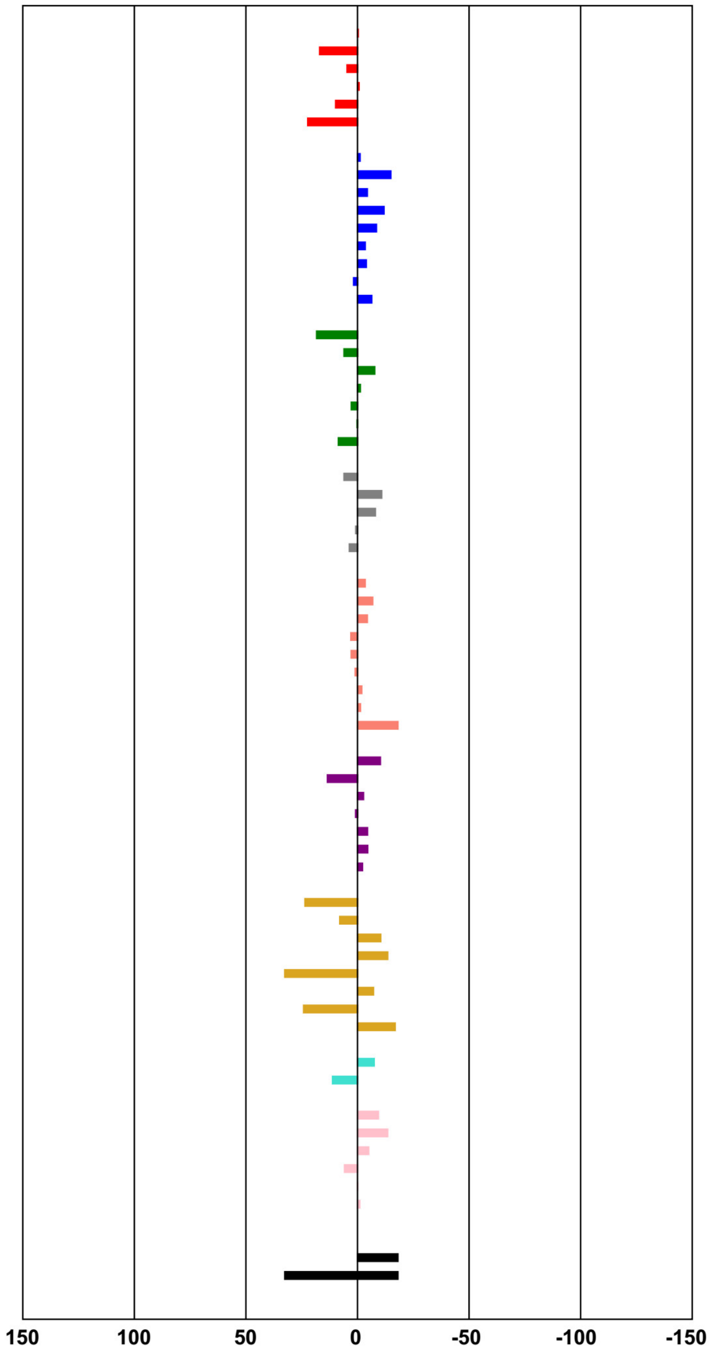

Figure S62. One dose mean graph of **6g**.

2.8 6h

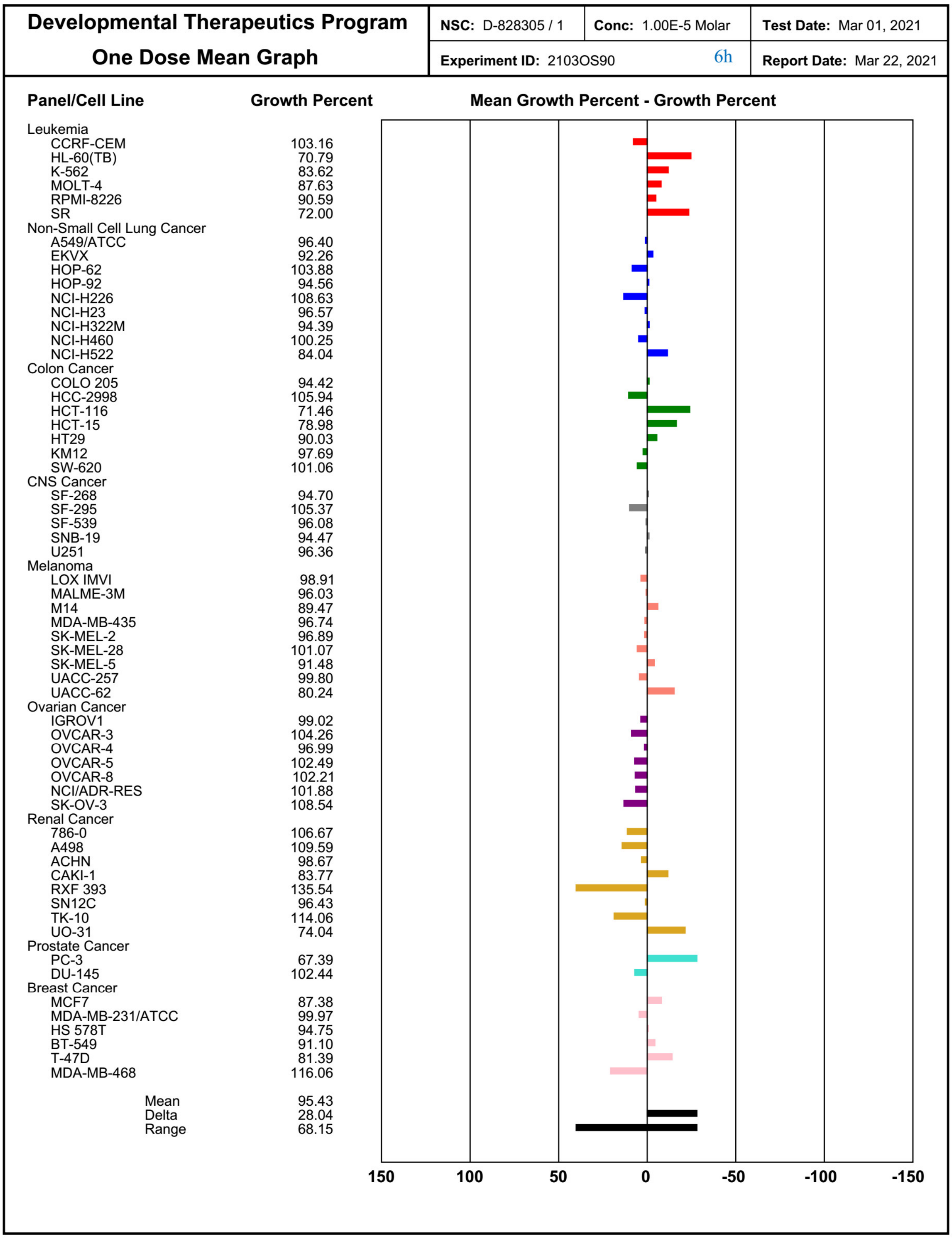

Figure S63. One dose mean graph of **6h.**

2.9 11

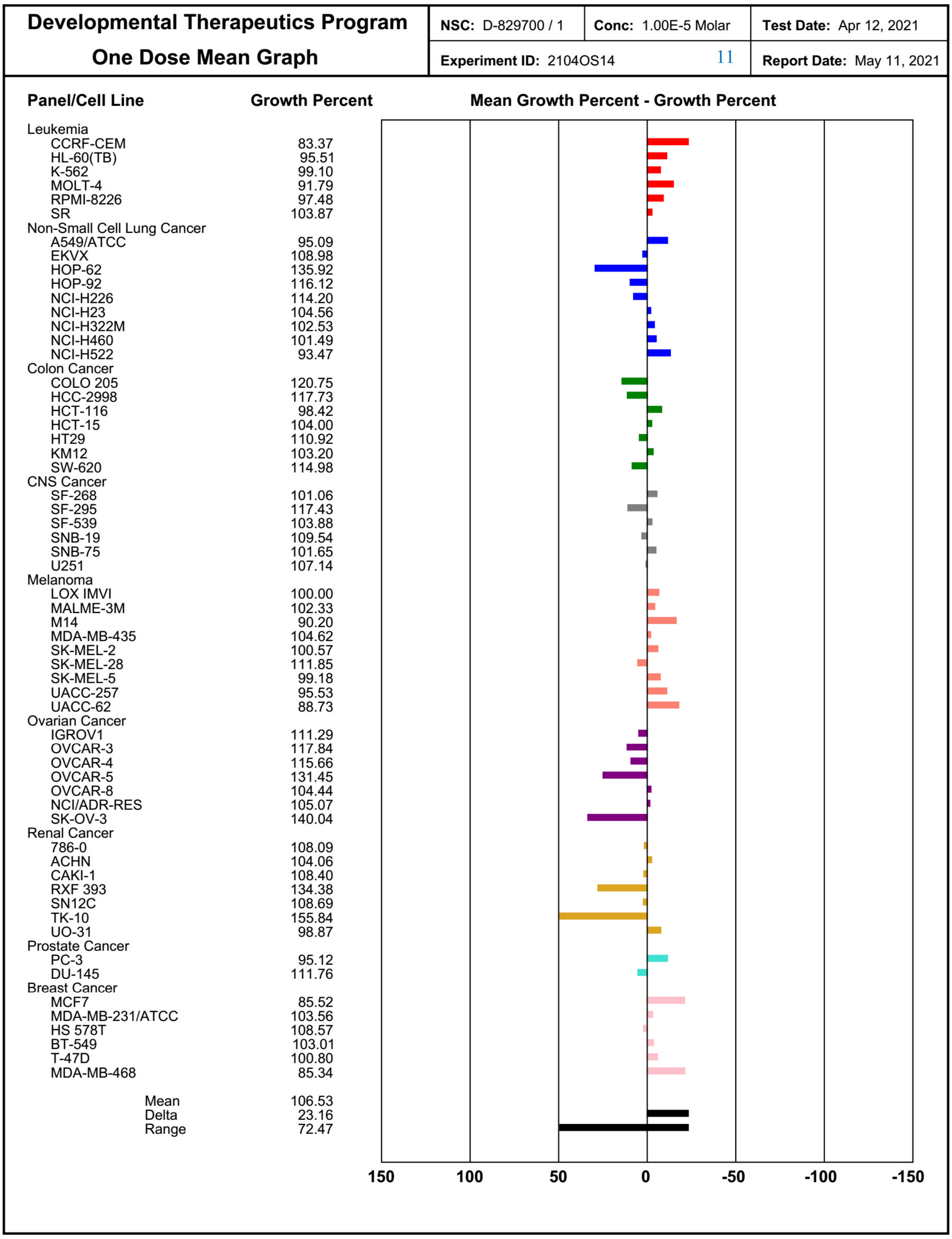

Figure S64. One dose mean graph of **11**.

2.10 12a

Developmental Therapeutics Program	NSC: D-828304 / 1	Conc: 1.00E-5 Molar	Test Date: Mar 01, 2021
One Dose Mean Graph	Experiment ID: 2103OS90	12a	Report Date: Mar 22, 2021

Panel/Cell Line	Growth Percent
Leukemia	
CCRF-CEM	59.23
HL-60(TB)	77.18
K-562	39.80
MOLT-4	56.39
RPMI-8226	83.41
SR	58.67
Non-Small Cell Lung Cancer	
A549/ATCC	76.64
EKVX	73.16
HOP-62	93.10
HOP-92	90.13
NCI-H226	85.58
NCI-H23	96.74
NCI-H322M	77.61
NCI-H460	52.95
NCI-H522	91.36
Colon Cancer	
COLO 205	73.59
HCC-2998	85.58
HCT-116	41.11
HCT-15	41.95
HT29	61.53
KM12	56.73
SW-620	85.99
CNS Cancer	
SF-268	68.61
SF-295	76.59
SF-539	78.06
SNB-19	88.00
U251	69.93
Melanoma	
LOX IMVI	57.95
MALME-3M	91.04
M14	68.70
MDA-MB-435	78.65
SK-MEL-2	105.94
SK-MEL-28	92.40
SK-MEL-5	70.32
UACC-257	102.82
UACC-62	64.87
Ovarian Cancer	
IGROV1	77.88
OVCAR-3	105.05
OVCAR-4	70.76
OVCAR-5	93.72
OVCAR-8	89.70
NCI/ADR-RES	83.00
SK-OV-3	96.87
Renal Cancer	
786-0	92.39
A498	94.43
ACHN	77.73
CAKI-1	53.03
RXF 393	86.80
SN12C	77.71
TK-10	94.39
UO-31	57.52
Prostate Cancer	
PC-3	45.65
DU-145	82.25
Breast Cancer	
MCF7	58.52
MDA-MB-231/ATCC	84.40
HS 578T	77.88
BT-549	103.19
T-47D	80.45
MDA-MB-468	75.83
Mean	76.80
Delta	37.00
Range	66.14

Mean Growth Percent - Growth Percent

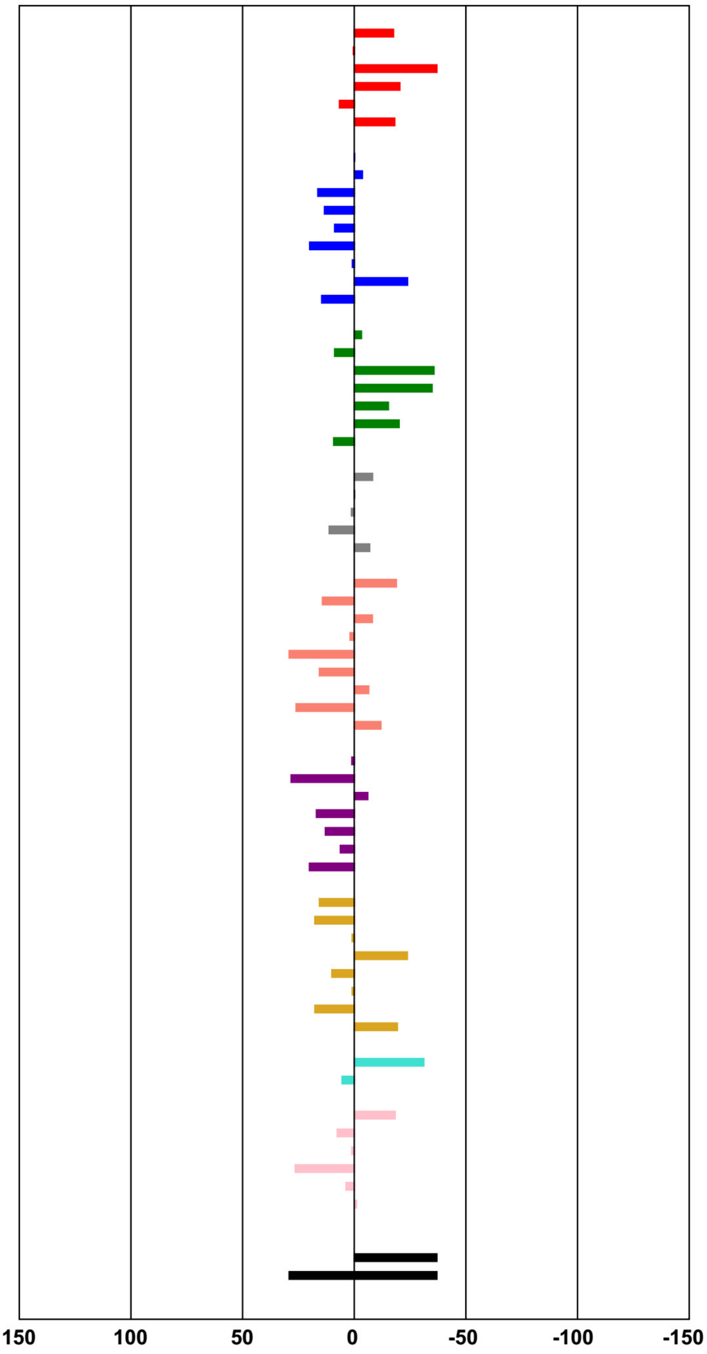

Figure S65. One dose mean graph of **12a.**

2.11 12b

Developmental Therapeutics Program | **NSC:** D-828303 / 1 | **Conc:** 1.00E-5 Molar | **Test Date:** Mar 01, 2021

One Dose Mean Graph | **Experiment ID:** 2103OS90 12b | **Report Date:** Mar 22, 2021

Panel/Cell Line	Growth Percent
Leukemia	
CCRF-CEM	48.49
HL-60(TB)	79.82
K-562	22.77
MOLT-4	46.97
RPMI-8226	73.80
SR	43.47
Non-Small Cell Lung Cancer	
A549/ATCC	64.44
EKVX	65.40
HOP-62	77.94
HOP-92	68.36
NCI-H226	76.99
NCI-H23	72.37
NCI-H322M	74.07
NCI-H460	35.58
NCI-H522	72.57
Colon Cancer	
COLO 205	74.58
HCC-2998	75.10
HCT-116	46.67
HCT-15	38.07
HT29	43.90
KM12	41.24
SW-620	84.76
CNS Cancer	
SF-268	64.95
SF-295	54.52
SF-539	68.29
SNB-19	78.94
U251	73.17
Melanoma	
LOX IMVI	27.94
MALME-3M	93.53
M14	68.18
MDA-MB-435	72.77
SK-MEL-2	99.80
SK-MEL-28	89.22
SK-MEL-5	86.00
UACC-257	102.46
UACC-62	64.92
Ovarian Cancer	
IGROV1	60.07
OVCAR-3	91.97
OVCAR-4	78.52
OVCAR-5	77.15
OVCAR-8	72.77
NCI/ADR-RES	66.38
SK-OV-3	88.84
Renal Cancer	
786-0	67.15
A498	97.40
ACHN	56.91
CAKI-1	31.10
RXF 393	51.18
SN12C	57.98
TK-10	86.83
UO-31	43.42
Prostate Cancer	
PC-3	48.22
DU-145	70.76
Breast Cancer	
MCF7	38.79
MDA-MB-231/ATCC	69.70
HS 578T	77.61
BT-549	97.50
T-47D	68.83
MDA-MB-468	71.95
Mean	66.83
Delta	44.06
Range	79.69

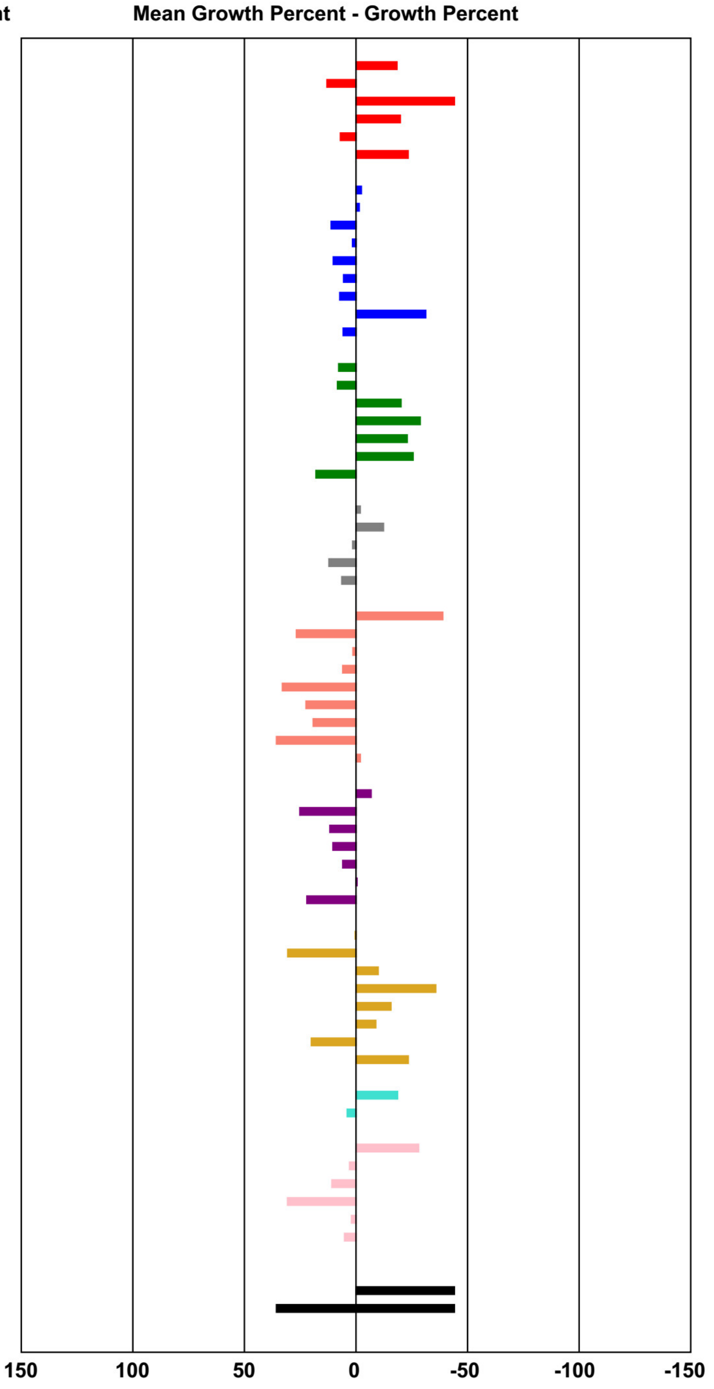

Figure S66. One dose mean graph of **12b.**

2.12 12c

Developmental Therapeutics Program | **NSC:** D-828302 / 1 | **Conc:** 1.00E-5 Molar | **Test Date:** Mar 01, 2021

One Dose Mean Graph | **Experiment ID:** 2103OS90 | 12c | **Report Date:** Mar 22, 2021

Panel/Cell Line	Growth Percent
Leukemia	
CCRF-CEM	60.40
HL-60(TB)	83.19
K-562	51.35
MOLT-4	60.31
RPMI-8226	91.13
SR	55.48
Non-Small Cell Lung Cancer	
A549/ATCC	82.38
EKVX	71.11
HOP-62	68.33
HOP-92	82.26
NCI-H226	74.96
NCI-H23	91.64
NCI-H322M	85.06
NCI-H460	46.57
NCI-H522	83.51
Colon Cancer	
COLO 205	73.25
HCC-2998	96.07
HCT-116	31.88
HCT-15	34.60
HT29	70.41
KM12	74.26
SW-620	79.72
CNS Cancer	
SF-268	73.20
SF-295	57.45
SF-539	59.37
SNB-19	84.13
U251	54.13
Melanoma	
LOX IMVI	48.54
MALME-3M	77.70
M14	71.90
MDA-MB-435	86.57
SK-MEL-2	103.84
SK-MEL-28	89.51
SK-MEL-5	61.69
UACC-257	95.70
UACC-62	62.51
Ovarian Cancer	
IGROV1	59.62
OVCAR-3	91.56
OVCAR-4	81.28
OVCAR-5	89.54
OVCAR-8	75.76
NCI/ADR-RES	88.75
SK-OV-3	82.71
Renal Cancer	
786-0	88.11
A498	116.68
ACHN	68.07
CAKI-1	55.05
RXF 393	89.20
SN12C	76.16
TK-10	109.09
UO-31	50.26
Prostate Cancer	
PC-3	46.96
DU-145	84.78
Breast Cancer	
MCF7	68.23
MDA-MB-231/ATCC	65.90
HS 578T	83.15
BT-549	97.42
T-47D	85.62
MDA-MB-468	84.20
Mean	74.78
Delta	42.90
Range	84.80

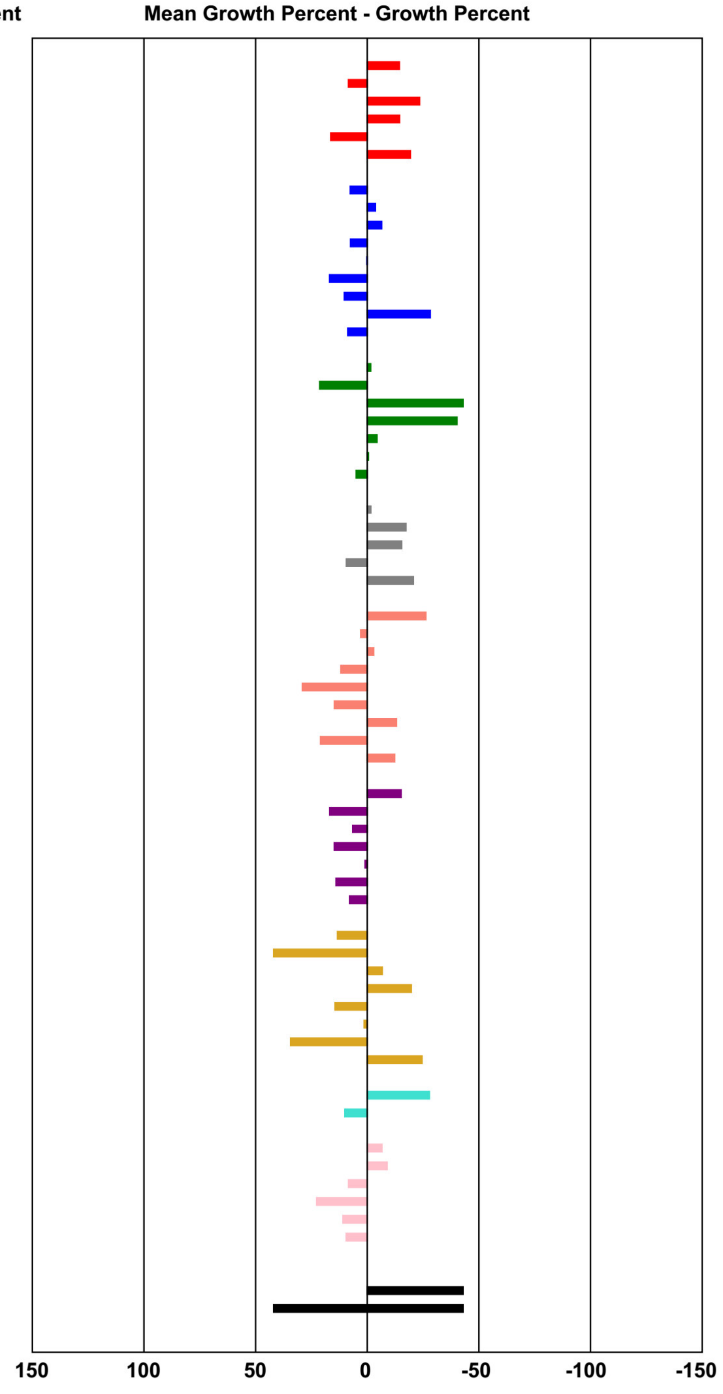

Figure S67. One dose mean graph of **12c.**

2.13 13a

Developmental Therapeutics Program

One Dose Mean Graph

**NSC:** D-829701 / 1	**Conc:** 1.00E-5 Molar	**Test Date:** Apr 12, 2021	
**Experiment ID:** 2104OS14	13a	**Report Date:** May 11, 2021	

Panel/Cell Line	Growth Percent
Leukemia	
CCRF-CEM	101.21
HL-60(TB)	108.73
K-562	87.53
MOLT-4	103.59
RPMI-8226	82.53
SR	87.13
Non-Small Cell Lung Cancer	
A549/ATCC	98.55
EKVX	99.95
HOP-62	125.45
HOP-92	100.94
NCI-H226	110.08
NCI-H23	101.47
NCI-H322M	101.08
NCI-H460	101.49
NCI-H522	102.70
Colon Cancer	
COLO 205	113.67
HCC-2998	105.37
HCT-116	94.89
HCT-15	99.88
HT29	114.56
KM12	100.52
SW-620	101.71
CNS Cancer	
SF-268	99.94
SF-295	109.84
SF-539	97.09
SNB-19	96.35
SNB-75	93.65
U251	102.65
Melanoma	
LOX IMVI	-38.51
MALME-3M	110.15
M14	95.67
MDA-MB-435	103.78
SK-MEL-2	-89.82
SK-MEL-28	106.69
SK-MEL-5	98.96
UACC-257	96.17
UACC-62	96.21
Ovarian Cancer	
IGROV1	95.90
OVCAR-3	100.13
OVCAR-4	110.66
OVCAR-5	114.21
OVCAR-8	103.77
NCI/ADR-RES	106.32
SK-OV-3	121.81
Renal Cancer	
786-0	77.86
ACHN	96.68
CAKI-1	97.61
RXF 393	94.11
SN12C	99.78
TK-10	154.35
UO-31	-20.87
Prostate Cancer	
PC-3	104.61
DU-145	98.62
Breast Cancer	
MCF7	79.53
MDA-MB-231/ATCC	101.02
HS 578T	110.78
BT-549	94.08
T-47D	95.24
MDA-MB-468	96.59
Mean	94.15
Delta	183.97
Range	244.17

Mean Growth Percent - Growth Percent

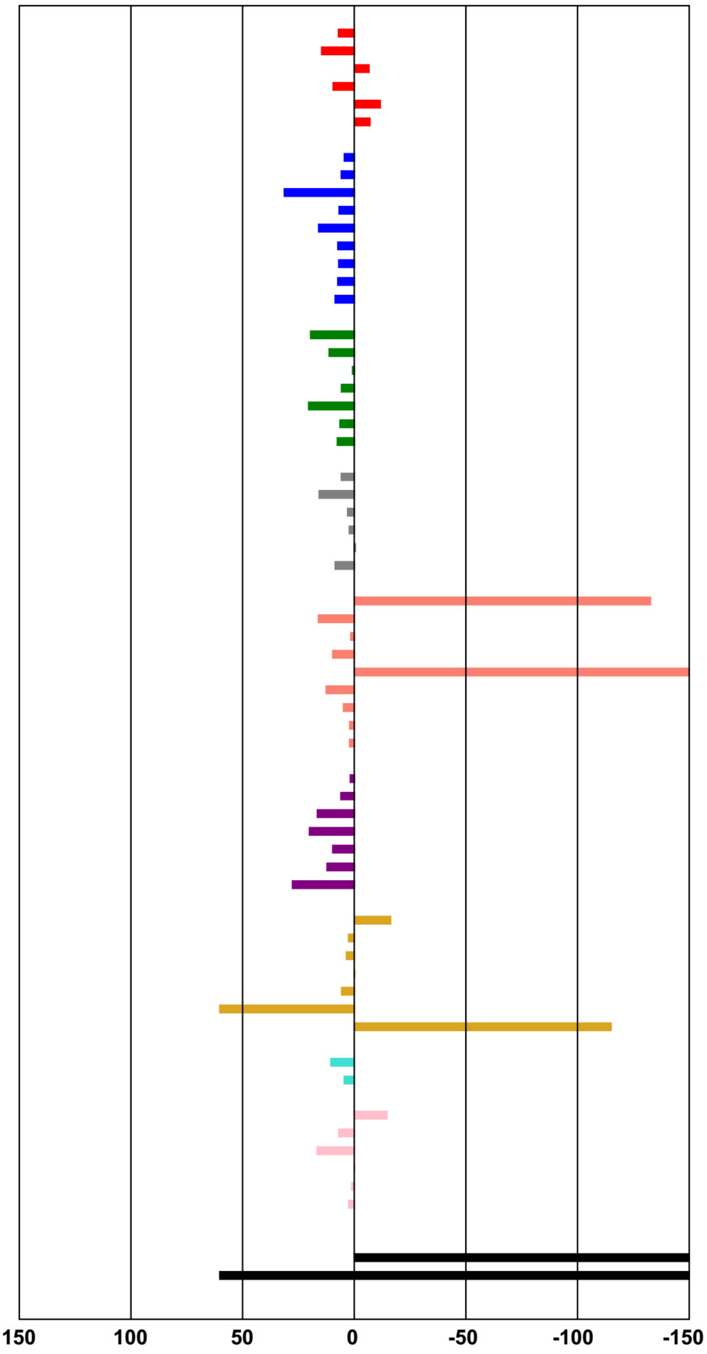

Figure S68. One dose mean graph of **13a**.

2.14 13b

Developmental Therapeutics Program	NSC: D-829702 / 1	Conc: 1.00E-5 Molar	Test Date: Apr 12, 2021
One Dose Mean Graph	Experiment ID: 2104OS14	13b	Report Date: May 11, 2021

Panel/Cell Line	Growth Percent
Leukemia	
HL-60(TB)	33.07
K-562	19.16
MOLT-4	79.63
SR	39.76
Non-Small Cell Lung Cancer	
A549/ATCC	97.67
EKVX	99.24
HOP-62	98.34
HOP-92	117.39
NCI-H226	111.37
NCI-H23	98.81
NCI-H322M	102.74
NCI-H460	102.13
NCI-H522	89.79
Colon Cancer	
COLO 205	110.35
HCC-2998	107.90
HCT-116	24.88
HCT-15	90.57
HT29	105.88
KM12	53.31
SW-620	85.06
CNS Cancer	
SF-268	106.41
SF-295	109.05
SF-539	36.89
SNB-19	89.43
SNB-75	93.92
U251	100.54
Melanoma	
LOX IMVI	2.09
MALME-3M	102.62
M14	98.99
MDA-MB-435	97.90
SK-MEL-2	104.62
SK-MEL-28	109.98
SK-MEL-5	98.75
UACC-257	97.90
UACC-62	96.16
Ovarian Cancer	
IGROV1	97.39
OVCAR-3	90.89
OVCAR-4	109.54
OVCAR-5	112.48
OVCAR-8	102.43
NCI/ADR-RES	104.73
SK-OV-3	122.34
Renal Cancer	
786-0	4.55
ACHN	90.25
CAKI-1	99.62
RXF 393	99.19
SN12C	95.71
TK-10	63.25
UO-31	-86.33
Prostate Cancer	
PC-3	102.15
DU-145	83.61
Breast Cancer	
MCF7	16.43
MDA-MB-231/ATCC	88.41
HS 578T	120.34
BT-549	100.47
T-47D	65.98
MDA-MB-468	26.64
Mean	82.85
Delta	169.18
Range	208.67

Mean Growth Percent - Growth Percent

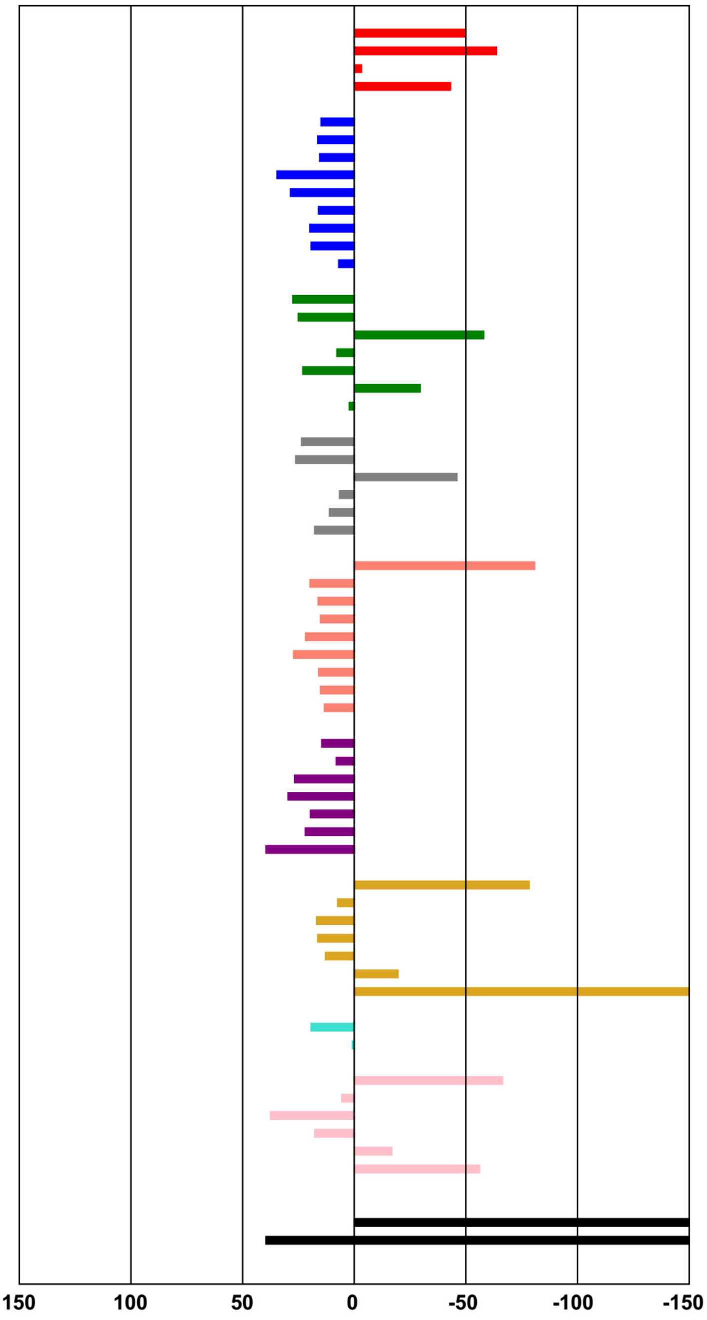

Figure S69. One dose mean graph of **13b**.

2.15 13c

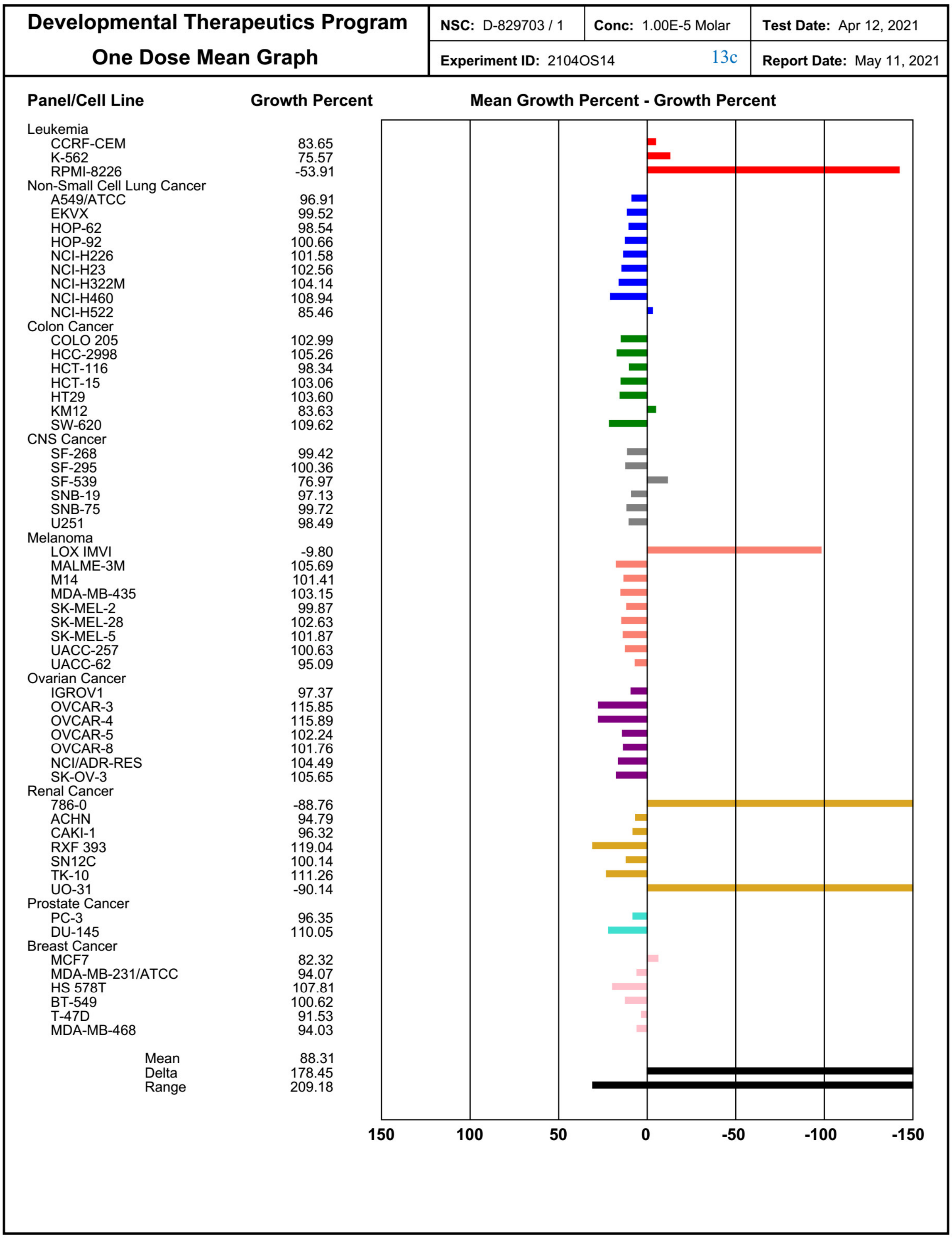

Figure S70. One dose mean graph of **13c**.

2.16 14a

Developmental Therapeutics Program One Dose Mean Graph	NSC: D-829697 / 1	Conc: 1.00E-5 Molar	Test Date: Apr 12, 2021
	Experiment ID: 2104OS14	14a	Report Date: May 11, 2021

Panel/Cell Line	Growth Percent
Leukemia	
CCRF-CEM	57.91
HL-60(TB)	63.09
K-562	49.77
MOLT-4	44.02
RPMI-8226	61.94
SR	45.67
Non-Small Cell Lung Cancer	
A549/ATCC	63.76
EKVX	51.31
HOP-62	104.53
HOP-92	85.39
NCI-H226	77.15
NCI-H23	63.38
NCI-H322M	80.21
NCI-H460	64.19
NCI-H522	60.75
Colon Cancer	
COLO 205	101.27
HCC-2998	84.43
HCT-116	49.64
HCT-15	71.19
HT29	92.01
KM12	73.35
SW-620	92.79
CNS Cancer	
SF-268	73.46
SF-295	71.76
SF-539	92.62
SNB-19	94.01
SNB-75	76.08
U251	85.52
Melanoma	
LOX IMVI	63.61
MALME-3M	80.00
M14	70.69
MDA-MB-435	77.81
SK-MEL-2	77.17
SK-MEL-28	85.29
SK-MEL-5	39.03
UACC-257	60.63
UACC-62	52.51
Ovarian Cancer	
IGROV1	73.46
OVCAR-3	94.45
OVCAR-4	64.29
OVCAR-5	108.67
OVCAR-8	79.36
NCI/ADR-RES	60.57
SK-OV-3	101.89
Renal Cancer	
786-0	85.95
ACHN	70.42
CAKI-1	55.06
RXF 393	98.44
SN12C	78.95
TK-10	102.29
UO-31	55.94
Prostate Cancer	
PC-3	63.34
DU-145	74.63
Breast Cancer	
MCF7	50.05
MDA-MB-231/ATCC	72.55
HS 578T	85.78
BT-549	62.13
T-47D	42.50
MDA-MB-468	33.20
Mean	72.07
Delta	38.87
Range	75.47

Mean Growth Percent - Growth Percent

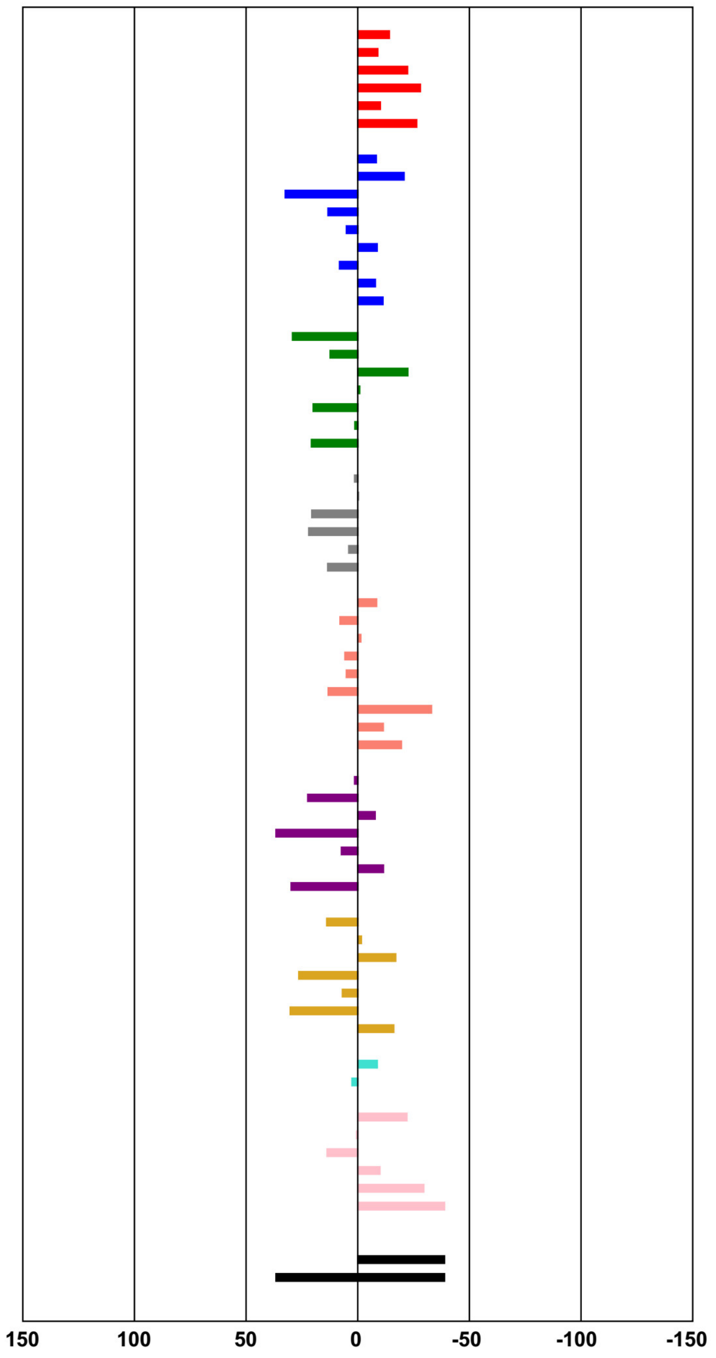

Figure S71. One dose mean graph of **14a**.

2.17 14b

Developmental Therapeutics Program One Dose Mean Graph	NSC: D-829698 / 1	Conc: 1.00E-5 Molar	Test Date: Apr 12, 2021
	Experiment ID: 2104OS14	14b	Report Date: May 11, 2021

Panel/Cell Line	Growth Percent
**Leukemia**	
CCRF-CEM	78.76
HL-60(TB)	93.68
K-562	75.80
MOLT-4	61.55
RPMI-8226	78.99
SR	73.05
**Non-Small Cell Lung Cancer**	
A549/ATCC	74.31
EKVX	83.05
HOP-62	109.59
HOP-92	93.19
NCI-H226	100.85
NCI-H23	86.60
NCI-H322M	88.99
NCI-H460	89.95
NCI-H522	80.86
**Colon Cancer**	
COLO 205	116.22
HCC-2998	110.82
HCT-116	78.98
HCT-15	80.57
HT29	107.47
KM12	94.46
SW-620	109.92
**CNS Cancer**	
SF-268	84.94
SF-295	102.54
SF-539	99.39
SNB-19	100.52
SNB-75	94.56
U251	97.63
**Melanoma**	
LOX IMVI	74.09
MALME-3M	105.91
M14	91.56
MDA-MB-435	100.37
SK-MEL-2	99.23
SK-MEL-28	108.26
SK-MEL-5	90.08
UACC-257	98.47
UACC-62	64.36
**Ovarian Cancer**	
IGROV1	87.63
OVCAR-3	101.91
OVCAR-4	95.44
OVCAR-5	117.17
OVCAR-8	92.40
NCI/ADR-RES	85.44
SK-OV-3	125.21
**Renal Cancer**	
786-0	97.94
ACHN	88.76
CAKI-1	67.06
RXF 393	107.68
SN12C	89.43
TK-10	136.80
UO-31	72.36
**Prostate Cancer**	
PC-3	77.68
DU-145	90.74
**Breast Cancer**	
MCF7	77.00
MDA-MB-231/ATCC	82.67
HS 578T	103.67
BT-549	95.93
T-47D	75.49
MDA-MB-468	66.94
Mean	91.78
Delta	30.23
Range	75.25

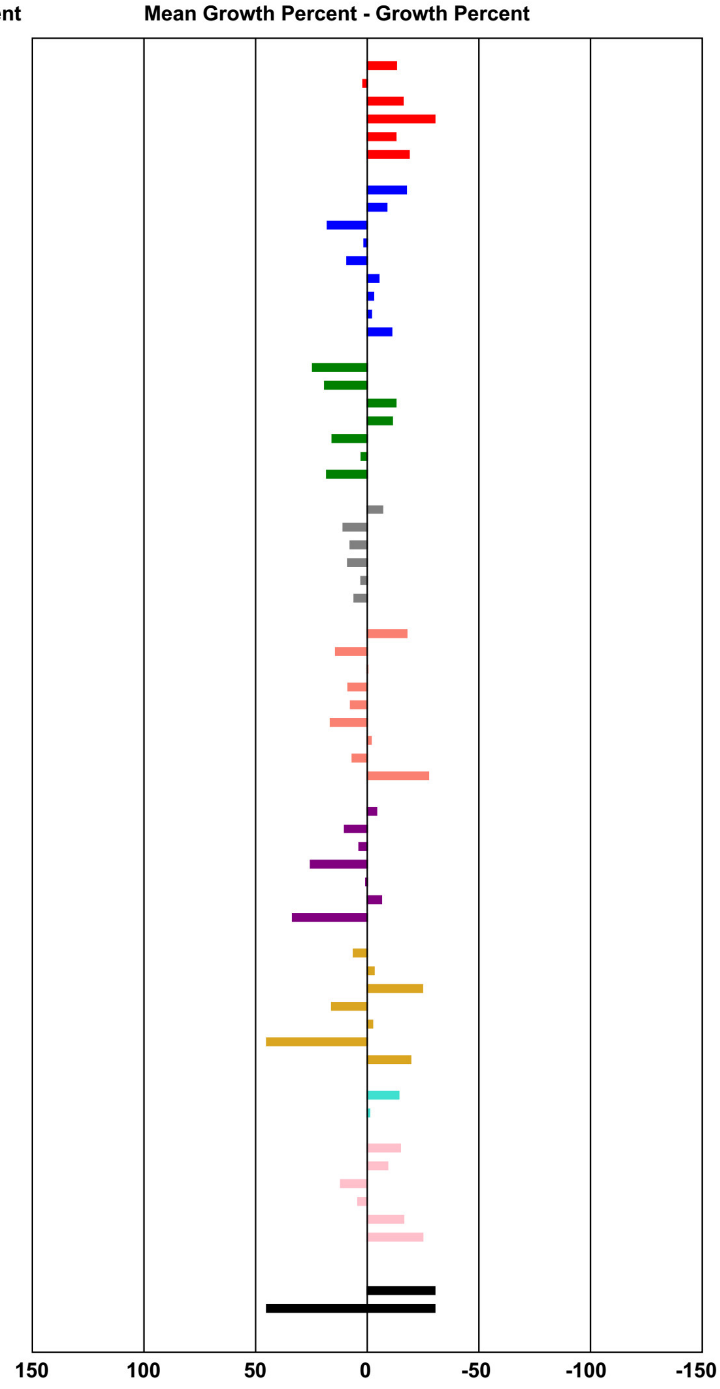

Figure S72. One dose mean graph of **14b**.

2.18 14c

Developmental Therapeutics Program One Dose Mean Graph	NSC: D-829699 / 1	Conc: 1.00E-5 Molar	Test Date: Apr 12, 2021
	Experiment ID: 2104OS14	14c	Report Date: May 11, 2021

Panel/Cell Line	Growth Percent
Leukemia	
CCRF-CEM	105.22
HL-60(TB)	103.20
K-562	110.66
MOLT-4	87.17
RPMI-8226	111.75
SR	95.15
Non-Small Cell Lung Cancer	
A549/ATCC	97.39
EKVX	98.93
HOP-62	116.09
HOP-92	107.67
NCI-H226	108.98
NCI-H23	103.09
NCI-H322M	104.06
NCI-H460	106.97
NCI-H522	90.77
Colon Cancer	
COLO 205	114.51
HCC-2998	112.32
HCT-116	93.76
HCT-15	97.31
HT29	110.02
KM12	105.52
SW-620	111.22
CNS Cancer	
SF-268	95.24
SF-295	115.30
SF-539	101.75
SNB-19	101.91
SNB-75	99.13
U251	101.98
Melanoma	
LOX IMVI	92.51
MALME-3M	106.53
M14	101.36
MDA-MB-435	106.96
SK-MEL-2	102.96
SK-MEL-28	103.56
SK-MEL-5	101.25
UACC-257	105.22
UACC-62	85.58
Ovarian Cancer	
IGROV1	93.90
OVCAR-3	127.06
OVCAR-4	110.28
OVCAR-5	111.71
OVCAR-8	101.27
NCI/ADR-RES	106.58
SK-OV-3	119.28
Renal Cancer	
786-0	104.38
ACHN	96.91
CAKI-1	90.20
RXF 393	116.98
SN12C	98.75
TK-10	132.50
UO-31	83.36
Prostate Cancer	
PC-3	96.61
DU-145	106.83
Breast Cancer	
MCF7	88.10
MDA-MB-231/ATCC	96.45
HS 578T	107.75
BT-549	98.09
T-47D	103.34
MDA-MB-468	94.39
Mean	103.35
Delta	19.99
Range	49.14

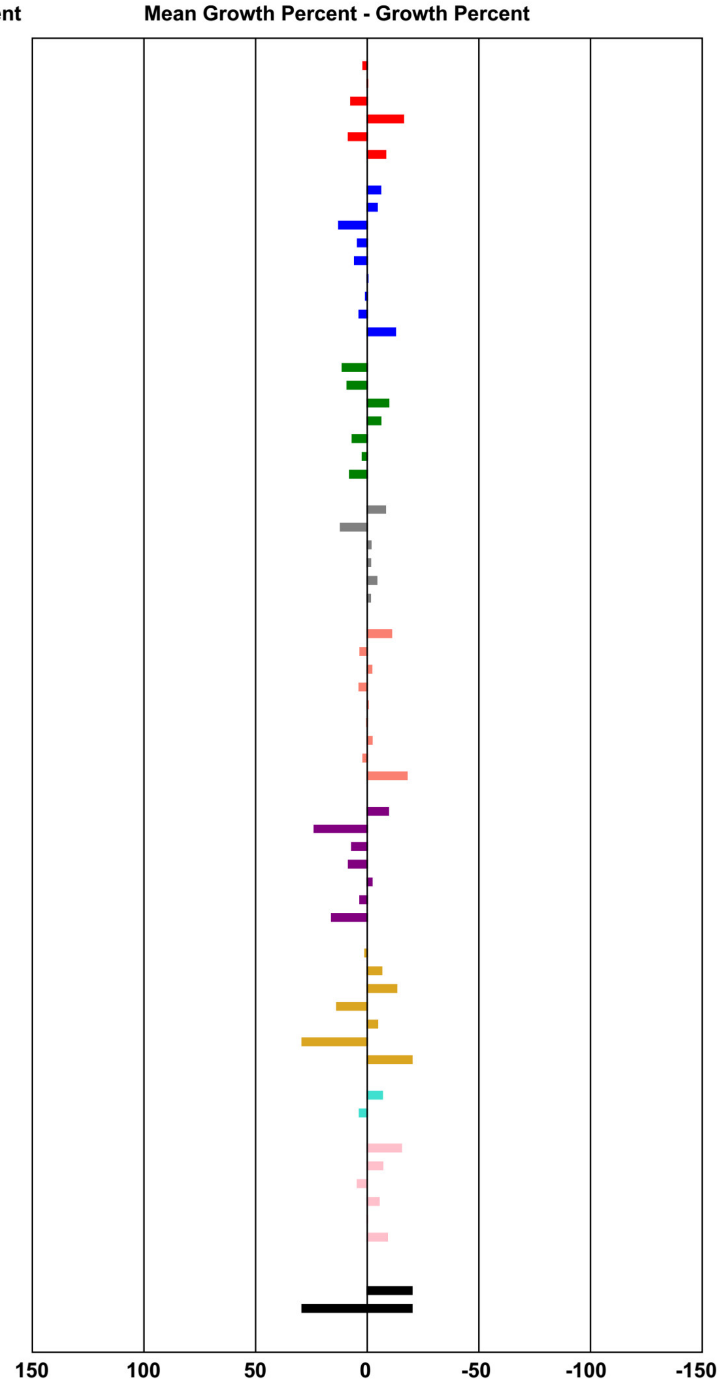

Figure S73. One dose mean graph of **14c**.

3 Docking studies

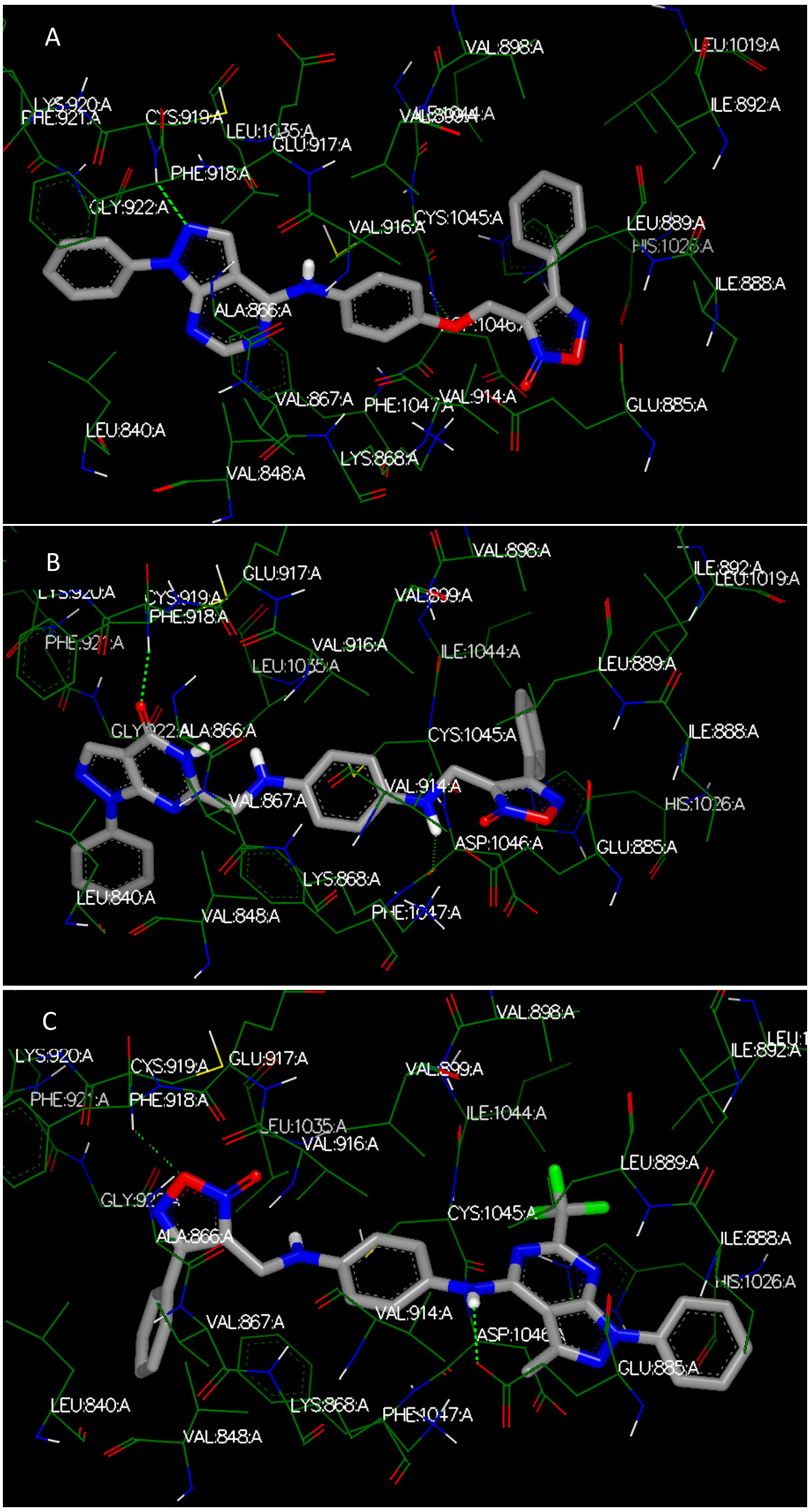

Figure S74. Docking poses of some target compounds inside VEGFR-2 active site: A) Compound **6a**; B) Compound **14a**; C) Compound **12a**.

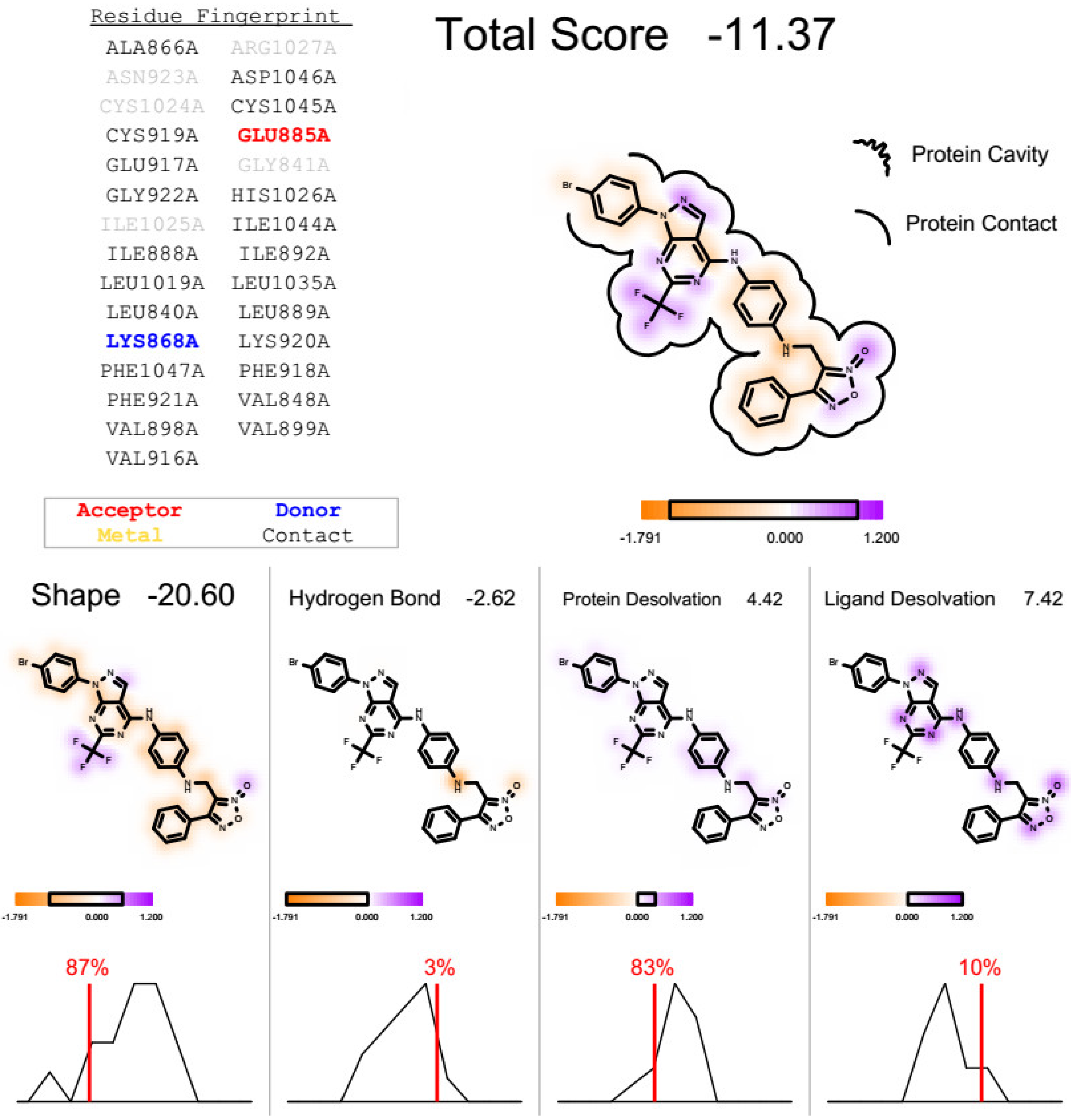

Figure S75. FRED docking report of **12b** in VEGFR-2, showing fitness in the binding pocket and residues in contact.

4 ADMET analysis

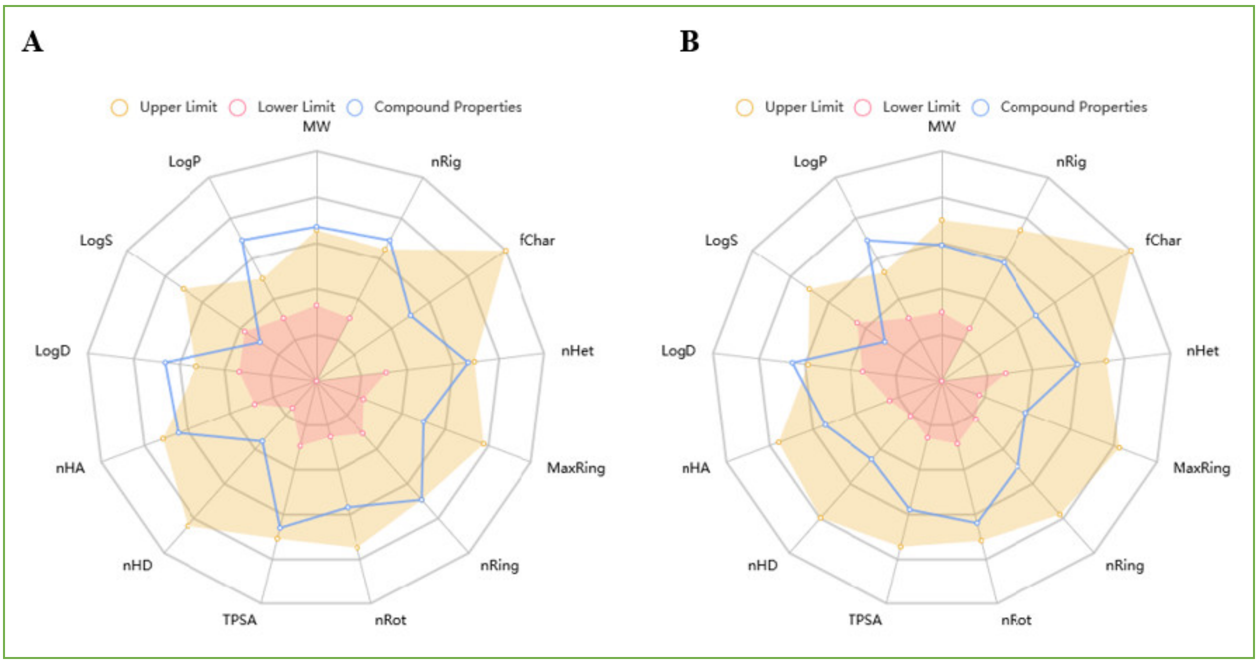

Figure S76. Radar graph of physicochemical properties of A) compound **12b** and B) **sorafenib**, obtained from ADMETlab 2.0.

Table S3. ADMET properties of compound **12b**, predicted using the web-based ADMETlab 2.0 tool.

	Property	Value	Meaning and Preference
**Medicinal Chemistry**	Pfizer Rule	Accepted	log P = 5.918 and TPSA = 123.86
	SAscore (Synthetic accessibility score)	3.406	Easy to be synthesized.
	NPscore (Natural product-likeness score)	-1.455	Within the range (-5 to 5).
	QED (Drug-likeness based on desirability)	0.231	Too complex (<0.34)
**Absorption**	Caco-2 Permeability	-4.977	Optimal (> -5.15 Log unit)
	MDCK Permeability	1.5×10^{-5}	Optimal ($2–20 \times 10^{-6}$ cm/s)
	Pgp inhibitor	0.596	Inhibitor
	Pgp substrate	0.001	Non-substrate
	HIA (Human Intestinal Absorption)	0.009	HIA < 30%
	F20% (20% Bioavailability)	0.001	bioavailability = 20%
	F30% (30% Bioavailability)	0.0	bioavailability = 30%
**Distribution**	PPB (Plasma Protein Binding)	99.67%	Optimal: <90%.
	VD (Volume Distribution)	1.945	Optimal (0.04–20 L/kg)
	BBB (Blood–Brain Barrier) Penetration	0.672	Medium

	Property	Value	Meaning and Preference
**Excretion**	CL (Clearance)	3.038	low (<5 mL/min/kg)
	$T_{1/2}$ (half-life)	0.227	short (<3h)
**Toxicity**	hERG Blockers	0.801	Active
	H-HT (Human Hepatotoxicity)	0.955	Positive
	DILI (Drug-Induced Liver Injury)	0.99	High risk
	AMES Toxicity	0.637	Medium
	Rat Oral Acute Toxicity	0.951	High
	FDAMDD (Maximum Recommended Daily Dose)	0.914	Positive
	Skin Sensitization	0.084	Non-sensitizer
	Carcinogenicity	0.685	Medium
	Eye Corrosion	0.003	Non-corrosive
	Eye Irritation	0.008	non-irritant
	Acute Toxicity Rule (during oral administration)	0 Alerts	No Alerts
	Bioconcentration Factors	1.383	Unit: $-\log_{10}[(mg/L)/(1000*MW)]$
	IGC_{50} (Tetrahymena pyriformis 50% growth inhibition concentration)	5.086	Unit: -log[(mg/L)/(1000*MW)]
**Metabolism**	CYP1A2 inhibitor	Positive	
	CYP1A2 substrate	Negative	
	CYP2C19 inhibitor	Positive	
	CYP2C19 substrate	Negative	
	CYP2C9 inhibitor	Positive	
	CYP2C9 substrate	Negative	
	CYP2D6 inhibitor	Negative	
	CYP2D6 substrate	Negative	
	CYP3A4 inhibitor	Negative	
	CYP3A4 substrate	Negative	
